# Transcriptome of different fruiting stages in the cultivated mushroom *Cyclocybe aegerita* suggests a complex regulation of fruiting and reveals enzymes putatively involved in fungal oxylipin biosynthesis

**DOI:** 10.1186/s12864-021-07648-5

**Published:** 2021-05-04

**Authors:** Axel Orban, Annsophie Weber, Robert Herzog, Florian Hennicke, Martin Rühl

**Affiliations:** 1 Institute of Food Chemistry and Food Biotechnology, Justus Liebig University Giessen, 35392 Giessen, Hesse Germany; 2 International Institute Zittau, Technical University Dresden, 02763 Zittau, Saxony Germany; 3 Project Group Genetics and Genomics of Fungi, Ruhr-University Bochum, Chair Evolution of Plants and Fungi, 44780 Bochum, North Rhine-Westphalia Germany; 4 Fraunhofer Institute for Molecular Biology and Applied Ecology IME Branch for Bioresources, 35392 Giessen, Hesse Germany

**Keywords:** Basidiomycota, C8 oxylipins, Global gene expression analysis, Volatilome, Developmental biology, Multicellular development, Carpophore, Mycelium, Black poplar mushroom, Pioppino, Sesquiterpenes, Dioxygenases

## Abstract

**Background:**

*Cyclocybe aegerita* (syn. *Agrocybe aegerita*) is a commercially cultivated mushroom. Its archetypal agaric morphology and its ability to undergo its whole life cycle under laboratory conditions makes this fungus a well-suited model for studying fruiting body (basidiome, basidiocarp) development. To elucidate the so far barely understood biosynthesis of fungal volatiles, alterations in the transcriptome during different developmental stages of *C. aegerita* were analyzed and combined with changes in the volatile profile during its different fruiting stages.

**Results:**

A transcriptomic study at seven points in time during fruiting body development of *C. aegerita* with seven mycelial and five fruiting body stages was conducted. Differential gene expression was observed for genes involved in fungal fruiting body formation showing interesting transcriptional patterns and correlations of these fruiting-related genes with the developmental stages. Combining transcriptome and volatilome data, enzymes putatively involved in the biosynthesis of C8 oxylipins in *C. aegerita* including lipoxygenases (LOXs), dioxygenases (DOXs), hydroperoxide lyases (HPLs), alcohol dehydrogenases (ADHs) and ene-reductases could be identified. Furthermore, we were able to localize the mycelium as the main source for sesquiterpenes predominant during sporulation in the headspace of *C. aegerita* cultures. In contrast, changes in the C8 profile detected in late stages of development are probably due to the activity of enzymes located in the fruiting bodies.

**Conclusions:**

In this study, the combination of volatilome and transcriptome data of *C. aegerita* revealed interesting candidates both for functional genetics-based analysis of fruiting-related genes and for prospective enzyme characterization studies to further elucidate the so far barely understood biosynthesis of fungal C8 oxylipins.

**Supplementary Information:**

The online version contains supplementary material available at 10.1186/s12864-021-07648-5.

## Background

The formation of fruiting bodies (FBs, basidiomes, basidiocarps) that are in particular formed by species from the Basidiomycota class Agaricomycetes [1] is one of the most complex developmental processes in the fungal life cycle. Depending on the species, this development results in various FB shapes and features (e.g. nutritional mode or FB-specific natural products) the former of which has now been revealed as the major driver of diversification in mushrooms [2]. In a first step, hyphal knots develop as a result of enhanced hyphal branching in defined areas of the vegetative mycelium. The branches in the hyphal knots intertwine successively to initials, being for e.g. *Coprinopsis cinerea* about 1–2 mm in size [3, 4]. Usually, as in the model agaric *Cyclocybe aegerita* (V. Brig.) Vizzini (synonym: *Agrocybe aegerita* (V. Brig.) Singer) [5], cell differentiation takes place in these FB initials, which already becomes evident in late FB initials [6]. Progression of differentiation leads to the formation of bipolar primordia essentially comprising the different ‘tissue’ (more precisely referred to as plectenchyma or plectenchyme in fungi [6–8]) types observed in mature FBs. The subsequent development from differentiated primordia to FBs is mainly due to cell elongation rather than cell differentiation [4]. The formation and maturation of basidiospores and their subsequent release can be highly synchronized, as observed in species with an ephemeral life strategy producing short-lived, autolytic FBs such as the dung-dwelling well-studied model agaric *C. cinerea*. Other Agaricales (‘agarics’) species, representing the more typical case of how meiotic sporulation proceeds in these fungi, lack such a tight synchronization. Sampled FBs of such species contain e.g. spore-forming basidia in various developmental stages at the same time [9]. Asynchronous sporulation is exemplified in the long-lasting FBs of the bracket fungus *Schizophyllum commune*, another important Agaricales model system for mating and fruiting [7, 10–13], where older ‘ripe’ parts of the FB sporulate while younger FB parts still proliferate [3]. Environmental and physiological influences, such as nutrient availability, light and the occurrence of predators, have a great impact on the development of FBs (reviewed in [14]). High concentrations of CO_2_, for example, can suppress fruiting or lead to malformed FBs [15–17]. Furthermore, oxylipins have proven to have an influence on developmental processes in fungi. Recently, Niu et al. demonstrated that 5,8-dihydroxyoctadecadienoic acid induces lateral hyphal branching in *Aspergillus* ssp. with G-protein coupled receptors being involved in the signal transduction [18]. Additionally, gene deletion experiments with inter alia *C. cinerea* and *S. commune* revealed a set of genes that are essential for the proper formation of FBs [7, 10–12, 19–30]. In *S. commune* for example, the deletion of the transcription factor *HOM2* was associated with an enhanced growth of vegetative mycelium unable to develop FBs whereas the deletion of *HOM1* and *GAT1* resulted in the formation of more but smaller FBs with an unusual morphology compared to the wild type [10, 12]. Furthermore, the blue light photoreceptor Wc-1 (also called Dst1 in *C. cinerea*) is essential for the photomorphogenesis of *C. cinerea* and *S. commune* [11, 12, 29]*.* Defects of this gene lead to suppressed primordium maturation with the pileus and stipe tissues at the upper part of the primordium remaining rudimentary [26]. Differential expression of several fruiting-related genes (FRGs) has been observed in different fungal species during FB development including the model agarics *S. commune* [13] and *C. cinerea* [26, 31] as well as the mushrooms *Agaricus bisporus* [32], *Armillaria ostoyae*, *Lentinus tigrinus*, *Phanerochaete chrysosporium*, *Rickenella mellea* [33], *Auriculariopsis ampla* [34], *Hypsizygus marmoreus* [35], *Ganoderma lucidum* [36], *Pleurotus eryngii* [37], *Hericium erinaceus* [38], *Lentinula edodes* [39] and *Flammulina filiformis* [40]. Besides morphological changes during FB development, the odor as a result of released volatile organic compounds (VOCs) is an important characteristic of different fungal species. Several studies revealed that the volatile profile of mushrooms differs depending on the developmental stage [41–49]. In this context, the function of VOCs as ‘infochemicals’ is of special interest since VOCs have proven to influence the behavior of invertebrates and play therefore probably an important role in the fungal life cycle by inter alia repelling fungal predators or attracting insects for the purpose of spore dispersal (reviewed in [50, 51]). Furthermore, C8 VOCs showed regulatory functions in fungi and influence on conidiation and conidia germination in *Penicillium paneum* and *Trichoderma* spp., respectively [52, 53]. Hence, the changes observed in volatilomes of fungi are probably due to the adaption of the organisms to the altering requirements during different developmental stages.

Recently, the changes of the volatilomes in the headspace (HS) of *Cyclocybe aegerita* (syn. *Agrocybe aegerita*), which is a commercially cultivated edible agaricomycete from Europe [5], during different fruiting stages of the dikaryon *C. aegerita* AAE-3 and a set of progeny monokaryons was monitored under nearly natural circumstances applying a non-invasive extraction method [54]. This study revealed drastic changes in the volatile profile across developmental stages. In early stages, alcohols and ketones, including oct-1-en-3-ol and cyclopentanone, were the main substances in the HS of the dikaryon. With ongoing FB development, the VOCs composition differed remarkably and particularly during sporulation. Sesquiterpenes, such as Δ^6^-protoilludene, α-cubebene and δ-cadinene, were the dominant substances detected in the HS at this stage. After sporulation, the amount of sesquiterpenes decreased, while the appearance of additional VOCs, especially octan-3-one, was observed. Despite the notable changes in VOCs profiles and the biological importance of fungal VOCs, overall little is known about the pathways leading to their formation. Even the biosynthesis of volatile C8 oxylipins, such as oct-1-en-3-ol, octan-3-one and oct-1-en-3-one, ubiquitously found in fungi and perceived as ‘typical’ mushroom odors, is so far scarcely understood. It has been proposed that volatile C8 oxylipins are derived from linoleic acid, probably involving lipoxygenases (LOXs), dioxygenases (DOXs) and hydroperoxide lyases (HPLs) in the formation process (reviewed in [51, 55]). However, enzymes clearly linked to volatile C8 oxylipin biosynthesis have been barely identified so far. To tap this hitherto neglected topic of fungal VOCs biosynthesis, we conducted a transcriptomic study with in total seven mycelium- and five FB development stages of *C. aegerita* AAE-3, chosen to be similar with stages sampled in the volatilome study of *C. aegerita* mentioned above [54]. Combining the volatilome and trancriptome data sets and comparing volatile profiles in the HS of *C. aegerita* with transcription patterns of selected genes, we were able to identify enzymes putatively involved in the formation of VOCs in fungi. Especially regarding C8 volatile pathways, we determine for the first time promising candidates responsible for the biosynthesis of fungal VOCs.

## Results

### Fruiting body development in *C. aegerita* in modified crystallizing dishes

**Fig. 1:**
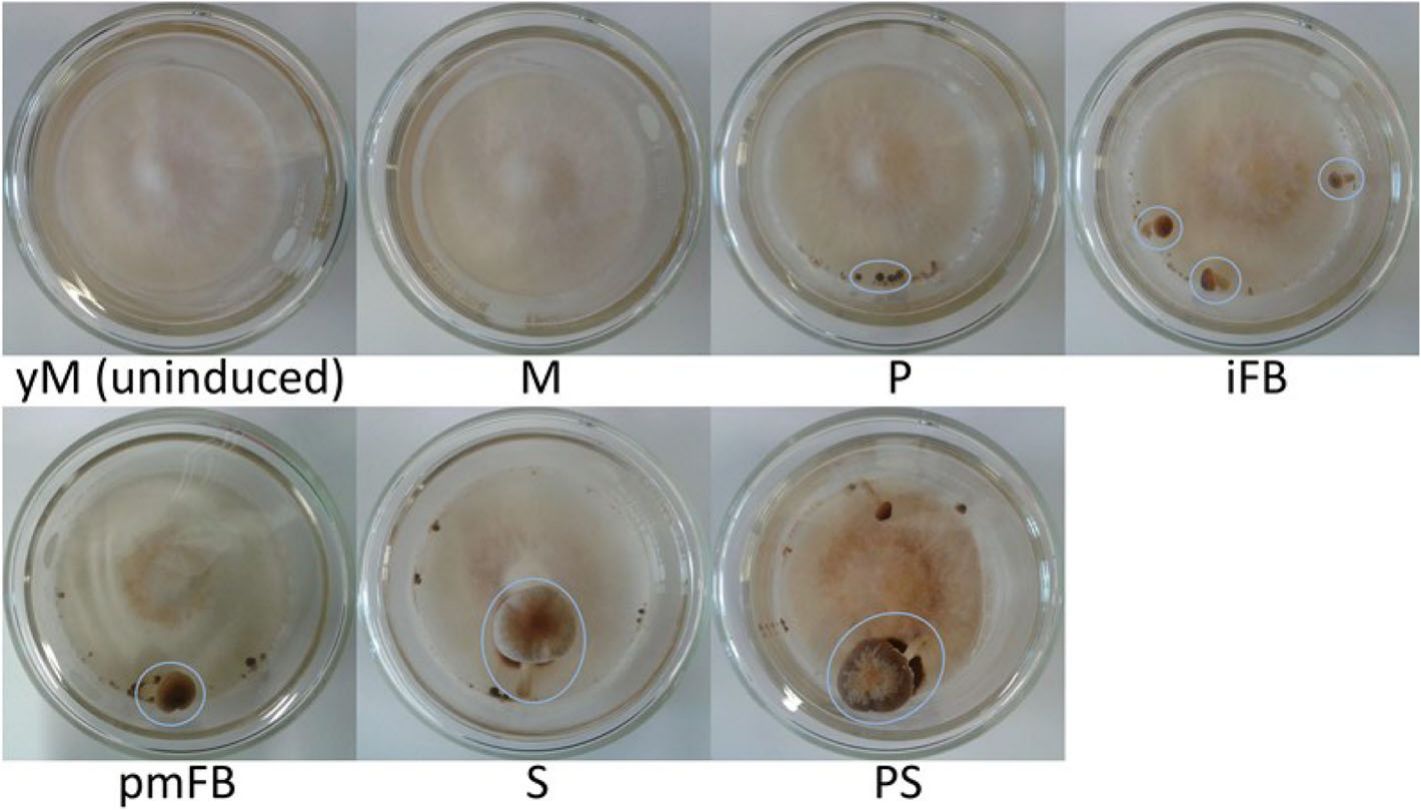
Fruiting body development of *C. aegerita.* Samples were grown at 24 °C in the dark in crystallizing dishes containing 16 mL 1.5% MEA medium and were sealed with Parafilm™. Ten days after inoculation, the Parafilm™ was removed and samples were transferred to a climate chamber (24 °C, 95% rH, 12/12 h day/night rhythm) and cultured for further 18 days. Blue circles indicate examples of FB samples harvested at the designated stage. yM: young (uninduced) mycelium (day 10 post inoculation, p.i.); M: fruiting-primed mycelium (day 14 p.i.); P: primordia (day 18 p.i.); iFB: immature fruiting bodies (day 20 p.i.); pmFB: premature fruiting bodies (day 22 p.i.); S: sporulating mature FB (day 24 p.i.); PS: post sporulation (day 28 p.i.)

The dikaryotic strain *C. aegerita* AAE-3 was able to produce FBs and basidiospores under the chosen cultivation conditions (Fig. 1). By day 18 post inoculation (p.i.), primordia emerged on fruiting-induced mycelium. Further on, they developed into FBs, typically sporulating at day 24 p.i. At the final stage sampled on day 28 p.i., these show first signs of aging, e.g. a moisture-soaked cap margin.

### Differential gene expression during fruiting body development

**Fig. 2:**
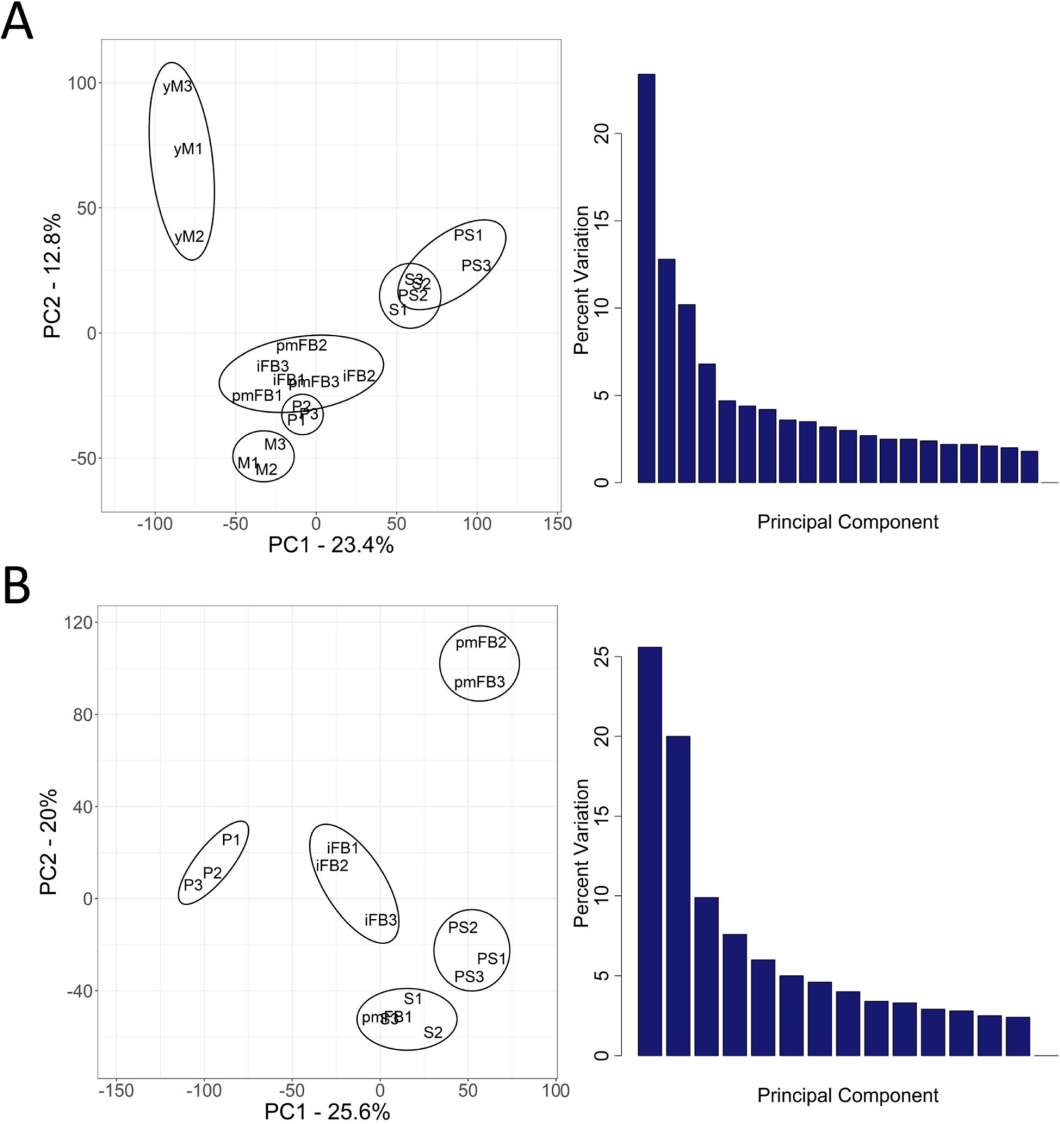
Principal component analysis (PCA) (left) and scree plot (right) for transcriptome data of developmental stages of *C. aegerita*. **a** Transcriptome data of the mycelium samples. PC1 and PC2 covered 36.2% of the data’s original variation. **b** Transcriptome data of the fruiting body samples. PC1 and PC2 covered 45.6% of the data’s original variation. yM: young (uninduced) mycelium (day 10 post inoculation, p.i.); M: mycelium (day 14 p.i.); P: primordia (day 18 p.i.); iFB: immature fruiting bodies (day 20 p.i.); pmFB: premature fruiting bodies (day 22 p.i.); S: sporulation (day 24 p.i.); PS: post sporulation (day 28 p.i.)

To identify changes in the transcriptome during mushroom tissue formation and FB maturation in *C. aegerita*, RNA sequencing of different developmental stages of mycelium and FBs was conducted (Additional file 1). In total, transcripts representing 12,965 of the 14,115 annotated genes were identified (Additional file 2: Table S1, BioProject PRJNA677924, BioSamples 16,789,160 to 16,789,171). A principal component analysis (PCA) was performed for the transcriptomes of mycelium and FBs to highlight similar expression patterns of the different developmental stages (Fig. 2). For the mycelium samples, the first two principal components covered 36.2% of the data’s original variation and developmental stages clustered roughly in three groups (Fig. 2a). The transcriptome of the young mycelium samples, which were taken prior to the day night shift all other samples have been exposed to, formed an individual cluster. The following four FB developmental stages (fruiting-primed mycelium to mature FBs) clustered together as well as the transcriptomes of the last two developmental stages (sporulation and post sporulation). The performed Friedman test revealed significant differences (*p* = 5.448e^− 10^) between the transcriptomes of the different developmental stages of the mycelium. The Wilcoxon-Nemenyi-McDonald-Thompson test, used as the post hoc analysis of the Friedman test, showed that transcriptomes of sporulation and post sporulation samples differed significantly (*p* < 0.05) from samples of all other stages, but were similar to each other. Additionally, the transcriptome of young mycelium samples differed significantly from transcriptomes of fruiting-primed mycelium and primordia samples. For the FB samples, the first two principal components represented 45.6% of the data’s original variation with all five developmental stages forming individual clusters (Fig. 2b). The performed Friedman test revealed significant differences (*p* = 3.162e^− 13^) between the transcriptomes of the different developmental stages of the FBs. The Wilcoxon-Nemenyi-McDonald-Thompson test displayed that primordia, immature FB and premature FB samples each showed significant differences (*p* < 0.05) regarding their transcriptomes compared to samples of the other stages, with pmFB1 being a striking exception sharing consistent features with sporulating FBs. Compared to the other premature FBs, pmFB1 samples were probably further developed but without showing visible signs of sporulation. Additionally, the sporulation and post sporulation stages did not differ significantly amongst themselves.

A highly interesting difference between two mycelial samples occurred during maturation of FBs. From day 22 to day 24 amongst genes having at least a read count of 100 in the mycelium samples, 66 genes showed a > 5-fold decrease (Additional file 2: Table S2) and 117 genes a > 5-fold increase (Additional file 2: Table S3). The deduced protein sequences of these regulated genes were analyzed by means of BLAST (tblastn) using characterized proteins present in the UniProt database. In total, 35 sequences of the downregulated and 75 sequences of the upregulated genes could be functionally allocated. Of these, most upregulated genes (15 of 75) are related to the mevalonate pathway and the sesquiterpenoid clusters, whereas the down-regulated genes mainly code for six putative hydrophobins and other fruiting-related genes (FRGs). Thus, we focus on the FRGs as well as on genes that are involved in the biosynthesis of volatile compounds mainly produced during fructification and sporulation.

### Transcription of fruiting-related genes (FRGs)

**Fig. 3:**
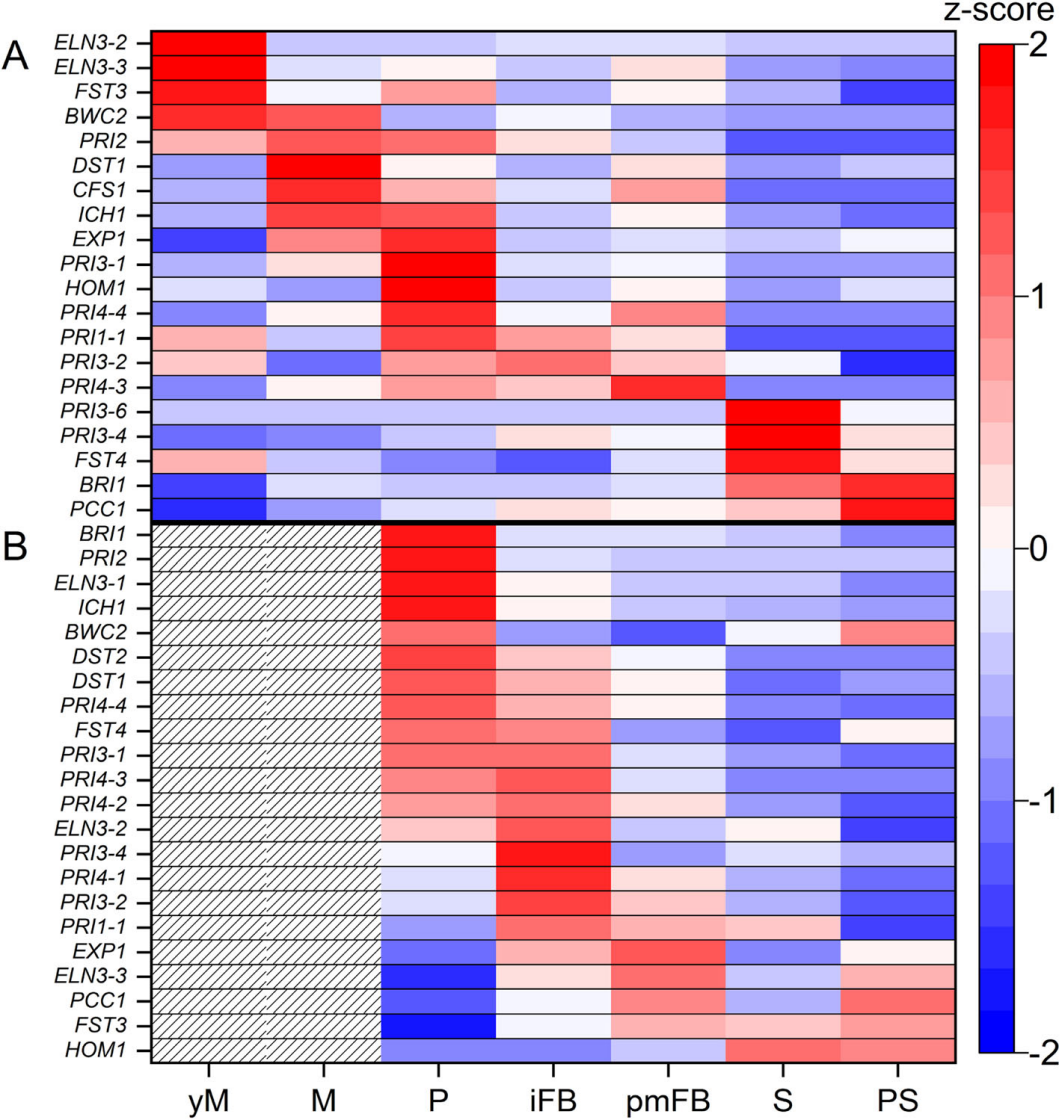
Transcription of putative *C. aegerita* homologs of fruiting-related genes (FRGs) in mycelium (**a**) and in mushroom tissue (**b**) during different developmental stages. NRC per gene were transformed to z-score values (respective scale to the right) whereby only genes were considered showing maximum transcription levels higher than 25 NRC. Red colors indicate transcriptional upregulation while blue colors represent downregulation. White colors indicate a z-score of zero and hatched areas show an absence of sampling due the non-applicability. yM: young (uninduced) mycelium (day 10 post inoculation, p.i.); M: mycelium (day 14 p.i.); P: primordia (day 18 p.i.); iFB: immature fruiting bodies (day 20 p.i.); pmFB: premature fruiting bodies (day 22 p.i.); S: sporulation (day 24 p.i.); PS: post sporulation (day 28 p.i.). FRGs (in order of appearance in the panels **a** and **b**): *ELN3–2* (AAE3_06792), *ELN3–3* (AAE3_13318), *FST3* (AAE3_09009), *BWC2* (AAE3_13841), *PRI2* (AAE3_02445), *DST1* (AAE3_10538), *CFS1* (AAE3_01819), *ICH1* (AAE3_04768), *EXP1* (AAE3_02324), *PRI3–1* (AAE3_14114), *HOM1* (AAE3_03904), *PRI4–4* (AAE3_04667), *PRI1–1* (AAE3_04306), *PRI3–2* (AAE3_14115), *PRI4–3* (AAE3_04665), *PRI3–6* (AAE3_13216), *PRI3–4* (AAE3_14116), *FST4* (AAE3_11357), *BRI1* (AAE3_08826), *PCC1* (AAE3_01481), *ELN3–1* (AAE3_00364), *DST2* (AAE3_02725), *PRI4–2* (AAE3_04675), *PRI4–1* (AAE3_04684)

In the genome sequence of *C. aegerita*, Gupta et al. [56] identified an array of putative homologs of genes confirmed to play a role in fruiting of model agarics. The transcription levels of these FRGs were analyzed, whereby only genes were considered showing maximum transcription levels higher than 25 normalized read counts (NRC) (Fig. 3). Structurally according to Gupta et al. [56], these FRGs can be grouped into three major groups. The largest group of putative *C. aegerita* FRGs encodes for the transcription factors Bri1, Bwc2, C2H2, Exp1, Fst3, Fst4, Gat1, Hom1, Hom2 and Pcc1, which had been described from *S. commune* and *Coprinopsis cinerea* [10, 12, 20, 28, 29], originally. A second group of FRGs annotated to the *C. aegerita* AAE-3 genome sequence [56] includes the genes *CFS1*, *DST1*, *DST2*, *ELN3* and *ICH1*, encoding for proteins with diverse functions, e.g. blue light perception or a putative role in cell wall carbohydrate production, all derived from putatively orthologous genes of *C. cinerea* [21, 25–27, 30]. A third group consists of four previously annotated genes [56], *PRI1* to *PRI4,* proven to be transcriptionally upregulated in primordia of the wild type strain *C. aegerita* SM51 also known as WT-1 [19, 22–24].

Regarding their expression patterns during fruiting, these very different FRGs were clustered according to the developmental phase and hyphal context when and where they were mainly expressed (Fig. 3). In total, four larger cohorts can be distinguished from another: two in the mycelium and two in the mushroom tissue. In the mycelium, a first cohort comprises genes reaching expression maxima already early in mycelial stages until primordia developed. It includes the genes *ELN3–2*, *ELN3–3*, *FST3*, *BWC2*, *PRI2*, *DST1*, *CFS1*, *ICH1*, *EXP1*, *PRI3–1*, *HOM1*, *PRI4–4* and *PRI1–1*. Four of them, namely *ELN3–3*, *CFS1*, *PRI1* and *PRI2,* have elevated expression levels spanning multiple stages (Fig. 3a). The second group consists of genes whose expression peaked in mycelium during the formation of post-primordial fruiting stages: *PRI3–2*, *PRI4–3, PRI3–6*, *PRI3–4*, *FST4*, *BRI1* and *PCC1*.

In FBs, the first cohort comprises genes which reached an expression maximum in early fruiting stages. The genes *BRI1*, *PRI2*, *ELN3–1*, *ICH1*, *BWC2*, *DST2*, *DST1*, *PRI4–4*, *FST4* and *PRI3–1* revealed expression maxima in *C. aegerita* primordia, some of them such as *DST1*, *FST4* and *PRI3–1* showed a continuously high expression in subsequent FB stages (Fig. 3b). Other genes in this group, including *PRI4–3*, *PRI4–2*, *ELN3–2*, *PRI3–4*, *PRI3–2*, *PRI4–1* and *PRI1–1*, displayed highest expression in immature FBs with *PRI4–3*, *PRI3–2* and *PRI1–1* revealing remarkably high expression in previous and subsequent stages. The second gene cohort is formed by the genes *EXP1*, *ELN3–3*, *PCC1*, *FST3 and HOM1* revealing high expression in late fruiting stages. All genes in this cohort showed expression maxima within FB tissue samples which only span two stages, except for *FST3* which revealed constantly elevated transcription in FB tissue until reaching the post-sporulation stage (Fig. 3b). Moreover, correlations between the expression of individual FRGs in FB tissue and mycelium samples were investigated. Generally, formation of clusters of strongly positive correlated genes was more apparent in FRGs expressed in FB samples (Additional file 3: Figures S2 and S3).

### RT-qPCR-based confirmation of expression values of selected candidate genes

Expression of four predicted *C. aegerita* orthologs of known transcription factor-encoding FRGs from *S. commune* and *C. cinerea* was additionally monitored via quantitative real-time reverse transcription-PCR (RT-qPCR) (Additional file 3: Figure S4; 2-fold expression change as cut-off) to get a further hint in how far a predicted ortholog might work in the same way during fruiting of *C. aegerita*. The RT-qPCR-monitored expression pattern of *HOM1* (Additional file 3: Figure S4A) showed a transcriptional induction from the FB initial stage through the primordium and immature FB stage which confirmed its expression pattern detected within the RNA-seq analysis (see Fig. 3), at least for the congruently assessed development stages. The expression profile of *GAT1* monitored by RT-qPCR (Additional file 3: Figure S4B) proved that this FRG is indeed differentially expressed during fruiting of *C. aegerita* AAE-3, despite its sub-threshold expression values in the RNA-seq analysis above. The transcriptional induction of *DST1* in primordia and immature FB cap tissue over its expression in young mycelium (Additional file 3: Figure S4C) was congruent with its high expression in primordia and immature FBs over young mycelium detected by the RNA-seq analysis (see Fig. 3). Eventually, the RT-qPCR-monitored expression pattern of *BWC2* (Additional file 3: Figure S4D) generally confirmed the transcriptional induction of this gene during fruiting (from the primordium stage on) compared to its expression in mycelial stages, at least for the assessed development stages compared to the ones assessed by the RNA-seq analysis (see Fig. 3).

### Elucidation of aroma related biosynthesis pathways during development of *C. aegerita*

**Fig. 4:**
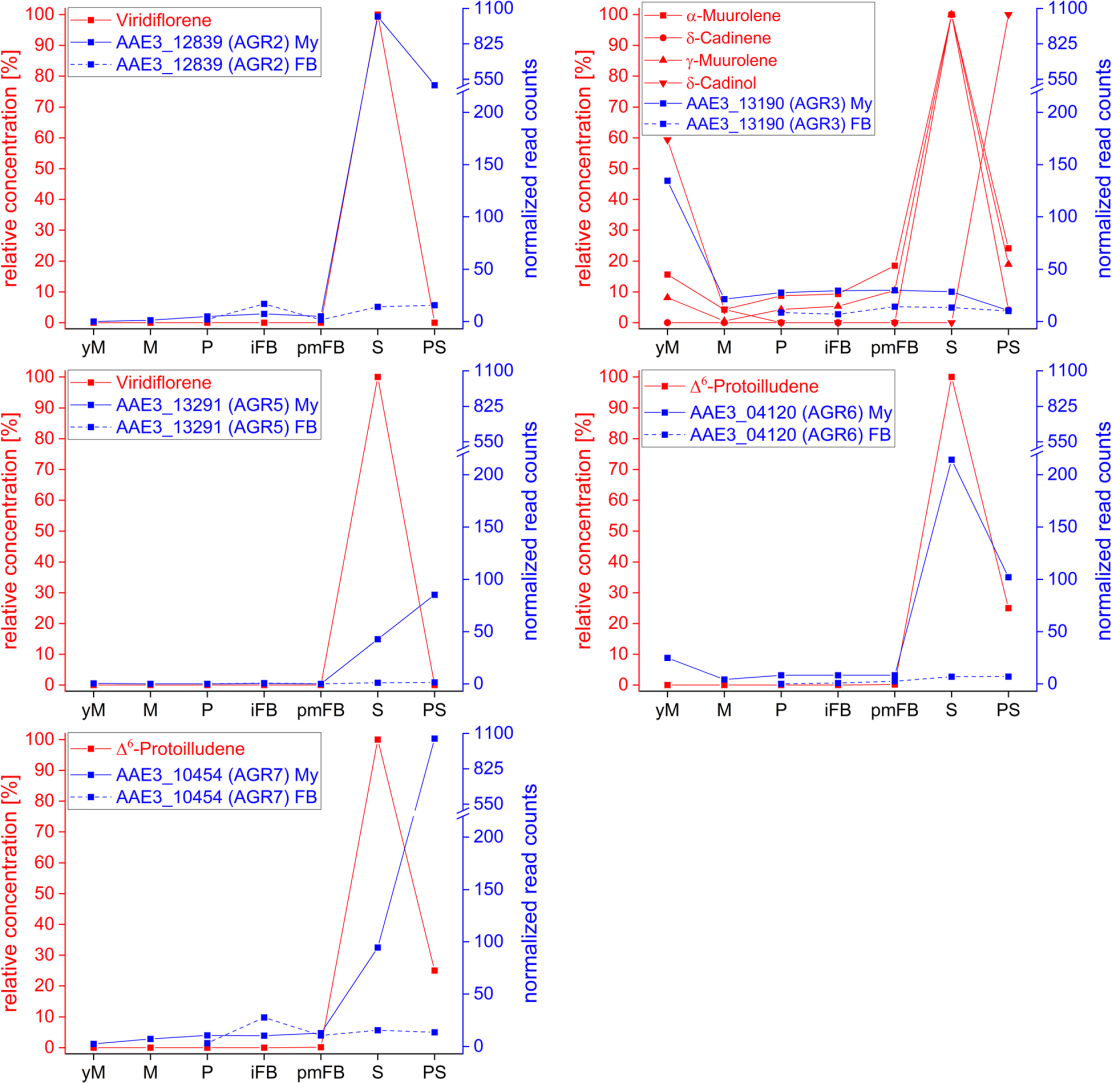
Transcription levels of genes coding for the sesquiterpene synthases (STSs) Agr2, Agr3, Agr5, Agr6 and Agr7 (blue) in the mycelium (My) and in fruiting bodies (FB) during different developmental stages of *C. aegerita* as well as the relative concentrations of the corresponding sesquiterpenes (red) in the HS of *C. aegerita* [54]. yM: young (uninduced) mycelium (day 10 post inoculation, p.i.); M: mycelium (day 14 p.i.); P: primordia (day 18 p.i.); iFB: immature fruiting bodies (day 20 p.i.); pmFB: premature fruiting bodies (day 22 p.i.); S: sporulation (day 24 p.i.); PS: post sporulation (day 28 p.i.)

To take up results of a recently published work on VOCs produced by *C. aegerita* during different developmental stages [54], this transcriptome study should help to elucidate biosynthesis pathways of VOCs such as sesquiterpenoids and oxylipins in *C. aegerita*. The large diversity of sesquiterpenes and other terpenes is derived from only two precursors, dimethylallyl diphosphate and isopentenyl diphosphate, which in fungi are produced from acetyl-CoA by means of the mevalonate pathway [57]. Genes coding for enzymes of the mevalonate pathway were identified in the *C. aegerita* genome by means of BLAST search using amino acid sequences of already characterized fungal analogs. Generally, the expression of enzymes involved in the mevalonate pathway were upregulated in the mycelium during sporulation and post sporulation, whereas in FBs, the transcription of these enzymes was rather higher in early stages of development (Additional file 4: Figure S5). This scenario is especially true for the farnesyl pyrophosphate synthase gene. Its corresponding enzyme provides farnesyl pyrophosphate, which is cyclized by sesquiterpene synthases (STSs) into a wide range of sesquiterpenes [58]. The genome of *C. aegerita* contains 11 genes coding for STSs [59]. Of these, nine gave rise to one or more sesquiterpenes after transformation into *E. coli* [59] (Additional file 4: Figure S5). The comparison of the transcription levels of the different STSs revealed remarkable differences, also strongly depending on sample type and developmental stage (Fig. 4, Additional file 4: Figure S6). Generally, the maximum transcription levels of the STSs were noticeably lower in the examined FB stages than in the mycelial samples, never exceeding 50 NRC in the FB samples. When comparing the occurrence of Δ^6^-protoilludene, the most dominant VOC in the HS of *C. aegerita* AAE-3 during sporulation at day 24 p.i [54]. (Fig. 4), with the gene expression values of the two known Δ^6^-protoilludene synthases Agr6 and Agr7 [59], the transcription pattern of *AGR6* (AAE3_04120) in the mycelium perfectly reflects the occurrence of Δ^6^-protoilludene. In contrast, *AGR7* (AAE3_10454) showed the highest transcription levels in the mycelium after sporulation at day 28 p.i. when Δ^6^-protoilludene production already decreased. *AGR2* (AAE3_12839), which is associated with the production of viridiflorene [59], peaked simultaneously with the highest amount of viridiflorene at day 24 p.i. during sporulation revealing a 200-fold expression upregulation compared to day 22. In contrast, AAE3_13291, the gene coding for Agr5, showed the highest transcription level later on at day 28 p.i. It is worth mentioning that *AGR3* (AAE3_13190), which codes for a promiscuous STS involved in the biosynthesis of α-muurolene, δ-cadinene, γ-muurolene and δ-cadinol, was the only examined STS having its maximum transcription level at an early developmental stage at day 10 p.i., where some of its possible products also showed a slight maximum (Fig. 4). Nevertheless, throughout the peak maxima of α-muurolene, δ-cadinene, γ-muurolene and δ-cadinol during sporulation *AGR3* transcripts stayed at a low level. In contrast to the STSs mentioned above, genes coding for Agr1, Agr4, Agr8 and Agr9 were barely expressed in *C. aegerita* under the applied experimental conditions, neither in the mycelium nor in the FB samples (Additional file 4: Figure S6).

**Fig. 5:**
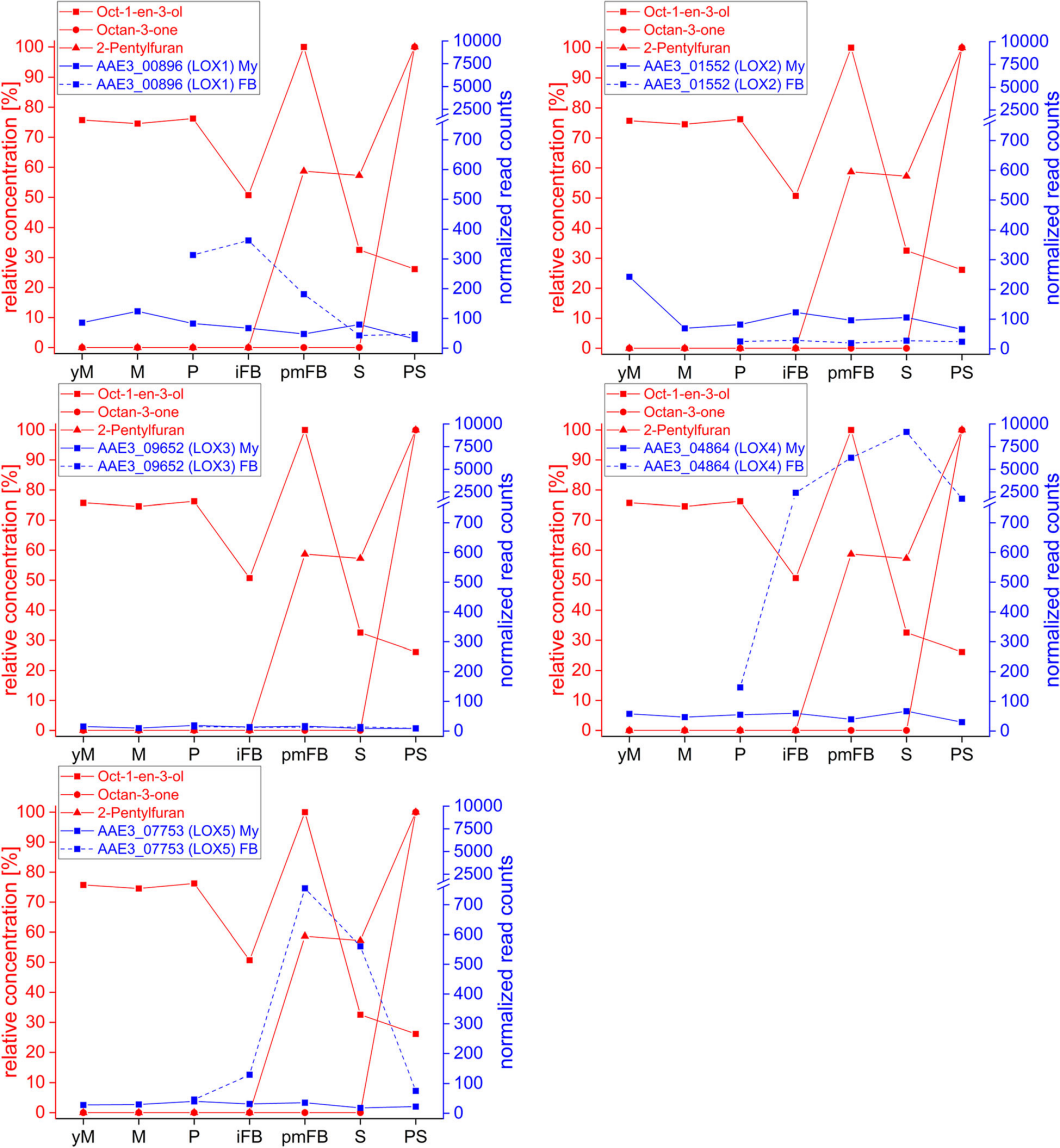
Transcription levels of the lipoxygenases *LOX1*–*5* (blue) in the mycelium (My) and in fruiting bodies (FB) during different developmental stages of *C. aegerita* as well as the relative concentrations of volatile oxylipins (red) in the HS of *C. aegerita* [54]. yM: young (uninduced) mycelium (day 10 post inoculation, p.i.); M: mycelium (day 14 p.i.); P: primordia (day 18 p.i.); iFB: immature fruiting bodies (day 20 p.i.); pmFB: premature fruiting bodies (day 22 p.i.); S: sporulation (day 24 p.i.); PS: post sporulation (day 28 p.i.)

In addition to the sesquiterpenes, the biosynthesis of oxylipins in fungi is of special interest. Biosynthesis of volatile fungal oxylipins, including the typical mushroom C8 aroma compounds such as oct-1-en-3-ol, octan-3-one and octan-3-ol, but also other oxylipins like 2-pentylfuran, ubiquitously found in fungi, is yet barely understood. Oxylipins derive from oxidized fatty acids or substances originating therefrom [60]. Linoleic acid, a product of the fatty acid synthesis and further processing steps (see Additional file 5), serves as a precursor for fungal (volatile) oxylipins, involving presumably lipoxygenases (LOXs), dioxygenases (DOXs), hydroperoxide lyases (HPLs), alcohol dehydrogenases (ADHs) and ene-reductases in the formation process [49, 61–65] (Additional file 6: Figure S8). Interestingly, the composition of the three volatile oxylipins detected in the HS of *C. aegerita* AAE-3 varied remarkably depending on the developmental stages [54] (Fig. 5). Taking these variations into account, identification of unknown enzymes involved in fungal volatile oxylipin formation by analyzing correlation patterns would be a favorable approach. Therefore, transcriptome and volatilome data analysis were performed in R, revealing high correlations between the expression patterns of certain genes and the occurrence of oxylipins. Nonetheless, even the application of a stringent Spearman’s rank correlation coefficient threshold (ρ = 0.7) resulted in too many hits for an efficient identification of genes putatively involved in volatile oxylipin biosynthesis (e.g. for oct-1-en-3-ol about 1000 genes with a matching expression profile were found in the FB samples) illustrating that a correlation does not necessarily means that a causal relation exists. Accordingly, BLAST searches were performed (for details see Methods) to reduce the number of genes coding for enzymes putatively involved in volatile oxylipin biosynthesis. For the most promising candidates, gene expression patterns were matched to the volatile oxylipin profiles to reveal putatively relevant connections.

The first step towards oxylipins is the oxygenation of fatty acids, mainly linoleic acids, by LOXs. The maximum transcription levels of the LOX genes in *C. aegerita* were noticeably higher in FBs than in the mycelium (Fig. 5), except for *LOX2* (AAE3_01552) and *LOX3* (AAE3_09652), of which the latter was barely expressed. The by far highest transcription level amongst all LOX genes was detected for *LOX4* (AAE3_04864) showing a successively upregulation in FB stages during development and peaking during sporulation at day 24 p.i. that lead to a 62-fold upregulation compared to the primordia stage. In contrast, in the mycelium the transcription of *LOX4* (Fig. 5) was remarkably less pronounced showing 135-fold less expression during sporulation compared to FBs. In mature FBs, *LOX5* (AAE3_07753) displayed the second highest transcription level of all LOX genes showing a transcription pattern quite similar to the occurrence of oct-1-en-3-ol in the HS of *C. aegerita*. Conversely, *LOX1* (AAE3_00896) revealed its highest transcription levels in early FB stages, peaking in immature FBs and decreasing afterwards.

In contrast to all other LOX genes, *LOX2* (AAE3_01552) displayed a remarkably higher transcription level in the mycelium revealing a 10-fold higher maximum expression at day 10 p.i. compared to the quite constant transcription levels in FB stages.

**Fig. 6:**
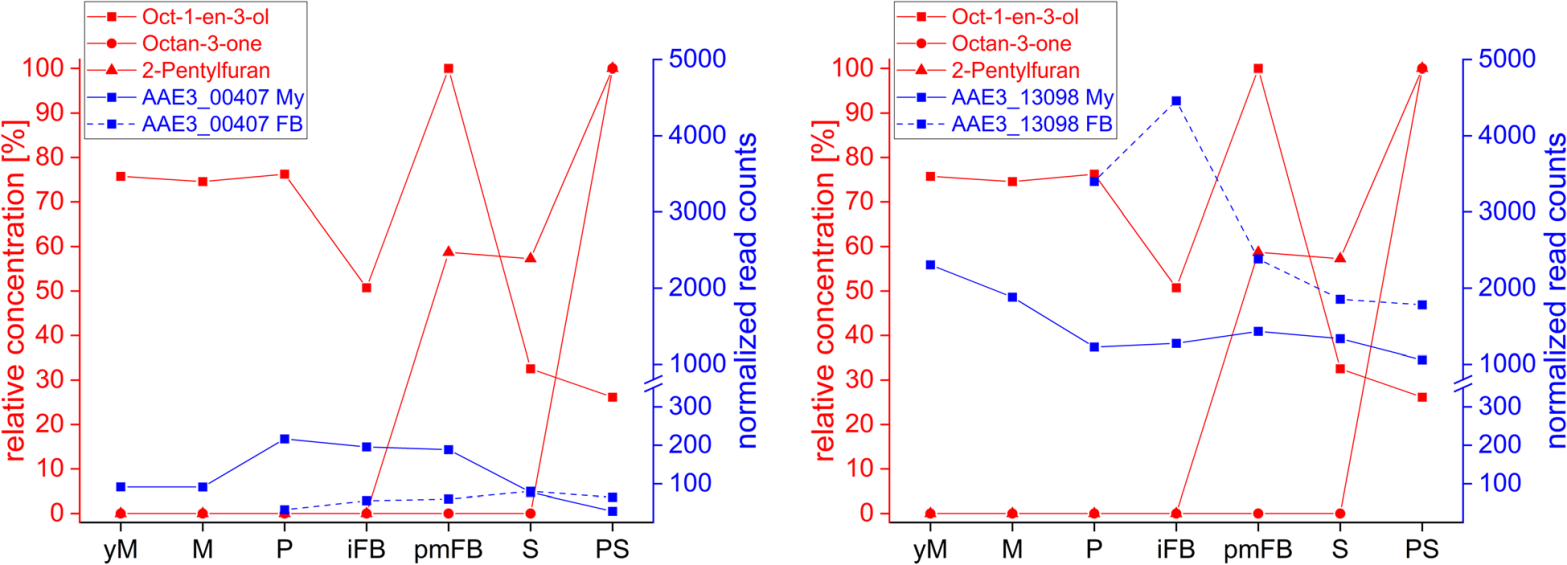
Transcription levels of the putative dioxygenase (DOX) genes AAE3_00407 and AAE3_13098 (blue) in the mycelium (My) and in fruiting bodies (FB) during different developmental stages of *C. aegerita* as well as the relative concentrations of volatile oxylipins (red) in the HS of *C. aegerita* [54]. yM: young (uninduced) mycelium (day 10 post inoculation, p.i.); M: mycelium (day 14 p.i.); P: primordia (day 18 p.i.); iFB: immature fruiting bodies (day 20 p.i.); pmFB: premature fruiting bodies (day 22 p.i.); S: sporulation (day 24 p.i.); PS: post sporulation (day 28 p.i.)

Besides the activity of LOXs on linoleic acid, DOXs might also play a crucial role in the formation of fungal volatile oxylipins [49] (Additional file 6: Figure S8). Two putative DOXs genes were found in the genome of *C. aegerita* by means of BLAST search using amino acid sequences of already characterized ascomyceteous DOXs. Interestingly, the transcription of the putative DOX gene AAE3_00407 was upregulated in the mycelium of early fruiting stages (2-fold change), whereas the transcription in the FBs remained comparably low (Fig. 6). In contrast, the expression of the putative DOX gene AAE3_13098 was high in young fruiting stages, peaking with nearly 4500 NRC in immature FBs, thereafter showing a 2.5-fold transcription decrease, along with the dropping amount of oct-1-en-3-ol in the HS, towards late FB stages (Fig. 6). It is worth mentioning that AAE3_13098 revealed also high expression (about 2000 NRC) in young mycelium stages as well as in later mycelium stages with an almost constant expression level of slightly above 1000 NRC.

It is likely that, analogous to plants, fungi have HPLs catalyzing the cleavage of hydroperoxide molecules into a C8 body that is subsequently converted by oxidoreductases into different VOCs (Additional file 6: Figure S8). The most prominent expression of a putative HPL was revealed by its encoding gene AAE3_05330 (3500 NRC) in late FB stages with a 2.3-fold increase of the transcription level compared to primordia stages and with high expression in the mycelium (1000 NRC at day 14 p.i.) (Additional file 6: Figure S9). For AAE3_09203, the highest expression was observed in immature FBs (2000 NRC), whereas in the mycelium the transcription of AAE3_09203 was remarkably lower revealing 16-fold less expression at this stage. Interestingly, in FB stages as well as in the mycelium, the course of expression of AAE3_09203 was comparable to the transcription of the putative DOX AAE3_13098. AAE3_09218 showed high expression in primordia (1500 NRC) with a 3-fold expression downregulation towards later stages. In the mycelium, the highest transcriptions were observed for AAE3_12835 (2000 NRC) and AAE3_04119 (1600 NRC) during sporulation but both with comparable low expression (17-fold and 58-fold less expression, respectively) in FB stages. In contrast, AAE3_06380 revealed quite constant transcription levels (about 1000 NRC) in the mycelium as well as in FB stages.

In addition to the oxygenation of linoleic acid by means of LOXs or DOXs and the subsequent cleavage to C8 oxylipins by means of HPLs, the enzymatic conversion of mentioned C8 oxylipins might play an important role in the formation of C8 VOCs in fungi. In this context, ADHs and ene-reductases might play an important role, explaining the observed decrease of oct-1-en-3-ol during sporulation and, thereafter, the increase of octan-3-one in the HS of *C. aegerita* [54]. Putative ADHs and ene-reductases were identified in the genome of *C. aegerita* by means of BLAST search using amino acid sequences of characterized ADHs and ene-reductases*.* The transcription levels of putative ADHs and ene-reductases were analyzed in the mycelium and in FB stages (Additional file 6: Figures S10 and S11). In general, most genes coding for putative ADHs showed transcription levels under 500 NRC in the mycelium and in FB tissue samples. Interestingly, the expression of AAE3_00054, AAE3_10620 and AAE3_12451 successively increased in FB stages, showing during sporulation high transcription levels with a 30-fold, 4-fold and 72-fold expression upregulation, respectively, compared to primordia stages and peaking concurrently with a low level of oct-1-en-3-ol content in the HS of *C. aegerita*. Comparable transcription, with over 2500 NRC during sporulation, was observed for AAE3_05375 in the mycelium, already displaying a high expression (900 NRC) in the young mycelium at day 10 p.i. Remarkably, the expression of AAE3_02583 reached, with nearly 3000 NRC, a high transcription level after sporulation at day 28 p.i., displaying, compared to expression during sporulation, a 23-fold upregulation concomitant with the appearance of octan-3-one in the HS of *C. aegerita*.

The genome of *C. aegerita* revealed some interesting putative ene-reductases (Additional file 6: Figure S11). The by far highest expression was revealed by AAE3_13549 in mature FBs (3300 NRC) as well as in FBs during sporulation (7900 NRC) and after sporulation (4750 NRC), representing a 34-fold upregulation during sporulation compared to primordia stages. Interestingly, the expression pattern of AAE3_13549 showed remarkable similarity with the transcription of the highly expressed putative ADHs AAE3_00054, AAE3_10620 and AAE3_12451 in FB stages. In contrast, the maximum transcription level of AAE3_13549 in the mycelium during sporulation was quite low, showing, compared to FBs, 24-fold less expression. In contrast to AAE3_13549, other putative ene-reductases were only slightly upregulated during late fruiting stages compared to early developmental stages. Such was observed during sporulation inter alia for AAE3_00194, with a 2-fold higher transcription level in the mycelium and a 1.5-fold expression upregulation in FB stages, or for AAE3_02355, with a 5-fold higher expression in the mycelium. It is worth mentioning that of the putative ene-reductases belonging to the OYE (old yellow enzyme) family only AAE3_09471 showed a maximum expression higher than 300 NRC, with about 400 NRC in sporulating FBs.

## Discussion

In this study, we conducted the first comparative transcriptome analysis of *C. aegerita* comprising the most important life stages of *C. aegerita* after successful mating and dikaryotization including seven mycelium and five FB developmental stages, for the first time also considering samples of the mycelium during fructification. A previous study dealing with the transcriptome of *C. aegerita* based on a de novo assembly of expressed sequences tags only compared one mycelium developmental stage with one fruiting stage without specifying the time of sampling [66]. Our transcriptomic data are in good agreement with results of other transcriptome studies on different developmental stages of other fungi of the phylum Basidiomycota regarding number of transcripts and differentially expressed genes (DEGs) [33, 37, 67–69]. For instance, Song et al. found 11,675 unique transcripts [70] of 13,028 predicted genes [71] by RNA-Seq analysis of mycelium and mature FBs of *Lentinula edodes*.

The differences of the transcription pattern between the FB stages in *C. aegerita* was expected (Fig. 2b). However, the differences within the fungal mycelium samples was astonishing (Fig. 2a). The beginning of the day/night shift from day 10 onwards explains the extreme alteration in the transcriptome between day 10 and day 14, but not the variation in the transcripts within the mycelial samples occurring during sporulation of the FBs (Fig. 2). Multiple genes are responsible for this alteration that can be assigned as e.g. FRGs or as genes involved in biosynthetic pathways of VOCs.

### Fruiting-related genes (FRGs)

**Fig. 7:**
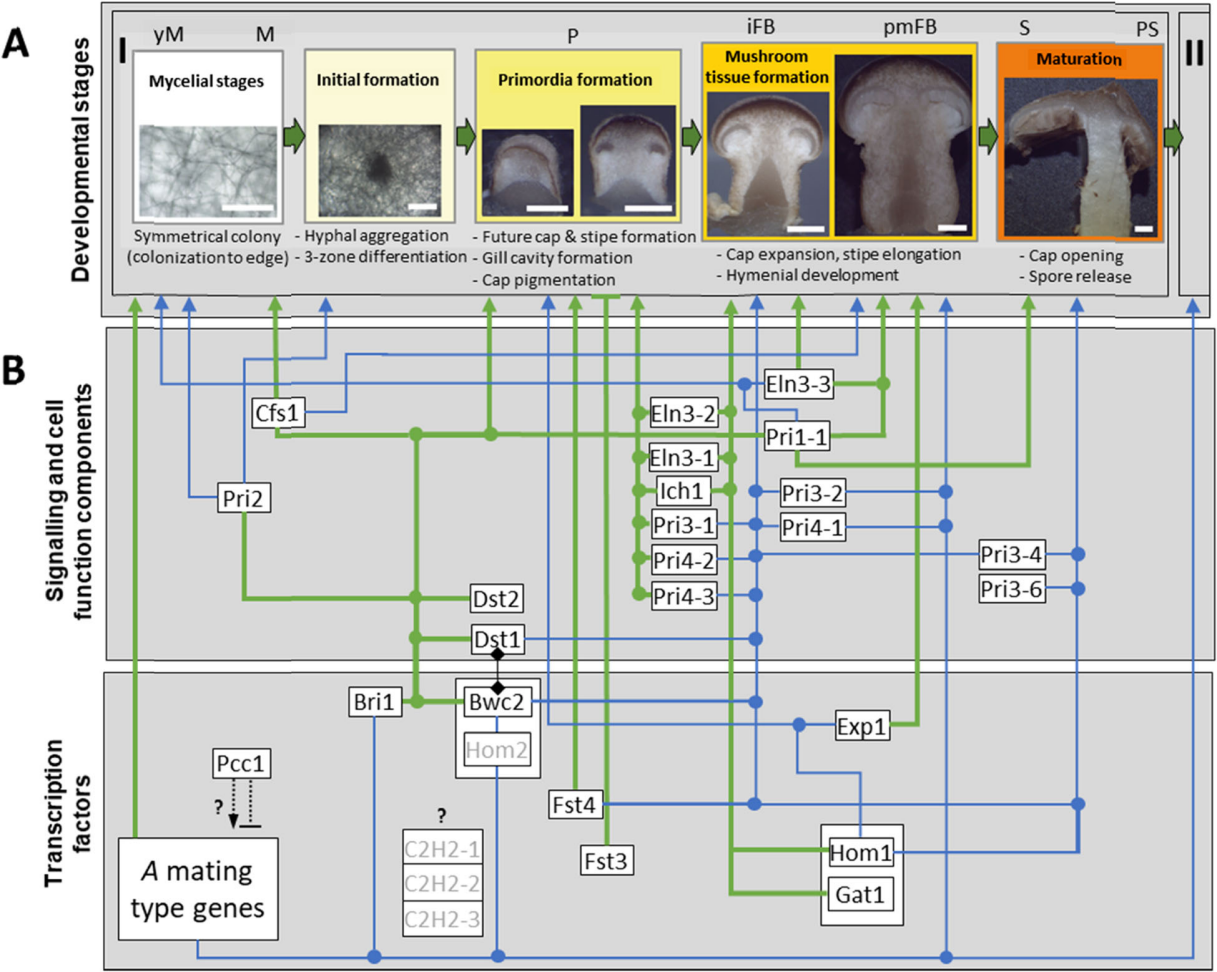
Hypothetic roles of *C. aegerita* orthologs of “classic” model agaric fruiting-related genes (FRGs) from *S. commune*, *C. cinerea* and *C. aegerita* SM51 in FB development of *C. aegerita* AAE-3. **a** Landmarks during fruiting of *C. aegerita* AAE-3 in the fruiting regime of Herzog et al. [6] as monitored by handcuts. The five stages of this process are shown in white to orange boxes. Scale bars represent 100 μm (white box), 400 μm (light yellow box), 0.5 mm (yellow box), 1 mm (dark yellow box) and 2 mm (orange box). **b** Model for the regulation of *C. aegerita* AAE-3 FB development. Indices I and II symbolize consecutive fruiting flushes that are typical for *C. aegerita* fructification. Green lines represent a presumed functional role in a certain stage by the respective putative *C. aegerita* AAE-3 orthologs of proteins with experimentally ascertained function from *S. commune* and *C. cinerea*. These presumptions are necessarily also based on the differential expression data of the corresponding *C. aegerita*-FRG from the present study. For the sake of simplicity this categorization also extends to the *C. aegerita* AAE-3 counterparts of the *PRI1* to *PRI4* genes of *C. aegerita* SM51 where functions were presumed based on transcription profiling and in silico analyses. Putative interaction between Dst1 and Bwc2 is symbolized by a thin line ending in a diamond on each side. Blue lines represent hypothetical roles within *C. aegerita* AAE-3 fruiting body development solely based on the expression data recorded within the present study. Dotted lines represent conjectured (in) direct transcriptional activation/repression of target genes based on publications on putative counterparts from another mushroom. Grayed-out genes did not show differential expression in the present study in the respective developmental stage(s) of *C. aegerita* AAE-3 but have previously been shown to trigger relevant fruiting body developmental processes in the model mushroom *S. commune* [10, 12]

Among the genes that are known to be crucial for the initiation of fruiting, Pcc1 from *C. cinerea* is supposed to be either a repressor or an interaction partner of the heterodimer of homeodomain proteins HD1 and HD2 that triggers mating locus *A*-regulated development including fruiting [20]. Accordingly, its putative *C. aegerita* ortholog *PCC1* is highly expressed already from the beginning of the *C. aegerita* fruiting process. *PCC1* shows a high expression already in uninduced young mycelium (> 2000 normalized reads), which permanently increases to > 6000 NRC after sporulation, and also exhibits high expression values in FB tissue samples of different fruiting stages (Fig. 7). Of the differentially expressed genes relevant to fruiting initiation in the related agaric *S. commune* [10, 12], the transcription factor-encoding genes *BRI1* and *FST4* [56] showed a clear transcriptional induction in primordia and, in the case of the latter gene, also in immature FBs of *C. aegerita* AAE-3. Induction of both genes in primordia is in agreement with the findings by Ohm et al. [10] and Pelkmans et al. [12]. They showed that the Δ*fst4* mutant is not able to form FB initials (‘aggregates’) as it triggers the transition from vegetative growth to fruiting. On the other hand, Pelkmans et al. [12] showed that the Δ*bri1* mutant is delayed in fruiting, chiefly due to a reduced growth speed that may be explained by downregulation of crucial cellular processes. Our observation that *BRI1* and *FST4* also get strongly induced during the sporulation/post-sporulation stage in the mycelium might relate to the phenomenon that *C. aegerita* fruits in consecutive flushes ([5], Fig. 7) once the fruiting process has been triggered by environmental cues. In the present study, the induction of genes like *PCC1*, *BRI1* and *FST4*, induced at early developmental stages in *S. commune* [10, 12, 34], at the sporulation/post-sporulation stage may be characteristic of species that fruit in consecutive flushes like *C. aegerita*. If not revealed by future analysis of mycelium close to elder first flush FBs of *S. commune* that another increase in expression of such FRGs may just happen much later there, the expression maximum of *C. aegerita PCC1*, *BRI1* and *FST4* at the (post-)sporulation stage might mark a big difference to species producing even more long-lasting FBs. In contrast to *C. aegerita* FBs, *S. commune* FBs are characterized by an extremely long persistence (> 50 years, [72]), releasing spores whenever conditions are favorable [7]. Alternatively, the here observed post-sporulation induction could also be a hint that transcription factors like Bri1 may not exclusively govern the expression of genes that are involved in the generation of FB structures. Possibly, transcriptional induction of genes involved in fruiting-associated processes could be regulated, too, by such a factor. Such could be volatile production for spore disperser attraction or fungivore repellency. As a gene triggering the formation of light-induced FB initials, which also seems to play a role in subsequent fruiting stages [27] (Fig. 7), *CFS1* showed an expression profile that peaked in fruiting-induced mycelium and markedly decreased in later mycelium stages.

Induction in fruiting-induced mycelium in contrast to vegetative mycelium correlates well with the expression data of the *C. cinerea* ortholog in mycelial stages [27]. Still, there may be a difference between this FRG’s expression in both fungi, since it shows an expression maximum in primordia of *C. cinerea* implying a role also for later stages of FB development in the plectenchyma of *C. cinerea* [27], while in *C aegerita* expression remains high in mycelium to a considerable amount until the premature FB stage but not in primordia of *C. aegerita*. This, of course neither rules out a putative indirect action of Cfs1 on *C. aegerita* FB stages forming on the fruiting-induced mycelium connected to them nor that a putative role of Cfs1 in later stages of development might potentially be mediated post-transcriptionally.

Being required for proper primordia development in response to illumination [26, 29, 30], the expression patterns of *BWC2* and *DST1* mostly match the expression patterns of their *C. cinerea* counterparts (Fig. 7). In addition, our quantitative PCR data are chiefly in agreement with the transcriptomic data on *BWC2* and *DST1* expression (see Fig. 3 and Additional file 3: Fig. S4), even though standard deviation in the sample from stipes of immature fruiting bodies was too high to confirm differential expression of *DST1* and *BWC2* there. Both genes are upregulated from the primordium stage onwards, interestingly also in the cap of developing FBs, implying a possible function in FB tissue generation there (Fig. 7). As a future experimental directive, it might be worth to test, e.g. by transcript profiling, whether a potential cap differentiation-associated tissue specificity of Dst1 expression might apply, which would corroborate the hypothesis that Dst1 may even have a function in the induction of cap formation. Such a cap tissue-specific expression localization, potentially from the primordial stage on, was also presumed for ageritin expression [72]. Moreover, transcriptomic data indicates upregulation of *DST1* in fruiting-induced mycelium, indicating a hypothetical role of *DST1* in the transition of FB initials to primordia that would need verification by functional analyses. Similar to *DST1*, *DST2* was also upregulated in primordia and developing FBs, as expected compared to its *C. cinerea* counterpart [29, 30] (Fig. 7).

According to Ohm et al. [10], the expression values of *FST3* in developing and maturating FBs in *C. aegerita* might restrict the extent of additional primordia formation ensuring that some FBs can fully develop assuming limited resources for sexual reproduction [10]. The paralogized *C2H2*, which is relevant to the transition from FB initials to primordia in the related agaric *S. commune* [10, 12], showed very low expression values. This leaves it open to future work how these paralogs might be involved in fruiting and whether they might be subject to post-transcriptional regulation. The same applies for *HOM2*, which also displayed less than 25 NRC (Fig. 7).

Among the cohort of genes associated with proper primordial development, the putative *C. cinerea* counterpart of *ICH1* had been characterized by a remarkable primordium malformation phenotype in the case of a recessive mutation of its DNA sequence [21]. Exhibiting an expression pattern that aligns very well with its *C. cinerea* counterpart, it can be presumed that *ICH1* should be similarly essential to proper primodium development (Fig. 7). In another *C. aegerita* wild type strain [19, 22–24], four genes were reported as transcriptionally induced during primordium development. In the genome sequence of *C. aegerita* AAE-3 [56], paralogization (commonly also referred to as gene duplication(s)/gene multiplication) for three of them can be detected. In the case of *PRI1*, the difference between the expressions of the paralogs during fruiting is similarly striking as it has been recently observed with the basidiome defense genes *AGT1* and *AGT2* of which only the former is transcriptionally induced during fruiting although both genes encode a functional ribotoxin [72, 73] and are located directly adjacent to each other on the chromosome. Both, *PRI1–1* and *PRI1–2* are supposed to encode a hemolysin. In the case of Pri1–1, a hemolytic activity has been proven at least for its putative *Pleurotus ostreatus* ortholog pleurotolysin [74]. Thus, the here-observed extraordinary high expression of *PRI1–1* during fruiting may go well together with a potential defense function of this protein to protect *C. aegerita* from predation during FB formation (Fig. 7). Supported by comparably high transcription levels especially in fruiting-induced mycelium and in primordia, and by the fact that *PRI2* should encode a hydrophobin, one may speculate whether Pri2 play an essential role for FB initial formation (Fig. 7). Making this point, Ohm et al. [10] discuss phenotypes and expression profiles, e.g. of the Δ*fst4* mutant which cannot form FB initials and displays a severely affected expression of dikaryon-specific hydrophobins. The scarcity of sequence motif annotation of hydrophobin genes [56] makes it very difficult to speculate about their possible functions, even for the highly expressed paralogs of *PRI3* (AAE3_14114 and AAE3_14115) and *PRI4* (AAE3_04665). Potential functions of *PRI3* and *PRI4* for the development of primordia into immature FBs or FB maturation-associated processes, as tentatively adumbrated by Fig. 7, may only be revealed to the point once gene knockout methodology is established for *C. aegerita*.

In *C. cinerea*, the gene encoding the Exp1 protein has been attributed a role in the basidiome maturation associated process of cap expansion [28]. *EXP1* has its expression maximum when cap expansion takes place in premature fruiting bodies implying a conserved function with its *C. cinerea* counterpart. Besides this, it is also upregulated in induced mycelium at the beginning of the fruiting process. Since a faint upregulation of the *C. cinerea* gene has been observed already in primordia [28], it may not be unexpected that the HMG-box transcription factor Exp1 could also regulate genes outside FB maturation (Fig. 7). Being involved in the FB maturation-associated process of stipe elongation in *C. cinerea*, the paralogs of *ELN3* exhibited diverging expression patterns during fruiting. Only *ELN3–1* (AAE3_00364) was exclusively upregulated in FB tissue. Displaying its maximum transcription in primordia, and to a lesser extent in maturing FBs, its function may extend also to a role in primordial plectenchyma formation. In contrast, *ELN3–2* (AAE3_06792) and ELN3*–3* (AAE3_13318) had their expression maxima in uninduced mycelium with lesser expression maxima in developing FBs or primordia (only *ELN3–2*). Compared to other model agarics, as anticipated by Gupta et al. [56], the here-recorded differential gene expression of *EXP1* and the *ELN3* paralogs implies a more complex genetic regulation of basidiome maturation in *C. aegerita* (Fig. 7). This, of course, needs verification by functional genetics analyses in future studies.

In *S. commune*, the Gat1- and the Hom1-encoding gene get transcriptionally induced mainly during development of FBs although a slight expression is detectable for *HOM1* already during aggregate and primordia formation [10, 12]. Functional analyses revealed both transcription factors to be important for plectenchyma formation in developing FBs of this species [10, 12]. Also, despite a possible (partial) shift in function during fruiting, both transcription factors are conserved also among other Agaricales members [34]. Differential expression of *HOM1* and *GAT1* in *C. aegerita* is chiefly congruent with the expression pattern of their *S. commune* counterparts [12]. This provides evidence to hypothesize that their functions should be conserved in *C. aegerita* (Fig. 7).

The cluster analysis on FRGs expressed in FB tissue samples (Additional file 3: Figure S2) resulted in clear groups of strongly positively correlated genes. This cluster formation is also chiefly in agreement with the assignment of genes into the expression maxima cohorts established within Fig. 3, particularly for genes that have early or late expression maxima in FB tissue. In contrast, the cluster analysis of the mycelium samples showed much less comprehensive cluster formation. Accordingly, the general overlap between the clusters from the cluster analysis and the gene cohorts revealing early or late expression maxima in mycelial stages (Fig. 3, Additional file 3: Figure S3) was also much less comprehensive. This underlines that a cluster analysis can be very useful to time-efficiently identify strongly positive correlated genes with common differential expression patterns.

### VOC related biosynthesis pathways

Sesquiterpene synthases from fungi have proven to often have a high catalytic promiscuity, leading to a highly diverse number of sesquiterpenes despite low variety of enzymes [75–78]. Furthermore, modifications of terpenes catalyzed by cytochrome P450 monooxygenases, oxidoreductases and different group transferases [79] might also contribute to the high diversity of sesquiterpenes observed in the HS of *C. aegerita* [54]. Most sesquiterpenes produced by the recombinant *E. coli* clones were also present during sporulation in the HS of *C. aegerita* [59]. Interestingly, genes of the mevalonate pathway as well as of the STSs showed generally higher expression levels in the mycelium than in FB samples during the late phase of the fruiting process. This indicates that the mycelium rather than the FB tissue is the origin of the high amounts of sesquiterpenes observed in the HS of *C. aegerita* during sporulation [54]. This would also explain the occurrence of sesquiterpenes in the HS of the monokaryon AAE-3-40, which do not develop FBs [54], and why these substances were not detected in previous studies on VOCs in FBs of *C. aegerita* [80–82]. In this context the question remains, if sporulation triggers the release of sesquiterpenes or if sesquiterpenes are somehow associated with the release of spores. Nonetheless, it seems that sesquiterpenes are involved in a so far barely understood communication between mycelium and FBs.

In addition to the sesquiterpenes, the biosynthesis of oxylipins in fungi is of special interest. The pathways leading to volatile fungal oxylipins, including the typical mushroom C8 VOCs such as oct-1-en-3-ol, octan-3-one and octan-3-ol, but also other oxylipins, like 2-pentylfuran, are still scarcely known despite their ubiquitous occurrence in fungi. Fungal volatile oxylipins are derived from linoleic acid and are therefore connected to the biosynthesis of fatty acids [60]. Generally, we observed a higher expression of genes involved in the fatty acid synthesis and further processing to linoleic acid in FB stages than in mycelial stages. Comparable data was obtained by Wang et al. investigating one mycelium and one FB developmental stage of *C. aegerita* and showing an upregulation of genes involved in fatty acid metabolism in FBs [66]. A similar upregulation was observed in *S. commune* during FB development [13]. In fact, a recently published comprehensive transcriptomic study dealing with six different Agaricomycetes species and the gene expression during various developmental stages revealed as well a higher expression of genes involved in lipid biosynthesis in fruiting stages throughout all investigated species [33]. This might indicate that this pattern is quite common among mushroom-forming fungi.

**Fig. 8:**
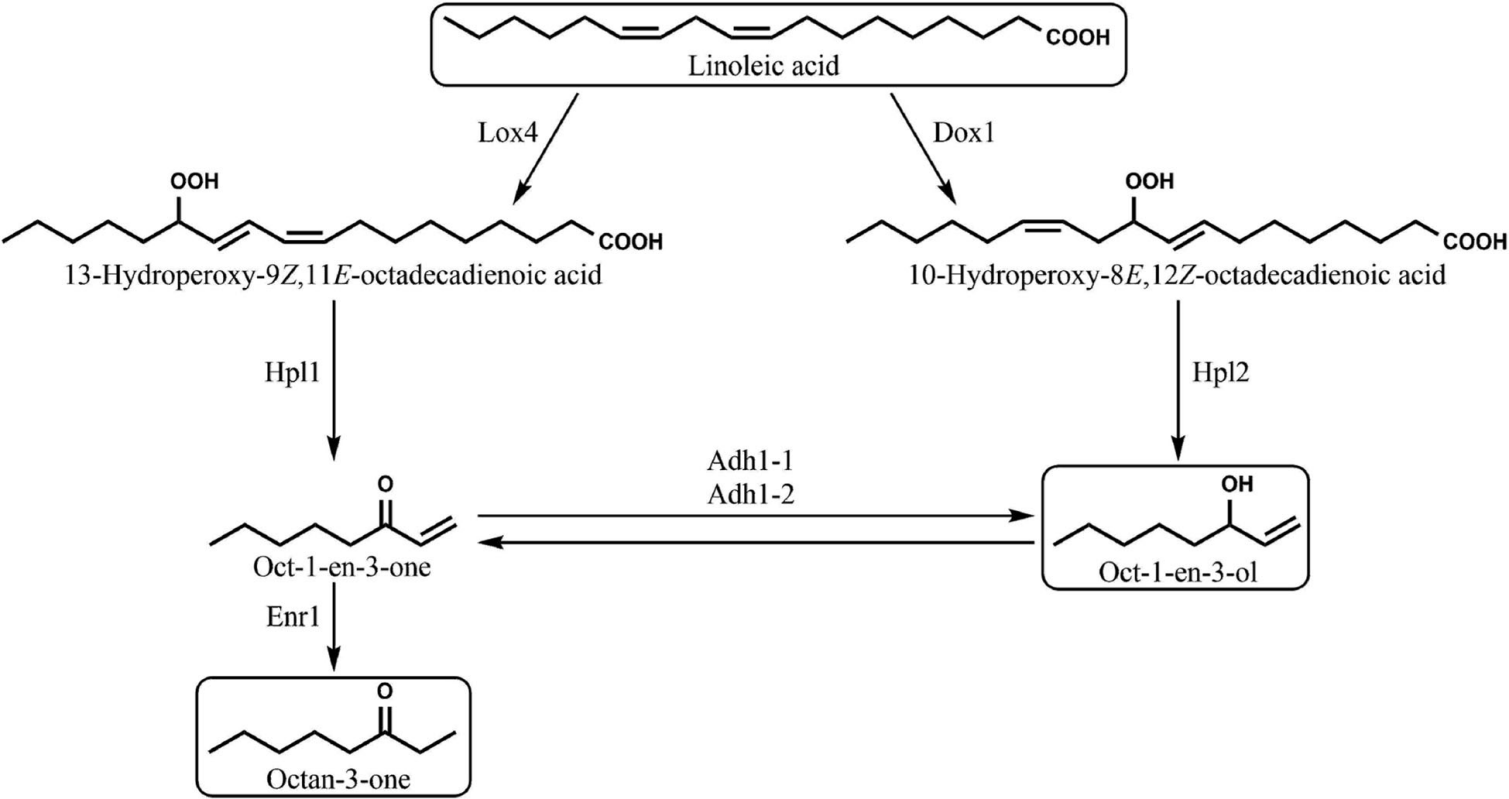
Putative pathways for the enzymatic formation of oxylipins derived from linoleic acid in *C. aegerita*. Lox4 (AAE3_04864), Dox1 (AAE3_13098), Hpl1 (AAE3_05330), Hpl2 (AAE3_09203), Adh1–1 (AAE3_00054), Adh1–2 (AAE3_06559), Enr1 (AAE3_13549)

It is widely accepted that LOXs and DOXs are involved in fungal oxylipin biosynthesis using linoleic acid as precursor, although little is known about the exact formation processes [60]. Despite the prominent role of LOXs in the fungal oxylipin synthesis, only three LOXs in Basidiomycota are functionally characterized so far [83–85]. Among them, the *C. aegerita* Lox4, a 13-LOX exclusively producing 13-hydroperoxy-9,11-octadecadienoic acid (13-HPOD) [85] whereas LOXs from *Pleurotus ostreatus* [83] and *Pleurotus sapidus* [84] produce, along with the main product 13-HPOD, also minor amounts of 9-hydroperoxy-10,12-octadecadienoic acid (9-HPOD). The role of 13-HPOD in the formation processes of fungal VOCs is still largely unknown. It seems that the biosynthesis of n-hexanal is associated with 13-HPOD [86]. However, it was also proposed that 13-HPOD is involved in the synthesis of oct-1-en-3-one, which is subsequently reduced to oct-1-en-3-ol or octan-3-one by so far unknown ADHs or ene-reductases, respectively (Fig. 8). In parallel, it is assumed that oct-1-en-3-ol emerges from 10-hydroperox-8,12-octadecadienoic acid (10-HPOD) as precursor [49, 87]. Nonetheless, several studies excluded 13-HPOD from being a precursor of oct-1-en-3-ol [63, 86, 88]. Recently, Tasaki et al. determined the transcription levels of *PoLOX1* and *PoLOX2* along with the oct-1-en-3-ol content and LOX activity in mycelium, primordia, young FBs and mature FBs of *P. ostreatus* [49]. In agreement with our results, LOX genes were mostly expressed in FB developmental stages with *PoLOX1* mainly in primordia and *PoLOX2* primarily in fully developed FBs. Tasaki et al. reported a correlation between LOX activity and *PoLOX1* expression in FB developmental stages. However, no LOX activity was detected in the mycelium, excluding LOXs as a source for oct-1-en-3-ol present in the mycelium [49]. Our transcriptomic data indicates similar with only little pronounced expression of LOX genes in the mycelium despite the presence of oct-1-en-3-ol during all mycelial stages [54].

Thus, a DOX might be responsible for the oct-1-en-ol production. In contrast to the quite well understood DOXs from Ascomycota [89], to our knowledge, no DOXs from Basidiomycota are characterized so far. Recently, Oliw analyzed reaction products of *Rhizoctonia solani* mycelium after addition of linoleic acid and observed substances probably derived from 9*S*-DOX-AOS (allene oxide synthase), 8*S*-DOX-8,9-ODS (oleate diol synthase) and 8*R*-DOX activity [90]. The biosynthesis of 8*S*-HPOME, 8*S*,9*S*-DiHOME and 8*R*-HPODE were linked to the proteins KEP54849 or KEP46854. This is due to the presence of a NXXQ motif in the I-helix of the CYP (cytochrome P450) domains, proven to be involved in the hydroperoxide isomerase activities of 7,8- and 5,8-LDS (linoleate diol synthase) [91], and the occurrence of the YRWH sequence. Whereas KEP52552, lacking the NXXQ motif and revealing an uncommon YHWH sequence, was connected to the 9*S*-DOX-AOS activity [90]. On basis of amino acid sequences of characterized DOXs from ascomycetes and putative DOXs of the basidiomycete fungus *R. solani*, two putative DOXs of *C. aegerita* show highest similarities with the ones from *R. solani* (AAE3_00407 with KEP52552 and AAE_13098 with KEP54849) (Additional file 7: Figure S12), which might indicate similar reaction products of these DOXs. Nonetheless, the putative DOXs in *C. aegerita* reveal remarkable differences to the two putative DOXs KEP52552 and KEP54849 from *R. solani* regarding leucine and valine residues in the DOX domain (AAE3_13098: Val-403, Leu-407; AAE3_00407: Phe-331, Leu-335) (Additional file 7: Figure S13A). These are confirmed to be crucial for the oxygenation at C^− 10^ and C^− 8^ of linoleic acid [92, 93]. In this context, it is worth mentioning that the putative DOX KEP46854, which shows overall less amino acid sequence similarity with the two putative DOXs from *C. aegerita* (Additional file 7: Figure S12), possesses a VXXXL residue. The same applies to AAE3_13098. Additionally, both putative DOXs from *C. aegerita* harbor, unlike KEP52552, the YRWH motif, commonly found in the DOX domains of 8- and 10-DOXs, whereas the corresponding sequence in 9*S-*DOX-AOS, 9*R-*DOX-AOS and 9*R*-DOX enzymes is normally YRFH (Additional file 7: Figure S13A). In the C-terminal CYP domains of AAE3_13098 and AAE3_00407, the NXXQ motif is absent. This motif is commonly found in 10*R*-DOX-EAS (epoxy alcohol synthase), 5,8- and 7,8-LDS, but not in 9*S-*DOX-AOS, 9*R-*DOX-AOS, 10*R*-DOX-CYP and 9*R*-DOX enzymes, the latter lacking a CYP domain and consequently also this motif [94] (Additional file 7: Figure S13B). Furthermore, like 10*R*-DOX-CYPs, both putative DOXs are missing a conserved cysteine residue in the CYP domain which serves as the fifth iron ligand in P450 enzymes and is essential for the function [93] (Additional file 7: Figure S13B). Therefore, it is likely that the CYP domains of DOXs from *C. aegerita* are, comparable to 10*R*-DOX-CYPs, not functional [93]. Hence, the putative DOXs from *C. aegerita* and 10*R*-DOX-CYPs have some structural features in common, even though the important leucine and valine residues mentioned above as part of a conserved LRTIV motif in 10*R*-DOX-CYPs differ from residues observed in AAE3_13098 and AAE3_00407 (Additional file 7: Figure S13A). Interestingly, 10*R*-DOX-CYPs of the fungal phylum of Ascomycota reveal the potential to form volatile C8 compounds since they produce inter alia 10-HPOD, which serves as a precursor for oct-1-en-ol [55, 63]. Moreover, addition of linoleic acid to an extract of *E. coli* containing a recombinant 10*R*-DOX-CYP from *A. nidulans* resulted in the production of oct-1-en-3-ol, oct-2-en-1-ol, oct-2-enal and octan-3-one [93]. Taking these aspects into account, it is possible that DOXs from *C. aegerita* might constitute a novel DOX subfamily with so far unknown products. In this context, the putative DOX AAE3_13098, which also shows comparably high transcription in the mycelium and similarities of its expression course to the oct-1-en-3-ol pattern in the HS of *C. aegerita*, seems to be an interesting candidate for future characterization studies. This way, one may become able to tap the so far neglected topic of DOXs in Basidiomycota and their potential role in (volatile) oxylipin formation (Fig. 8).

In plants, the cleavage of fatty acid hydroperoxides by HPLs is well known and HPLs can be divided into 9-HPLs, 13-HPLs and 9/13-HPLs responsible for the synthesis of C6- and C9-aldehydes which have, along with their derivatives, various functions in plants [95, 96]. In contrast, there is only scarce information about fungal HPLs. In an early study, Wurzenberger and Grosch incubated 9-, 10-, 12- and 13-HPOD with a protein fraction isolated from an extract obtained from the button mushroom *Agaricus bisporus* [63]. They observed that only addition of 10-HPOD resulted in the formation of oct-1-en-3-ol and 10-oxo-*trans*-8-decenoic acid probably due to the presence of a 10-HPOD specific HPL. Despite the fact that other studies suggest the existence of such an enzyme as well [86, 88], to our knowledge no fungal HPL has been isolated and characterized so far. Phylogenetic analysis revealed that putative HPLs encoded by AAE3_09218, AAE3_09203, AAE3_06699 and AAE3_11433 were closer related to characterized plant HPLs and AOS than the other putative HPLs we found in the genome of *C. aegerita* (Additional file 7: Figure S14). In FB samples as well as in the mycelial stages, the course of expression of the putative HPL gene AAE3_09203 was comparable to the transcription of the putative DOX gene AAE3_13098. Both genes showed similarities to the oct-1-en-3-ol pattern in the HS of *C. aegerita* (Additional file 7: Figure S15), making AAE3_09203 an interesting candidate for characterization studies. It is worth mentioning that AAE3_05330, identified as a putative HPL by means of BLAST search using a 13-HPL protein sequence of *A. thaliana*, shared similarities in the expression pattern with the gene coding for the characterized Lox4. Both genes were highly transcribed in late fruiting stages along with the appearance of octan-3-one, which might indicate an involvement of both enzymes in the formation of this C8 VOC (Fig. 8, Additional file 7: Figure S16).

In addition to the oxygenation of linoleic acid by LOXs/DOXs and the subsequent cleavage into C8 compounds, further enzymes are necessary to provide a plentitude of C8 VOCs in fungi. It has been demonstrated that in *A. bisporus* oct-1-en-3-one can be converted to oct-1-en-3-ol as well as to octan-3-one, probably by means of two different enzymes [64]. These results were confirmed by Wanner and Tressl using a crude enzyme extract of *Saccharomyces cerevisiae* [65]. Furthermore, they were able to isolate two reductases capable to convert oct-1-en-3-one to octan-3-one [65]. In this context, ADHs and ene-reductases might play an important role, explaining the observed decrease of oct-1-en-3-ol during sporulation and, thereafter, the increase of octan-3-one in the HS of *C. aegerita* [54]. A heterologously expressed ADH from the fungus *Neurospora crassa* was able to oxidize octan-1-ol [97]. Using the amino acid sequence of this ADH (Q9P6C8), we identified putative ADHs in the genome of *C. aegerita* by means of BLAST search which might be able to oxidize oct-1-en-3-ol to oct-1-en-3-one (Fig. 8, Additional file 6: Figure S10). Of those genes, the putative ADH gene AAE3_00054 showed highest expression of all putative ADH genes in FB development stages. This high expression came along with a decreasing amount of oct-1-en-3-ol and an increasing amount of octan-3-one in the HS of *C. aegerita*. In addition, phylogenetic analysis showed that AAE3_00054 and AAE3_06559 are closer related to the ADH of *N. crassa* than the other putative ADHs (Additional file 7: Figure S17).

The gene AAE3_13549 coding for a putative ene-reductase showed the highest transcription levels of all putative ene-reductases in late stages of FB development. Interestingly, amino acid sequence alignment revealed notable similarity (54%) between the putative *C. aegerita* ene-reductase encoded by AAE_13549 and the characterized plant ene-reductase from *N. tabacum* (Q9SLN8) known to be able to reduce oct-1-en-3-one (Fig. 8, Additional file 7: Figure S18). Additionally, in FB development stages, resemblance between the transcription of AAE3_13549 and the putative ADHs AAE3_00054, which codes for a putative ADH, along with the decreasing amount of oct-1-en-3-ol and the increasing amount of octan-3-one (Additional file 7: Figure S19) supports the proposed transformation of oct-1-en-3-ol via oct-1-en-3-one to octan-3-one [49, 64].

Overall, it seems that the first occurrence of octan-3-one in the HS of *C. aegerita* in late FB developmental stages is probably due to enzymatic activities in the FB tissue and not in the mycelium. This assumption bases on the fact that the transcription levels of genes coding for enzymes putatively related to the formation processes of C8 VOCs, such as enzymes involved in the fatty acid synthesis, LOXs, putative DOXs, putative HPLs, putative ADHs and putative ene-reductases, in FB stages showed transcription patterns that were matchable to the octan-3-one production. This would also explain why octan-3-one was not observed in the HS of *C. aegerita* monokaryotic strains unable to develop FBs [54]. In contrast, the transcriptome data suggest that the origin of the sesquiterpenes appearing in the HS of *C. aegerita* during the sporulation is the mycelium instead of the FBs. This is highly interesting as the sesquiterpene production occurs during sexual sporulation. The formation of various VOCs in different morphological parts of *C. aegerita* might be, along with the changing volatilome during different developmental stages [54], an important part in fungal communication which involves several VOCs as infochemicals with numerous functions (reviewed in [50, 51]). This is an aspect, which should be kept in mind during further studies dealing with e.g. fungal intra- and interspecific VOC based communication. In total, the combination of volatilome and transcriptome data proved to be a powerful tool to elucidate coherences regarding the VOC biosynthesis pathways in fungi.

## Conclusions

In this work, we investigated the changes in the transcriptome of *C. aegerita* during different points in time of FB development including seven mycelial and five plectenchymatic samples. On the one hand, the transcriptomic data generated here gave first insights into how the network of known FRGs may direct the complex process of FB development in *C. aegerita*. The here-observed differential expression patterns of partially highly paralogized FRGs during fruiting in contrast to the situation in the other model agarics *C. cinerea* or *S. commune* suggests a seemingly more complex regulation of fruiting in *C. aegerita*. On the other hand, by comparing the transcriptome with volatilome data of a recently conducted study [54], we were able to identify enzymes potentially involved in the biosynthesis of C8 oxylipins. Despite ubiquitously found in fungi and contributing to the typical mushroom odor, little is known about pathways leading to C8 based VOCs. To further elucidate this topic, enzymes of interest identified in this study, including LOXs, DOXs, HPLs, ADHs and ene-reductases, are valuable candidates for further studies. Additionally, we were able to localize the mycelium as the presumable main source of observed sesquiterpenes, whereas the in late stages detected changes in the C8 compound profile is most likely due to the activity of enzymes located in the FB tissue.

## Methods

### Fungal materials

The tested dikaryotic strain *C. aegerita* AAE-3 was grown at 24 °C in the dark in crystallizing dishes (lower dish: 70 mm in diameter, upper dish: 80 mm in diameter) with 16 mL 1.5% MEA (containing 15 g malt extract and 15 g agar per L) and sealed with Parafilm™. Ten days after the inoculation, the Parafilm™ was removed and the samples were transferred to a climate chamber (24 °C, 95%rH, 12/12 h day/night rhythm) and cultured on glass plates for further 18 days. Seven developmental stages of *C. aegerita* AAE-3 were tested, consisting of young mycelium (day 10 post inculation, p.i.), mycelium (day 14 p.i.), primordia (day 18 p.i.), immature FBs (day 20 p.i.), premature FBs (day 22 p.i.), sporulation (day 24 p.i.) and post sporulation (day 28 p.i.). Accordingly, seven mycelium and five FB stages were sampled. FB stages were collected by means of a scalpel used to carefully separate the FB samples from the mycelium. From a single agar plate only FB samples of a certain stage were sampled using the whole FB for RNA extraction and discarding younger stages (for details see Fig. 1). Mycelium samples were obtained using a spatula to gently remove the mycelium from the agar plate and thereby avoiding to collect possible FB stages. All samples were stored in RNAlater (Quiagen, Venlo, Netherlands) at − 20 °C. Each stage was grown in six replications of which two comparable samples were pooled prior to RNA extraction resulting in 36 RNA samples for sequencing. Accordingly, transcriptomic data presented are the mean values of RNA sample triplicates.

### RNA isolation and sequencing

For the RNA extraction, RNAlater was removed and samples were frozen in liquid nitrogen and ground into powder using mortar and pestle. Total RNA was extracted using TRIzol® (Life Technologies, Carlsberg, California, USA) according to the manufacturer’s instructions. Obtained RNA was solved in DEPC treated water and quantity as well as quality was assessed by means of photometric analysis (Pearl nanophotometer, Implen, Munich, Germany) and agarose gel electrophoresis (Peqlab electrophoresis chamber, VWR Life Science, Radnor, Pennsylvania, USA). RNA samples were stored at − 80 °C. For sequencing, RNA samples were send on dry ice to Lexogen (Lexogen GmbH, Vienna, Austria). The quality of the RNA samples was verified by Lexogen using a capillary gel electrophoresis system (Bioanalyzer, Agilent, Waldbronn, Germany). The complete sequencing procedure was offered as a Lexogen QuantSeq FWD SR5 service, including RNA quality control, RNA quantification, QuantSeq FWD library preparation for Illumina sequencing, NextSeq 75cyc high output sequencing, read trimming, mapping and quantification. Cutadapt version 1.16 [98] was used to trim the reads by removing trailing poly(A) and poly(G) as well as adapter sequences. STAR aligner version 2.5.3a (for details see: https://github.com/alexdobin/STAR/blob/master/doc/STARmanual.pdf) was used to align the trimmed reads on the *C. aegerita* reference genome [56] (version 2.2 of the genome has been used and can be downloaded via the respective genome browser (http://www.thines-lab.senckenberg.de/agrocybe_genome/). Quantification of the aligned reads was performed by featureCounts version 1.6.2. The QuantSeq 3′ mRNA sequencing method generates for each transcript only one fragment so the number of reads can be linked directly to the number of transcripts and is therefore proportional to the gene expression [99]. The average number of reads over all samples used for the alignment was 14.4 million reads per sample of which 93.1% resulted in a unique alignment to the reference genome and 1.4% were mapped to multiple loci. About 0.1% of the reads were discarded since the mapping resulted in too many loci and around 5.4% of the reads were too short for an adequate alignment.

### Transcriptome analysis and bioinformatics

Transcriptome data analysis were performed and implemented in R (version 3.6.0) [100]. Different R packages were applied as parts of scripts used for the transcriptome analysis. DEG analysis was accomplished by means of the R package “ImpulseDE2” (version 1.8.0) displaying not only permanent but also transient changes at the level of transcription [101, 102]. Accordingly, DEGs can be classified into four groups: transition up for monotonous upregulated genes, transition down for monotonous downregulated genes, transient up for transiently upregulated genes and transient down for transiently downregulated genes (for details see Fisher et al. [101]). The “ggplot2” R package (version 3.1.1) is part of the “tidyverse” collection and a powerful and versatile tool for graphical visualization [103]. This package was applied to generate the PCA plots. The subsequent used Friedman test and the Wilcoxon-Nemenyi-McDonald-Thompson test were originally implemented by Galili [104] and internally based on the R packages “coin” and “multcomp”. The R package “ComplexHeatmap” (version 2.0.0) were used to visualize the correlation matrices. For the correlation analysis Spearman rank correlation was applied. All R scripts used within this publication are deposited at https://github.com/AnnsophieWeber/ComparisonOfMetabolomeAndTranscriptomeData.

### Identification of proteins in *C. aegerita*

Generally, proteins were identified in the genome of *C. aegerita* using BLAST search (Geneious version 11.1.5, Biomatters, New Zealand) using amino acid sequences of mainly characterized proteins against the UniProt database [105]. Generally, a blastp E-value threshold of 1e-10 was applied and hits with the lowest E-values and highest identity were blasted (blastp) against the UniProt database to verify the results. Multiple hits were compared by alignment of the protein sequences and a phylogenetic analysis. Phylogenetic analyzes were performed by means of Phylogeny.fr (http://www.phylogeny.fr/) using default parameters [106]. Alignments were carried out by using Clustal Omega (https://www.ebi.ac.uk/Tools/msa/clustalo/) with default parameters [107].

Protein IDs for FRGs in *C. aegerita* were obtained from Gupta et al. [56] with exception of a second putative homolog of *AaPRI1* from *C. aegerita* SM51 within the *C. aegerita* AAE-3 genome sequence represented by the ID AAE3_04306. For LOXs in *C. aegerita*, protein IDs were used which were published by Karrer and Rühl [85]. STS protein IDs were obtained from Zhang et al. [59]. For identification of enzymes involved in the mevalonate pathway analogues in *S. cerevisiae* were used: acetoacetyl-CoA synthase (P41338), 3-hydroxy-3-methylglutaryl-CoA synthase (P54839), 3-hydroxy-3-methylglutaryl-CoA reductase (P12683, P12684), phosphomevalonate kinase (P24521), diphosphomevalonate decarboxylase (P32377), isopentenyl-diphosphate delta isomerase (P15496), dimethylallyltransferase/farnesyl pyrophosphate synthase (P08524). For enzymes putatively involved in the fatty acid synthesis, analogs from different fungi were used: acetyl-CoA carboxylase (*Laccaria bicolor*, B0CUD8), fatty acid synthase (*Omphalotus olearius*, B3GN11), β-ketoacyl-CoA synthase (*S. cerevisiae*, P25358), Δ^9^-fatty acid desaturase (*L. edodes*, Q76C19), Δ^12^-fatty acid desaturase (Q65YX3, *L. edodes*). Putative DOXs were identified in the genome of *C. aegerita* using protein sequences of characterized DOXs from Ascomycetes including a 8R-DOX-7,8-LDS (*Gaeumannomyces graminis*, AAD49559), a 9R-DOX (*Fusarium oxysporum*, EGU79548) and a 10R-DOX-CYP (*Aspergillus fumigatus*, ABV21633). Putative HPLs of *C. aegerita* were identified by means of BLAST search using protein sequences of characterized members of the CYP74 family in plants including a 13-HPL (*Arabidopsis thaliana*, Q9ZSY9), a 9-HPL (*Prunus dulcis*, Q7XB42), a 9/13-HPL (*Cucumis sativus,* Q9M5J2) and a 13-AOS (*A. thaliana*, Q96242). Considering the features of known CYP74 proteins in plants, we chose for each of the four proteins mentioned above the top 10 matches revealing sequence lengths between 300 and 700 amino acids. Additionally, to reduce the number of putative HPLs to the essentials, only genes were considered showing maximum transcription levels higher than 300 NRC. Putative ADHs were identified using an ADH of the fungus *Neurospora crassa* (Q9P6C8) proven to be able to oxidize octan-1-ol [97]. To reduce the number of putative ADHs to the essentials, only genes were considered showing maximum transcription levels higher than 300 NRC. Putative ene-reductases were identified in the genome of *C. aegerita* using sequences of characterized non-FMN ene-reductases of plants accepting inter alia non-2-enal and oct-1-en-3-one as substrates [108, 109] (*Nicotiana tabacum*, Q9SLN8; *A. thaliana*, Q39172) and a fungal non-FMN ene-reductase (*Sporidiobolus salmonicolor*, A0A0D6ERK8). In addition, sequences of fungal FMN depending OYE ene-reductases were used proven to be able to reduce amongst others citral (geranial) which shows some structural similarities with non-2-enal and oct-1-en-3-one [110, 111] (*Pichia stipites*, A3LT82; *Meyerozyma guilliermondii*, A5DR62). To reduce the number of putative ene-reductases to the essentials, only genes were considered showing maximum transcription levels higher than 300 NRC.

### RT-qPCR based confirmation of expression values of selected candidate genes

To exemplarily validate our transcriptomic data on FB development of *C. aegerita* AAE-3 via RT-qPCR, an optimal combination of two reference genes (gene IDs AAE3_02268 and AAE3_07769) with high expression stability during vegetative growth and fruiting of *C. aegerita* was identified recently by Hennicke et al. and Tayyrov et al. [72, 73]. Primers for the reference genes and general RT-qPCR conditions are identical to the ones employed by Hennicke et al. and Tayyrov et al. [72, 73], while primers for the genes *HOM1*, *GAT1*, *BWC2* and *DST1* (Additional file 3: Table S4) were designed here, applying the same criteria. Mycelial and fruiting stage samples were obtained, extracted and RNA quality assessed as performed by Hennicke et al. and Tayyrov et al. [72, 73], from developmental stages/plectenchymatic samples of *C. aegerita* AAE-3 chiefly congruent to the ones of the RNA-seq analysis in the present study, deviating only by these: fruiting body initials (FBi) at day 15 to 16 post inoculation (p.i.); fruiting body primordia (P) at day 17 to the morning of day 19 p.i.; immature FBs separately sampled into stipe (iFBs) and cap (iFBc) at day 19 to the morning of day 21 p.i. Samples of the stages premature FBs (day 22 p.i.), sporulation (day 24 p.i.), and post sporulation (day 28 p.i.) were not assessed here.

## Supplementary Information

**Additional file 1: **Differential gene expression during fruiting body development. **Figure S1.** Heatmap of DEGs in mycelium and FB samples.

**Additional file 2: Table S1.** Read counts of all sequenced genes. **Table S2.** Genes showing a > 5 fold decrease between day 22 and day 22 in the mycelium. **Table S3.** Genes showing a > 5 fold increase between day 22 and day 22 in the mycelium.

**Additional file 3: **Transcription of fruiting-related genes (FRGs). **Figure S2.** Correlation of the expression of putative *C. aegerita* homologs of FRGs in plectenchymatic samples (FB ‘tissue’) during the fructification process. **Figure S3.** Correlation of the expression of putative *C. aegerita* homologs of FRGs in mycelium samples. **Figure S4.** RT-qPCR-based expression level assessment with *C. aegerita* orthologs of four well-known fruiting-related genes (FRGs) during fruiting of *C. aegerita*. **Table S4:** RT-qPCR primers for the FRGs *HOM1*, *GAT1*, *BWC2 *and *DST1*.

**Additional file 4: **Terpenoid biosynthesis. **Figure S5.** Expression of genes involved in the mevalonate pathway and of the sesquiterpene synthases Agr1 to Agr9 in *C. aegerita*. **Figure S6.** Transcription levels of the sesquiterpene synthases Agr1, Agr4, Agr8 and Agr9.

**Additional file 5: **The fatty acid metabolism. **Figure S7.** Expression of genes putatively involved in the fatty acid biosynthesis of *C. aegerita*.

**Additional file 6: Figure S8.** Proposed pathways for the enzymatic formation of fungal oxylipins. **Figure S9.** Transcription levels of putative HPLs as well as the relative concentrations of volatile oxylipins in the headspace of *C. aegerita*. **Figure S10.** Transcription levels of putative ADHs as well as the relative concentrations of volatile oxylipins in the headspace of *C. aegerita*. **Figure S11.** Transcription levels of putative ene-reductases as well as the relative concentrations of volatile oxylipins in the headspace of *C. aegerita*.

**Additional file 7: **Revealing putative enzymes of the oxylipin pathway in *C. aegerita*. **Figure S12.** Phylogenetic analysis of different DOXs. **Figure S13.** Partial amino acid sequence alignment of different DOXs. **Figure S14.** Phylogenetic analysis of different CYP74 proteins. **Figure S15.** Transcription levels of the putative DOX AAE3_13098 and the putative HPL AAE3_09203. **Figure S16.** Transcription levels of AAE3_04864 (*LOX4*) and the putative HPL AAE3_05330. **Figure S17.** Phylogenetic analysis of putative ADHs. **Figure S18.** Amino acid sequence alignment of ene-reductases. **Figure S19.** Transcription levels of the putative ADHs AAE3_00054 and AAE3_06559 as well as the putative ene-reductase AAE3_13549.

## Data Availability

Data are available within the NCBI BioProject PRJNA677924 under the BioSample numbers 16789160 to 16789171.

## Abbreviations

ADHs: Alcohol dehydrogenases
AOS: Allene-oxide synthase
CYP: Cytochrome P450
DEGs: Differentially expressed genes
DOXs: Dioxygenases
EAS: Epoxy alcohol synthase
FBs: Fruiting bodies
FRGs: Fruiting-related genes
HPLs: Hydroperoxide lyases
9-HPOD: 9-Hydroperoxy-10,12-octadecadienoic acid
10-HPOD: 10-Hydroperoxy-8,12-octadecadienoic acid
13-HPOD: 13-Hydroperoxy-9,11-octadecadienoic acid
HS: Headspace
LDS: Linoleate diol synthase
LOXs: Lipoxygenases
NRC: Normalized read counts
ODS: Oleate diol synthase
OYE: Old yellow enzyme
PCA: Principal component analysis
p.i: Post inoculation
RT-qPCR: Real-time reverse transcription quantitative polymerase chain reaction
STSs: Sesquiterpene synthases
VOCs: Volatile organic compounds
