# Supplementary material for: Transcriptome of different fruiting stages in the cultivated mushroom Cyclocybe aegerita suggests a complex regulation of fruiting and reveals enzymes putatively involved in fungal oxylipin biosynthesis
Source: BMC Genomics. 2021 May 4;22:324. doi: 10.1186/s12864-021-07648-5 (PMC8097960; doi:10.1186/s12864-021-07648-5)
Supplement: Supplementary file 1 — Additional file 1: Differential gene expression during fruiting body development. Figure S1. Heatmap of DEGs in mycelium and FB samples. [file 12864_2021_7648_MOESM1_ESM.docx]

**Differential gene expression during fruiting body development**

In the mycelium, as a result of the comparison of the different developmental stages, 129 genes were classified as transient down, 152 genes as transient up, 1,553 genes as transition down and 1,784 genes as transition up (Figure S1A), whereas for fruiting bodies, no genes were identified as transient down, 2 genes as transient up, 758 genes as transition down and 912 genes as transition up (Figure S1B). In total, both transcriptome data sets revealed 4,632 differentially expressed genes (DEGs) of which 2,960 genes were uniquely differentially expressed in the mycelium and 1,014 genes were only differentially expressed in fruiting bodies, leaving 658 DEGs both life stages had in common (Figure S1).


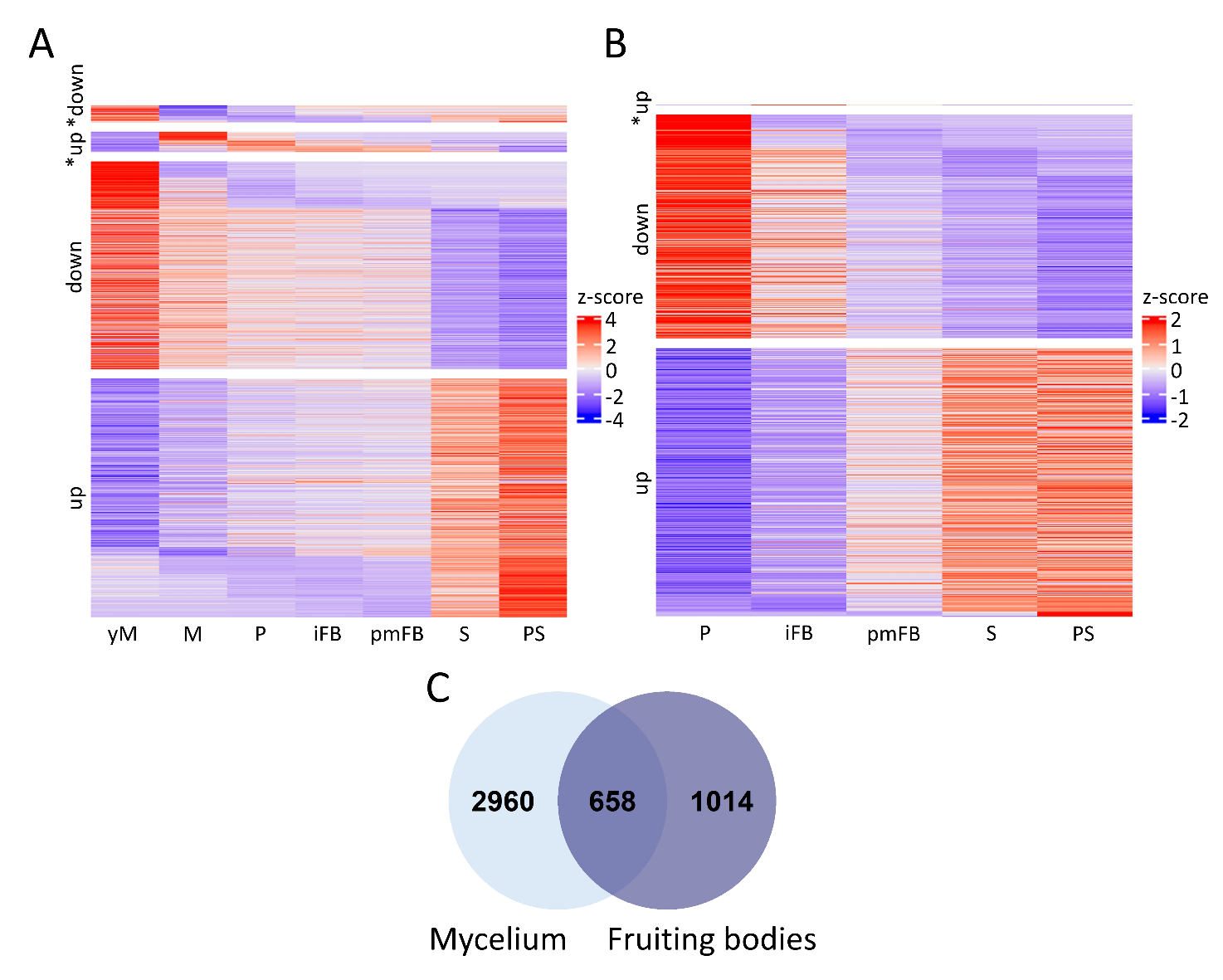


Figure S1: Differential gene expression during fruiting body development in C. aegerita. A) Differential expressed genes (DEGs) in the mycelium. B) DEGs in fruiting bodies. Each heatmap block represents one differential gene expression class:*down: transient down; *up: transient up; down: transition down; up: transition up. Normalized read counts were transformed to z-score values (respective scale to the right). Red colors indicate transcriptional upregulation while blue colors represent downregulation. yM: young (uninduced) mycelium (day 10 post inoculation, p.i.); M: mycelium (day 14 p.i.); P: primordia (day 18 p.i.); iFB: immature fruiting bodies (day 20 p.i.); pmFB: premature fruiting bodies (day 22 p.i.); S: sporulation (day 24 p.i.); PS: post sporulation (day 28 p.i.). C) Venn diagram presenting the overlap of DEGs among mycelium and fruiting bodies, as well as the number of DEGs only present in one sample type.
