# Supplementary material for: Transcriptome of different fruiting stages in the cultivated mushroom Cyclocybe aegerita suggests a complex regulation of fruiting and reveals enzymes putatively involved in fungal oxylipin biosynthesis
Source: BMC Genomics. 2021 May 4;22:324. doi: 10.1186/s12864-021-07648-5 (PMC8097960; doi:10.1186/s12864-021-07648-5)
Supplement: Supplementary file 3 — Additional file 3: Transcription of fruiting-related genes (FRGs). Figure S2. Correlation of the expression of putative C. aegerita homologs of FRGs in plectenchymatic samples (FB ‘tissue’) during the fructification process. Figure S3. Correlation of the expression of putative C. aegerita homologs of FRGs in mycelium samples. Figure S4. RT-qPCR-based expression level assessment with C. aegerita orthologs of four well-known fruiting-related genes (FRGs) during fruiting of C. aegerita. Table S4: RT-qPCR primers for the FRGs HOM1, GAT1, BWC2 and DST1. [file 12864_2021_7648_MOESM3_ESM.docx]

**Transcription of fruiting-related genes (FRGs)**

In FB samples, expression of all four *PRI4* paralogs, the *PRI3* paralogs *PRI3-1* and *-2*, *PRI2*, *BRI1*, *DST1* and *-2*, *ELN3-1* and *-2*, and *ICH1* strongly correlated with each other in a first cluster, the expression of *BWC2*, *PRI3-6* and *CFS1* strongly correlated with each other in a second one, and the expression of *EXP1*, *ELN3-3*, *PCC1* and *FST3* strongly correlated with each other in a third one (Figure S2). Among the *PRI3* paralogs, a strong correlation was also visible for the expression of *PRI3-1* and *-4*, whereas the expression of *PRI3-4* correlated with the one of *PRI3-6* in a fairly positive manner. Individual positive correlation was also detected between the expression of *FST4* versus *BWC2*, *DST1* and *-2*, *ELN3-1* and *-2*, *ICH1*, *PRI2*, *PRI3-1*, *-4* and *-6*, and *PRI4-2* to *-4*, as well as between the one of *HOM1* versus *FST3* and *CFS1* (Figure S2).

Some of these correlations were also observed in the mycelium samples where cluster formation was less comprehensive. Clusters of expression-wise strongly positive correlated genes were recognized, first, with all four *PRI4* paralogs, *DST1*, and *EXP1* (Figure S3; with *PRI4-2* and *DST1* revealing the weakest correlation among them) aligning with the correlation among *PRI4-1* to *-4* and *DST1* in the fruiting body samples (Figure S2). A second cluster was apparent between *PRI3-1*, *ICH1*, *CFS1*, *DST2* and *PRI2* (Figure S3) mirroring the correlation between *ICH1* and *DST2* observed in the fruiting body samples (Figure S2). A third cluster was recognized with *ELN3-3*, *FST3* and *BWC2* (Figure S3) comparable with the correlation between *ELN3-3* and *FST3* in the fruiting body samples (Figure S2). Two last clusters were evident in mycelium samples with *PRI3-4* and *-6*, *BRI1* and *PCC1* on the one hand, and with *ELN3-1* and *-2* on the other hand (Figure S3). There, *ELN3-1* and *-2* also showed a strongly positive correlation of their expression in plectenchyme samples (Figure S2). Furthermore, the positive expression-wise correlation between the *PRI3* paralogs *PRI3-1* and *-2* in plectenchyme samples aligned with the one in the mycelium samples, while the fairly positive correlation of the expression of *PRI3-4* and *-6* in the plectenchyme samples was complemented by a strongly positive correlation within the mycelium samples (Figure S3).


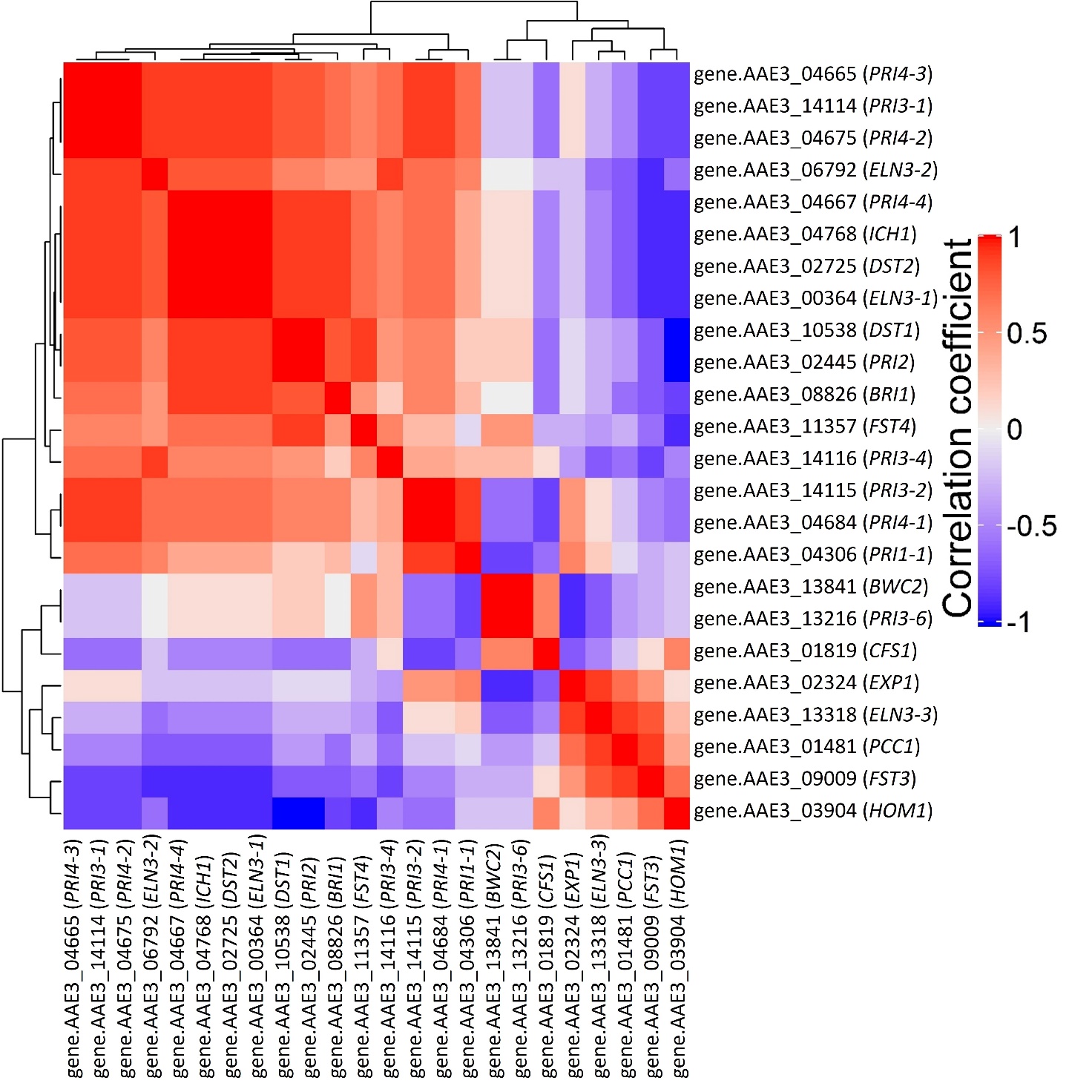


Figure S2: Correlation of the expression of putative C. aegerita homologs of fruiting-related genes (FRGs) in plectenchymatic samples (fruiting body ‘tissue’) during the fructification process. Only genes were considered showing maximum transcription levels higher than 25 normalized read counts. Red colors represent positive correlation, blue colors represent negative correlation and grey/light colors represent no/weak correlation between the selected genes.


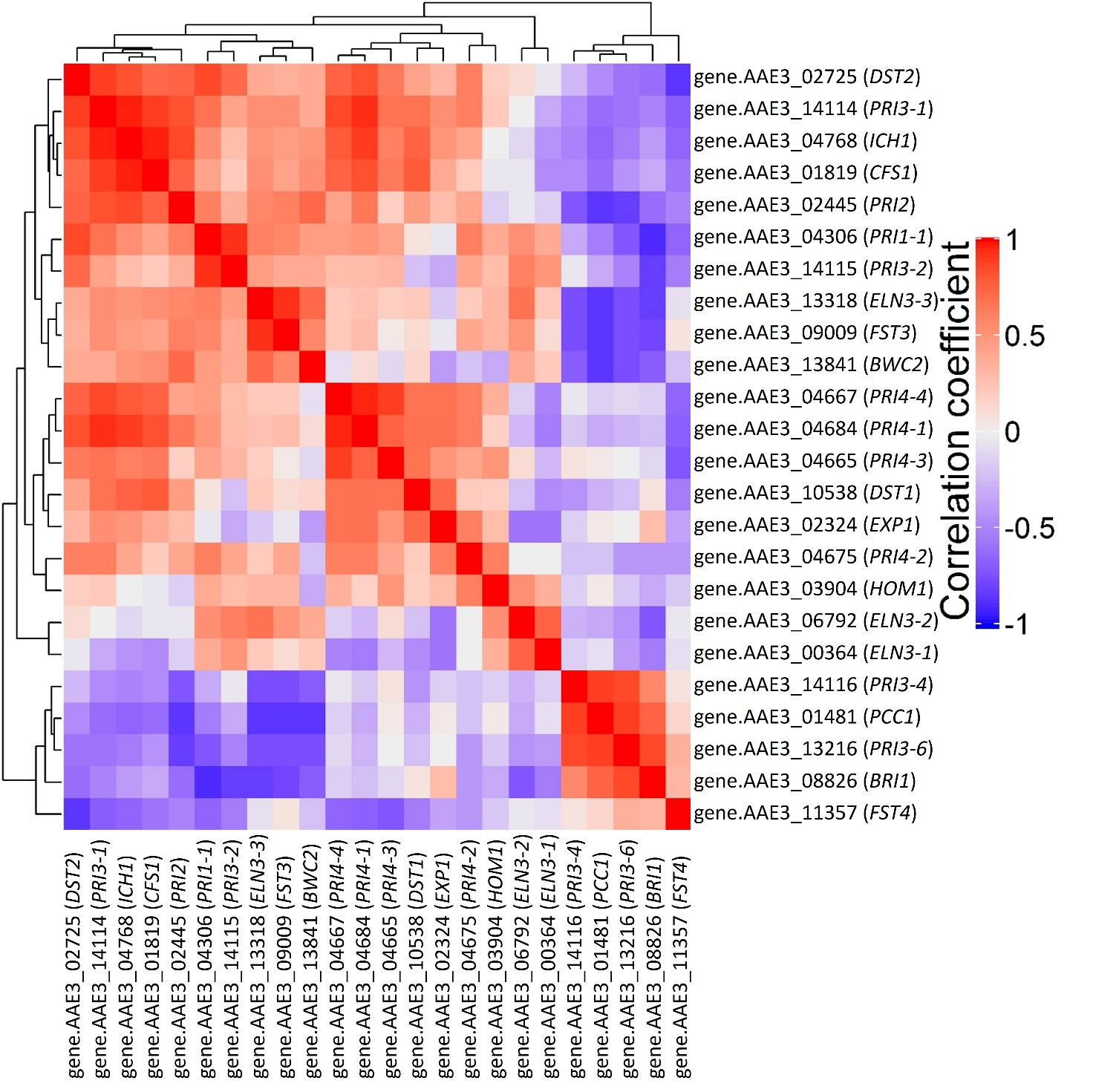


Figure S3: Correlation of the expression of putative C. aegerita homologs of fruiting-related genes (FRGs) in mycelium samples. Only genes were considered showing maximum transcription levels higher than 25 normalized read counts. Red colors represent positive correlation, blue colors represent negative correlation and grey/light colors represent no/weak correlation between the selected genes.


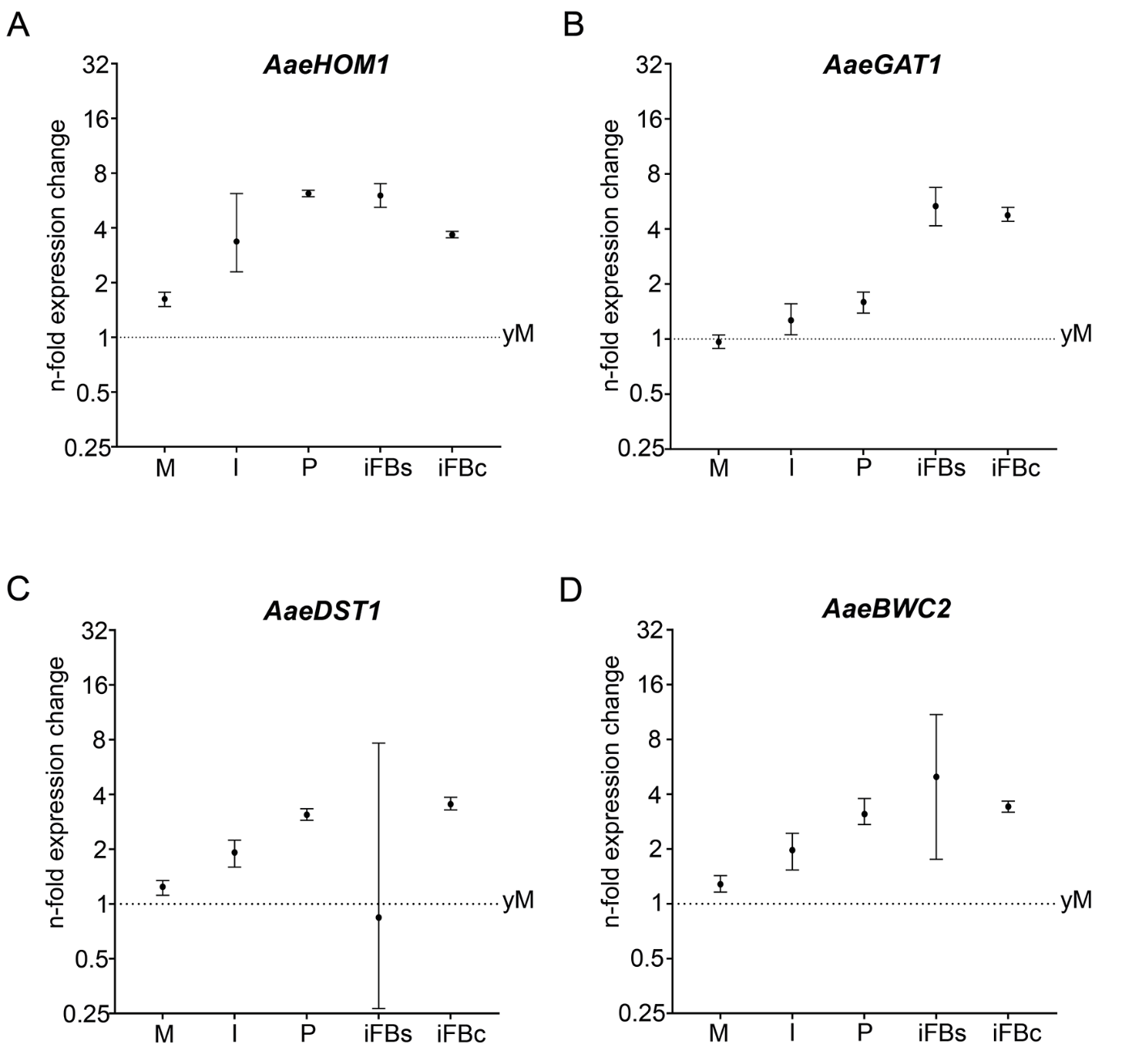


Figure S4: Relative qRT-PCR based expression level assessment with C. aegerita orthologs of four well-known fruiting-related genes (FRGs) during fruiting of C. aegerita. Expression changes are shown on a log 2 scale. The error bars represent the standard deviation of three biological replicates. For initials and primordia each replicate was a separate RNA extraction of pooled individuals, each collected from sets of 2–3 plates (for initials) or 1 plate (for primordia) with no overlap between plates/sample pools. The dotted horizontal line represents the expression level of the respective gene in young (uninduced) mycelium (yM) as the reference expression. Sampled C. aegerita AAE-3 materials: M, fruiting-primed mycelium 24 h to 48 h before emergence of fruiting body (FB) initials; I, FB initials; P, primordia; iFBs, immature FB stipe; iFBc, immature FB cap. A-B Expression of AaeHOM1 (gene ID AAE3_03904) and AaeGAT1 (gene ID AAE3_00943). C-D Expression of AaeDST1 (gene ID AAE3_10538) and AaeBWC2 (gene ID AAE3_13841).

**Table S4: RT-qPCR primers for the fruiting-related genes (FRGs) HOM1, GAT1, BWC2 and DST1**

| **Primer** | **Sequence 5’-3’** |
| --- | --- |
| qhom1_f | TCGCTCTTGCTAAGATGCTG |
| qhom1_r | CCCTGACTCGACATCGTAAAC |
| qgat1_f | GTCCTCTGTCTCATCGTCTG |
| qgat1_r | GAGAGCGGGAATGAGTGC |
| qbwc2_f | CTTGACAGTGAGGGTACAGC |
| qbwc2_r | AAGGGTCTTCGGTCCTAGAG |
| qdst1_f | CCTATCACCCATTTCCCCATC |
| qdst1_r | CAATCGCCAGCTCTTCAAAC |
