# Supplementary material for: Transcriptome of different fruiting stages in the cultivated mushroom Cyclocybe aegerita suggests a complex regulation of fruiting and reveals enzymes putatively involved in fungal oxylipin biosynthesis
Source: BMC Genomics. 2021 May 4;22:324. doi: 10.1186/s12864-021-07648-5 (PMC8097960; doi:10.1186/s12864-021-07648-5)
Supplement: Supplementary file 4 — Additional file 4: Terpenoid biosynthesis. Figure S5. Expression of genes involved in the mevalonate pathway and of the sesquiterpene synthases Agr1 to Agr9 in C. aegerita. Figure S6. Transcription levels of the sesquiterpene synthases Agr1, Agr4, Agr8 and Agr9. [file 12864_2021_7648_MOESM4_ESM.docx]

**Terpenoid biosynthesis**


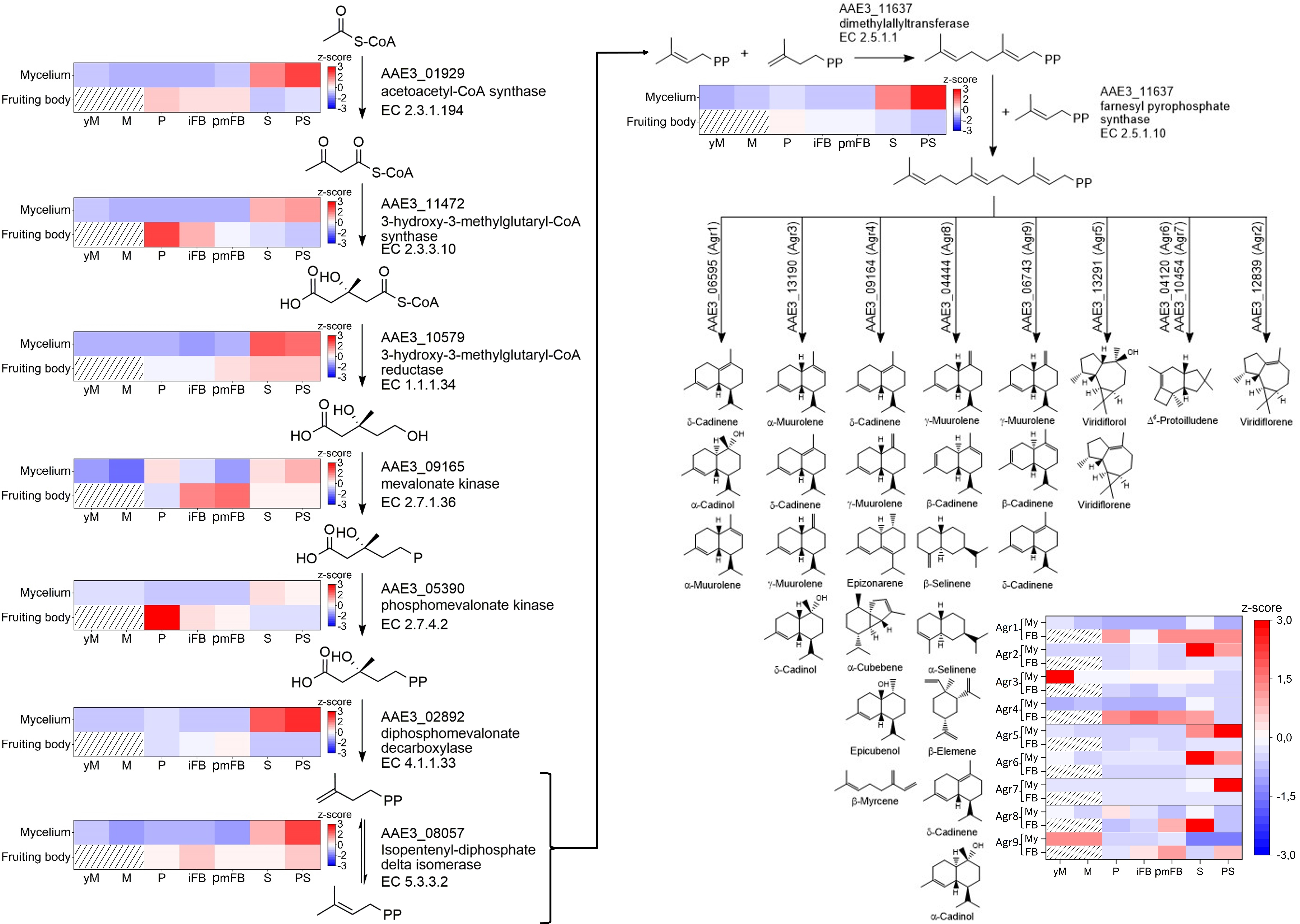


Figure S5: Expression of genes involved in the mevalonate pathway and of the sesquiterpene synthases (STSs) Agr1 to Agr9 in the mycelium and fruiting bodies of *C. aegerita*. Normalized read counts per gene were transformed to z-score values (respective scale to the right). Red colors indicate transcriptional upregulation while blue colors represent downregulation. White colors indicate a z-score of zero and hatched areas show an absence of sampling due the non-applicability. Interestingly, the transcription levels of genes involved in the mevalonate pathway were typically higher in the mycelium than in the fruiting bodies, except for the 3-hydroxy-3-methylglutaryl-CoA synthase gene (AAE3_11472), the mevalonate kinase gene (AAE3_09165) and the phosphomevalonate kinase gene (AAE3_05390). The farnesyl pyrophosphate synthase gene (AAE3_11637), responsible for the biosynthesis of the precursor of sesquiterpenes, was in both sample types highly expressed, especially in mycelium during late developmental stages (over 7,000 normalized read counts) and in primordia (over 2,900 normalized read counts). yM: young (uninduced) mycelium (day 10 post inoculation, p.i.); M: fruiting-primed mycelium (day 14 p.i.); P: primordia (day 18 p.i.); iFB: immature fruiting bodies (day 20 p.i.); pmFB: premature fruiting bodies (day 22 p.i.); S: sporulation (day 24 p.i.); PS: post sporulation (day 28 p.i.).


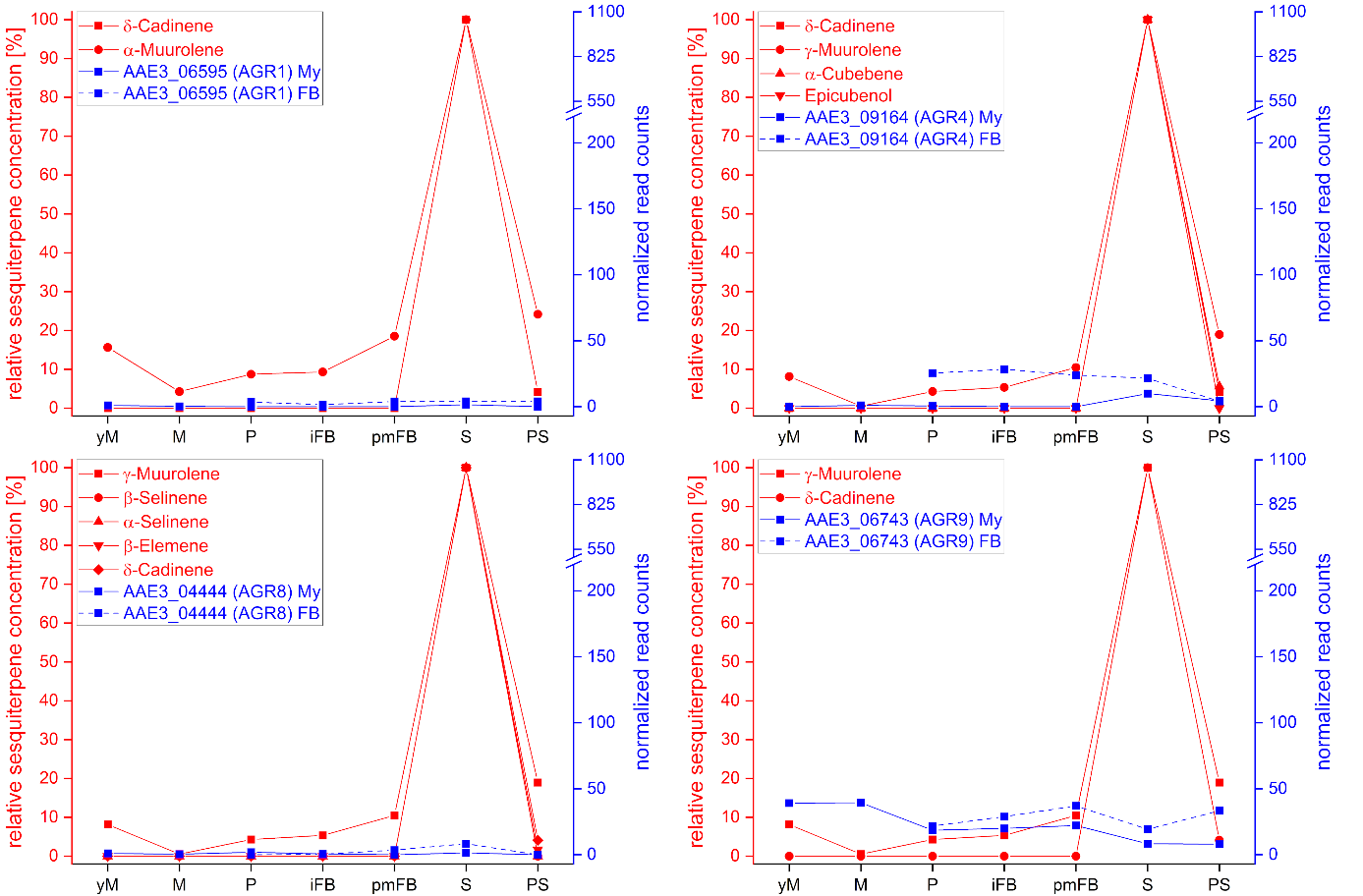


Figure S6: Transcription levels of the genes coding for the sesquiterpene synthases (STSs) Agr1, Agr4, Agr8 and Agr9 (blue) in the mycelium (My) and in fruiting bodies (FB) during different developmental stages of *C. aegerita* as well as the relative concentrations of the corresponding sesquiterpenes (red) in the HS of *C. aegerita*. Interestingly, compared to the STSs Agr2, Agr3, Agr5, Agr6 and Agr7, Agr9 coded by the gene AAE3_ 06743, involved in the synthesis of γ-muurolene and δ-cadinene, displayed in the mycelium as well as in FB quite constant transcription levels, ranging between 8 and 40 normalized read counts. This also applied for the transcription level of AAE3_09164 (*AGR4*) in FB whereas in the mycelium the highest transcription level was with 10 normalized read counts during sporulation quite low. Of all STS investigated in *C. aegerita* AAE-3, AAE3_06595 (*AGR1*) and AAE3_04444 (*AGR8*) showed the lowest transcription levels, not exceeding 9 normalized read counts in the examined developmental stages, neither in the mycelium nor in the plectenchmyatic samples of the different fruiting body stages. yM: young (uninduced) mycelium (day 10 post inoculation, p.i.); M: fruiting-primed mycelium (day 14 p.i.); P: primordia (day 18 p.i.); iFB: immature fruiting bodies (day 20 p.i.); pmFB: premature fruiting bodies (day 22 p.i.); S: sporulation (day 24 p.i.); PS: post sporulation (day 28 p.i.).
