# Supplementary material for: Transcriptome of different fruiting stages in the cultivated mushroom Cyclocybe aegerita suggests a complex regulation of fruiting and reveals enzymes putatively involved in fungal oxylipin biosynthesis
Source: BMC Genomics. 2021 May 4;22:324. doi: 10.1186/s12864-021-07648-5 (PMC8097960; doi:10.1186/s12864-021-07648-5)
Supplement: Supplementary file 5 — Additional file 5: The fatty acid metabolism. Figure S7. Expression of genes putatively involved in the fatty acid biosynthesis of C. aegerita. [file 12864_2021_7648_MOESM5_ESM.docx]

**The fatty acid metabolism**

The fatty acid synthase (FAS) is a multifunctional enzyme playing a key role in the cytosolic fatty acid synthesis. In fungi, the FAS complex normally consists of eight enzyme domains including an acetyltransferase (AT), an enoyl reductase, a dehydratase (DH), malonyl/palmitoyltransferase (MPT), an acyl carrier protein (ACP), a ketoreductase (KR), a ketosynthase (KS) and a phosphopantetheinyl transferase (PPT) [1, 2]. The FAS complex differs among fungi. In Basidiomycota, it tends to be a monomeric FAS protein encoded by a single gene whereas in Ascomycota the FAS complex is a *α*_6_*β*_6_ oligomer with the eight domains encoded in two genes [3–5]. The cytosolic fatty acid synthesis is initialized by the binding of acetyl-CoA to a phosphopantetheinyl linker of ACP catalyzed by AT and the binding of a malonyl-CoA elongation unit by means of MPT followed by condensation, accompanied by a decarboxylation step, to β-ketoacyl-SACP derivatives catalyzed by the KS domain [2] (Figure S7). Malonyl-CoA is provided by an acetyl-CoA carboxylase catalyzing the carboxylation of acetyl-CoA. Subsequently, multiple reduction steps involving KR, DH and ER occur, lead to the corresponding acyl-SACP. Repetitions of this cycle and reversed loading reaction catalyzed by MPT result in the release of palmitoyl and stearoyl-CoA which can be further elongated catalyzed by β-ketoacyl-CoA synthases (elongases). Stearyl-CoA can be transformed successively into the polyunsaturated fatty acid linoleic acid by means of desaturases [6] (Figure S7). Genes coding for enzymes involved in fatty acid synthesis and processing were identified in the *C. aegerita* genome by means of BLAST search using amino acid sequences of already characterized fungal analogues. Generally, these enzymes were higher expressed in plectenchymatic samples (fruiting body stages) than in the mycelium with exception of the putative Δ^9^-fatty acid desaturase AAE3_10709 and the putative Δ^12^-fatty acid desaturase AAE3_00256 (Figure S7). Interestingly, many genes revealed the highest transcription levels in primordia and/or late fruiting body development stages including the putative acetyl-CoA carboxylase AAE3_00446, the putative FAS AAE3_09085, the putative elongase AAE3_08045, the putative Δ^9^-fatty acid desaturases AAE3_00260, AAE3_10708 (homolog of *Le*-FAD1 [7]) and AAE3_07049 as well as the putative Δ^12^-fatty acid desaturases AAE3_00132, AAE3_00257, AAE3_03586 (homolog of *Le*-FAD2 [8]) and AAE3_12354.

Nonetheless, in our study, most genes involved in fatty acid biosynthesis and processing also showed a decent transcription over 100 normalized read counts in the mycelium. An enhanced biosynthesis of fatty acid in younger mycelium stages and subsequent storage is quite likely taking the comparably high fat content in the mycelium of various fungi into account [9, 10]. A crucial step in the biosynthesis of fungal volatile oxylipins is the formation of unsaturated fatty acids, especially linoleic acid, from saturated fatty acids catalyzed by desaturases [3] (Figure S7). Sakai et al. characterized a Δ^9^-fatty acid desaturase (FAD1) [7] and a Δ^12^-fatty acid desaturase (FAD2) [8] from *L. edodes* and compared the levels of expression in mycelium, primordia and mature fruiting bodies. Depending on the cultivation conditions, *FAD1* showed an increase in transcription by 4.1 and 6.0-fold and *FAD2* by 3.5 and 4.2-fold in primordia and fruiting bodies compared to the mycelium [7, 8] which is in good agreement with our results. Additionally, Wang et al. reported for *L. edodes* a high and quite constant expression of *FAD2* in three different developmental stages of fruiting bodies [11] counting as well for young fruiting stages of *C. aegerita*, nonetheless it is worth to mention that in primordia, a developmental stage not investigated by Wang et al., we observed remarkably higher transcription of AAE3_10708 (putative homolog of *Le-*FAD1) and especially of AAE3_03586 (putative homolog of *Le-*FAD2) than in all other developmental stages.

**References**

1. Schweizer E, Hofmann J. Microbial Type I Fatty Acid Synthases (FAS): Major Players in a Network of Cellular FAS Systems. Microbiol Mol Biol Rev. 2004;68:501–17.

2. Dickschat JS. Fungal volatiles – a survey from edible mushrooms to moulds. Nat Prod Rep. 2017;34:310–28.

3. Reich M, Göbel C, Kohler A, Buée M, Martin F, Feussner I, et al. Fatty acid metabolism in the ectomycorrhizal fungus *Laccaria bicolor*. New Phytol. 2009;182:950–64.

4. Antelo L, Schlipp A, Hof C, Eisfeld K, Berg H, Hornbogen T, et al. The Fatty Acid Synthase of the Basidiomycete *Omphalotus olearius* is a Single Polypeptide. Z Für Naturforschung C. 2014;64:244–250.

5. Jenni S, Leibundgut M, Maier T, Ban N. Architecture of a Fungal Fatty Acid Synthase at 5 Å Resolution. Science. 2006;311:1263–7.

6. Beccaccioli M, Reverberi M, Scala V. Fungal lipids: biosynthesis and signalling during plant-pathogen interaction. Front Biosci Landmark Ed. 2019;24:172–85.

7. Sakai H, Kajiwara S. A stearoyl-CoA-specific Delta 9 fatty acid desaturase from the basidiomycete *Lentinula edodes*. Biosci Biotechnol Biochem. 2003;67:2431–7.

8. Sakai H, Kajiwara S. Cloning and functional characterization of a Δ12 fatty acid desaturase gene from the basidiomycete *Lentinula edodes*. Mol Genet Genomics. 2005;273:336–41.

9. Sumner JL. The fatty acid composition of basidiomycetes. N Z J Bot. 1973;11:435–42.

10. Cohen N, Cohen J, Asatiani M, Varshney V, Yu H-T, Yang Y-C, et al. Chemical Composition and Nutritional and Medicinal Value of Fruit Bodies and Submerged Cultured Mycelia of Culinary-Medicinal Higher Basidiomycetes Mushrooms. Int J Med Mushrooms. 2014;16:273–91.

11. Wang Y, Zeng X, Liu W. De novo transcriptomic analysis during *Lentinula edodes* fruiting body growth. Gene. 2018;641:326–34.


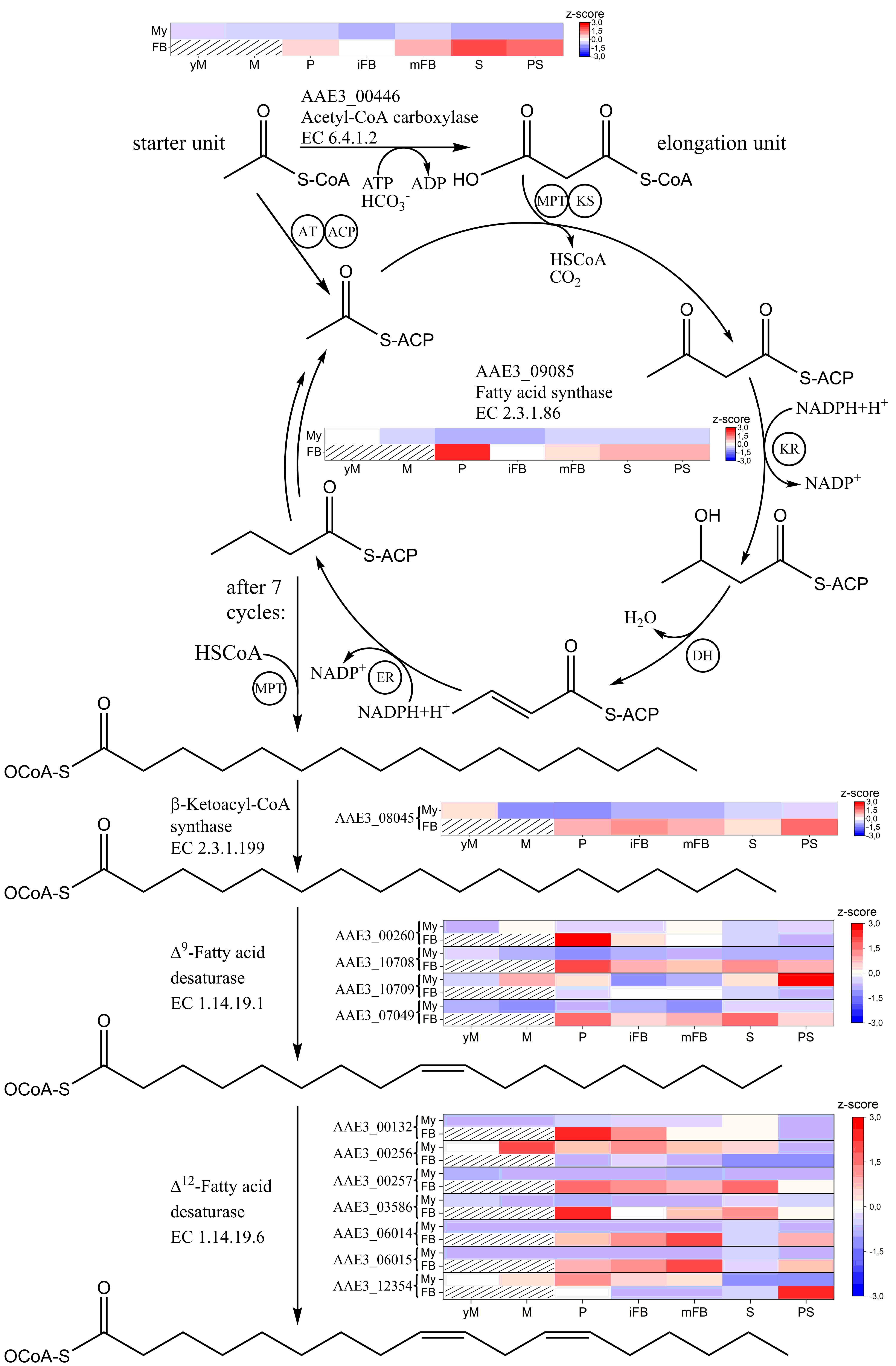


Figure S7: Expression of genes putatively involved in the fatty acid biosynthesis in the mycelium (My) and in fruiting bodies (FB) of *C. aegerita*. Normalized read counts were transformed to z-score values (respective scale to the right) whereby only genes were considered showing maximum transcription levels higher than 25 normalized read counts. Red colors indicate transcriptional upregulation while blue colors represent downregulation. White colors indicate a z-score of zero and hatched areas show an absence of sampling due the non-applicability. yM: young (uninduced) mycelium (day 10 post inoculation, p.i.); M: mycelium (day 14 p.i.); P: primordia (day 18 p.i.); iFB: immature fruiting bodies (day 20 p.i.); pmFB: premature fruiting bodies (day 22 p.i.); S: sporulation (day 24 p.i.); PS: post sporulation (day 28 p.i.).
