# Supplementary material for: Transcriptome of different fruiting stages in the cultivated mushroom Cyclocybe aegerita suggests a complex regulation of fruiting and reveals enzymes putatively involved in fungal oxylipin biosynthesis
Source: BMC Genomics. 2021 May 4;22:324. doi: 10.1186/s12864-021-07648-5 (PMC8097960; doi:10.1186/s12864-021-07648-5)
Supplement: Supplementary file 6 — Additional file 6: Figure S8. Proposed pathways for the enzymatic formation of fungal oxylipins. Figure S9. Transcription levels of putative HPLs as well as the relative concentrations of volatile oxylipins in the headspace of C. aegerita. Figure S10. Transcription levels of putative ADHs as well as the relative concentrations of volatile oxylipins in the headspace of C. aegerita. Figure S11. Transcription levels of putative ene-reductases as well as the relative concentrations of volatile oxylipins in the headspace of C. aegerita. [file 12864_2021_7648_MOESM6_ESM.docx]

**Enzymes acting on C8 oxylipins**


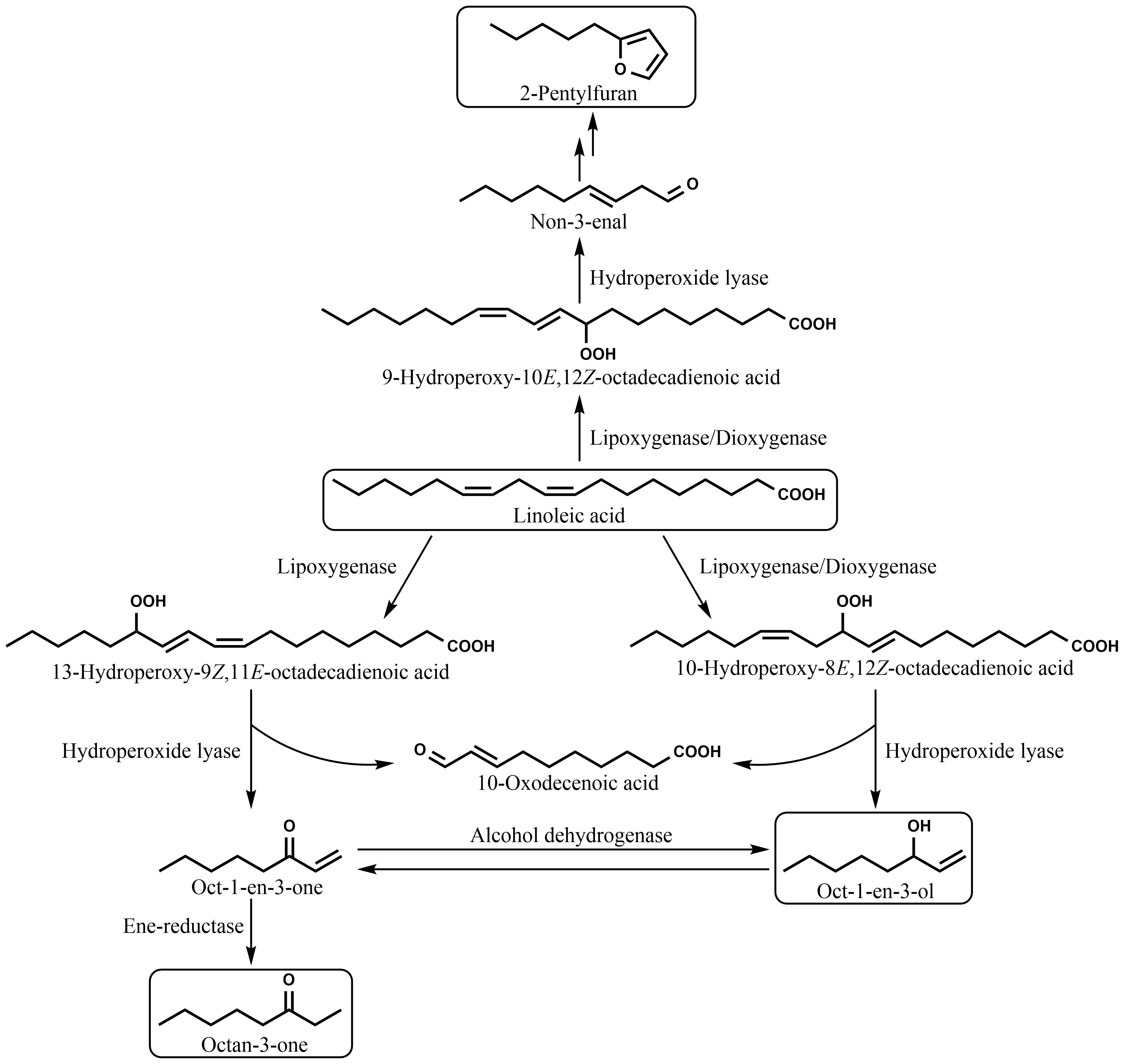


Figure S8: Proposed pathways for the enzymatic formation of fungal oxylipins.

Putative HPLs of *C. aegerita* were identified by means of BLAST search using protein sequences of characterized members of the CYP74 family in plants (Figure S9). Putative ADHs were identified using an ADH of the fungus *N. crassa* (Q9P6C8) ) proven to be able to oxidize octan-1-ol [1]. To reduce the number of putative ADHs to the essentials, only genes were considered showing maximum transcription levels higher than 300 normalized read counts. Analogously, putative ene-reductases were analyzed by means of BLAST search using sequences of characterized non-FMN ene-reductases of plants accepting *inter alia* non-2-enal and oct-1-en-3-one as substrates [2, 3] (*Nicotiana tabacum*, Q9SLN8; *Arabidopsis thaliana*, Q39172) and a fungal non-FMN ene-reductase (*Sporidiobolus salmonicolor*, A0A0D6ERK8). Additionally, sequences of fungal FMN depending old yellow enzyme (OYE) ene-reductases were used proven to be able to reduce amongst others citral (geranial) which shows some structural similarities with non-2-enal and oct-1-en-3-one [4, 5] (*Pichia stipites*, A3LT82), *Meyerozyma guilliermondii*, A5DR62). To reduce the number of putative ene-reductases to the essentials, only genes were considered showing maximum transcription levels higher than 300 normalized read counts (Figure S11). It is worth to mention that the putative OYE ene-reductases generally showed, compared to the putative non-FMN ene-reductases, low expression.

**References**

1. Park Y-C, San K-Y, Bennett GN. Characterization of alcohol dehydrogenase 1 and 3 from *Neurospora crassa* FGSC2489. Appl Microbiol Biotechnol. 2007;76:349–56.

2. Mansell DJ, Toogood HS, Waller J, Hughes JMX, Levy CW, Gardiner JM, et al. Biocatalytic Asymmetric Alkene Reduction: Crystal Structure and Characterization of a Double Bond Reductase from *Nicotiana tabacum*. ACS Catal. 2013;3:370–9.

3. Mano J, Torii Y, Hayashi S, Takimoto K, Matsui K, Nakamura K, et al. The NADPH:Quinone Oxidoreductase P1-ζ-crystallin in *Arabidopsis* Catalyzes the α,β-Hydrogenation of 2-Alkenals: Detoxication of the Lipid Peroxide-Derived Reactive Aldehydes. Plant Cell Physiol. 2002;43:1445–55.

4. Zhang B, Zheng L, Lin J, Wei D. Characterization of an ene-reductase from *Meyerozyma guilliermondii* for asymmetric bioreduction of α,β-unsaturated compounds. Biotechnol Lett. 2016;38:1527–34.

5. Bougioukou DJ, Walton AZ, Stewart JD. Towards preparative-scale, biocatalytic alkene reductions. Chem Commun. 2010;46:8558–60.


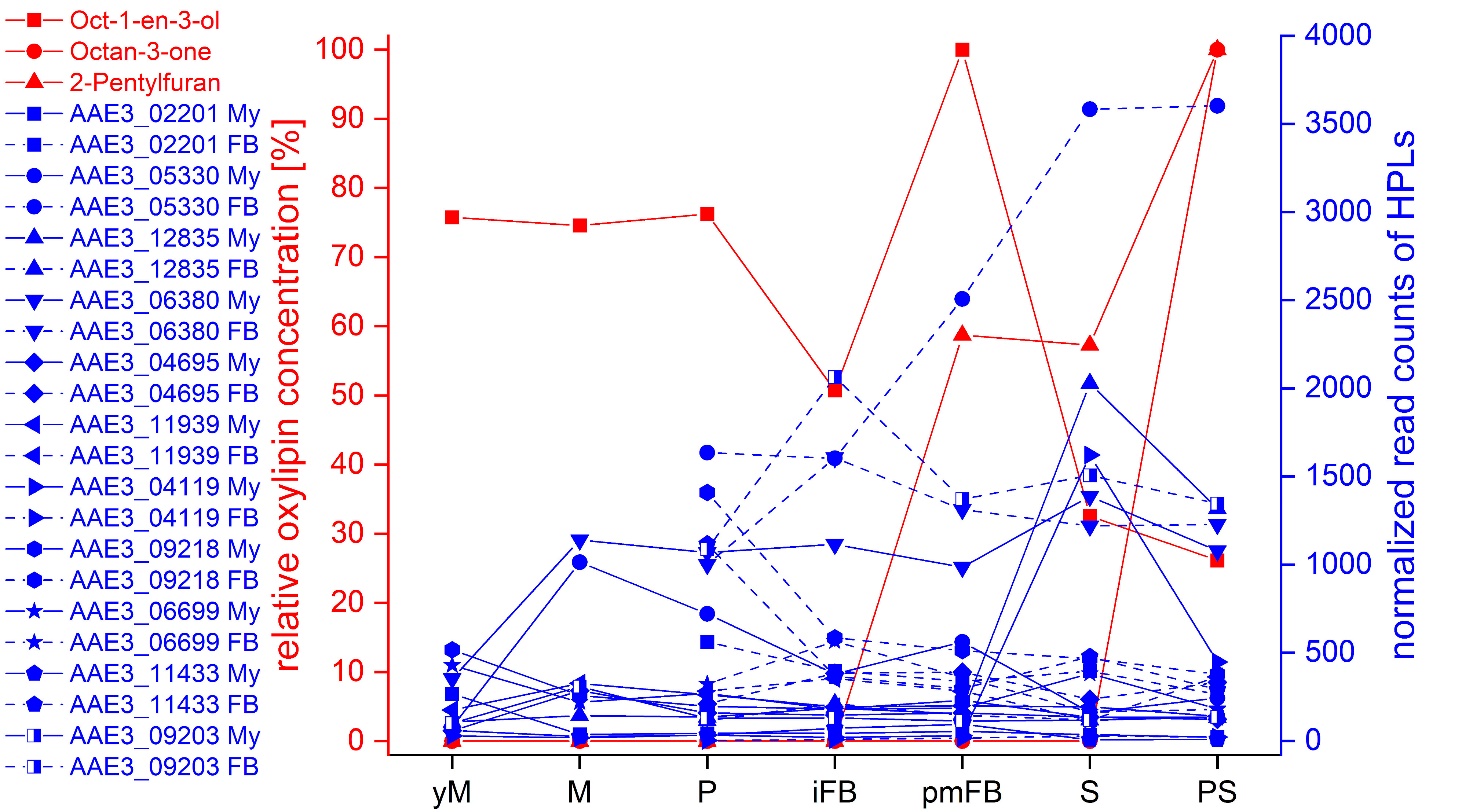


Figure S9: Transcription levels of genes coding for putative HPLs (blue) in the mycelium (My) and in fruiting bodies (FB) during different developmental stages of C. aegerita as well as the relative concentrations of volatile oxylipins (red) in the HS of C. aegerita. Only genes were considered showing maximum transcription levels higher than 300 normalized read counts. yM: young (uninduced) mycelium (day 10 post inoculation, p.i.); M: mycelium (day 14 p.i.); P: primordia (day 18 p.i.); iFB: immature fruiting bodies (day 20 p.i.); pmFB: premature fruiting bodies (day 22 p.i.); S: sporulation (day 24 p.i.); PS: post sporulation (day 28 p.i.).


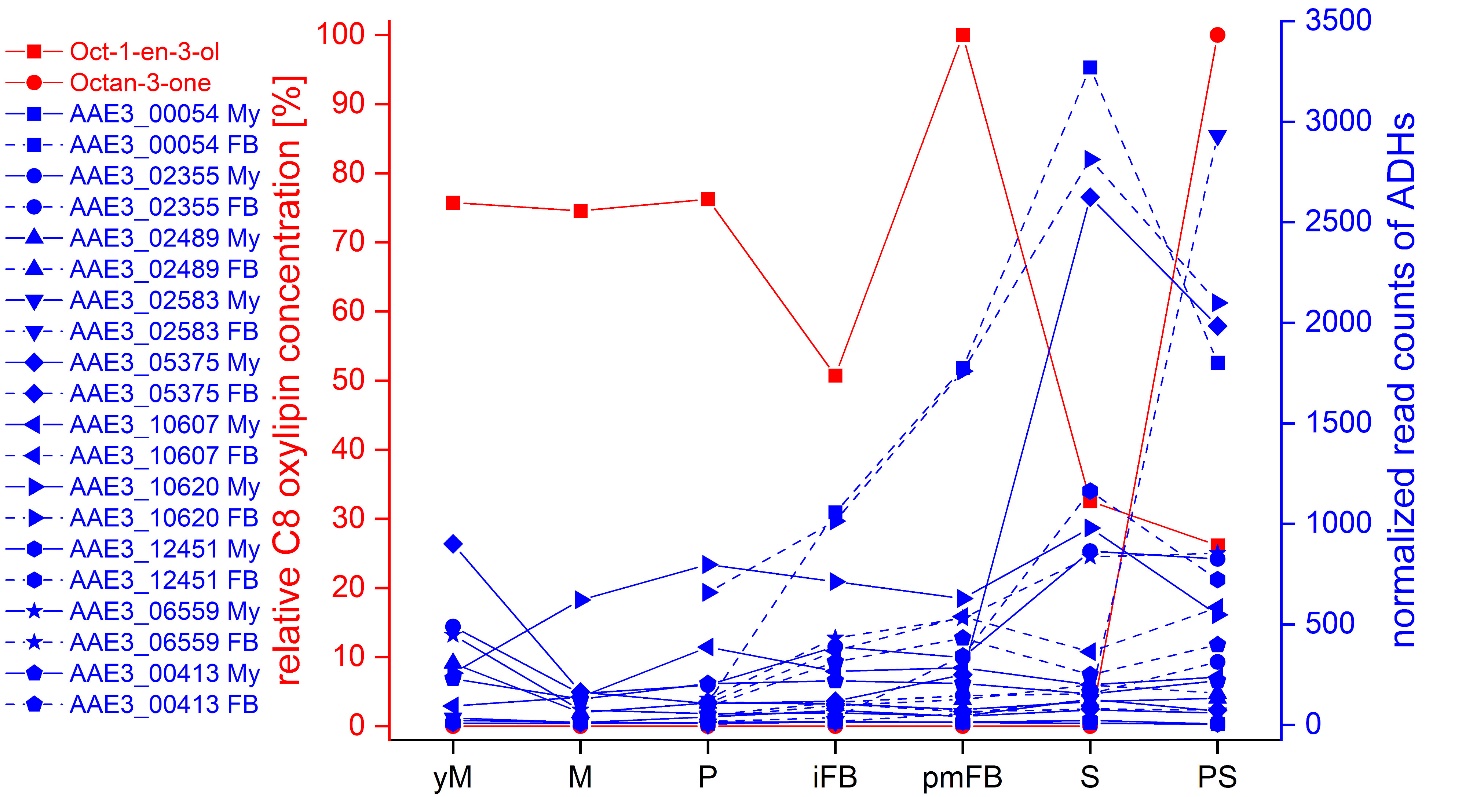


Figure S10: Transcription levels of genes coding for putative ADHs (blue) in the mycelium (My) and in fruiting bodies (FB) during different developmental stages of C. aegerita as well as the relative concentrations of volatile C8 oxylipins (red) in the HS of C. aegerita. Only genes were considered showing maximum transcription levels higher than 300 normalized read counts. yM: young (uninduced) mycelium (day 10 post inoculation, p.i.); M: mycelium (day 14 p.i.); P: primordia (day 18 p.i.); iFB: immature fruiting bodies (day 20 p.i.); pmFB: premature fruiting bodies (day 22 p.i.); S: sporulation (day 24 p.i.); PS: post sporulation (day 28 p.i.).


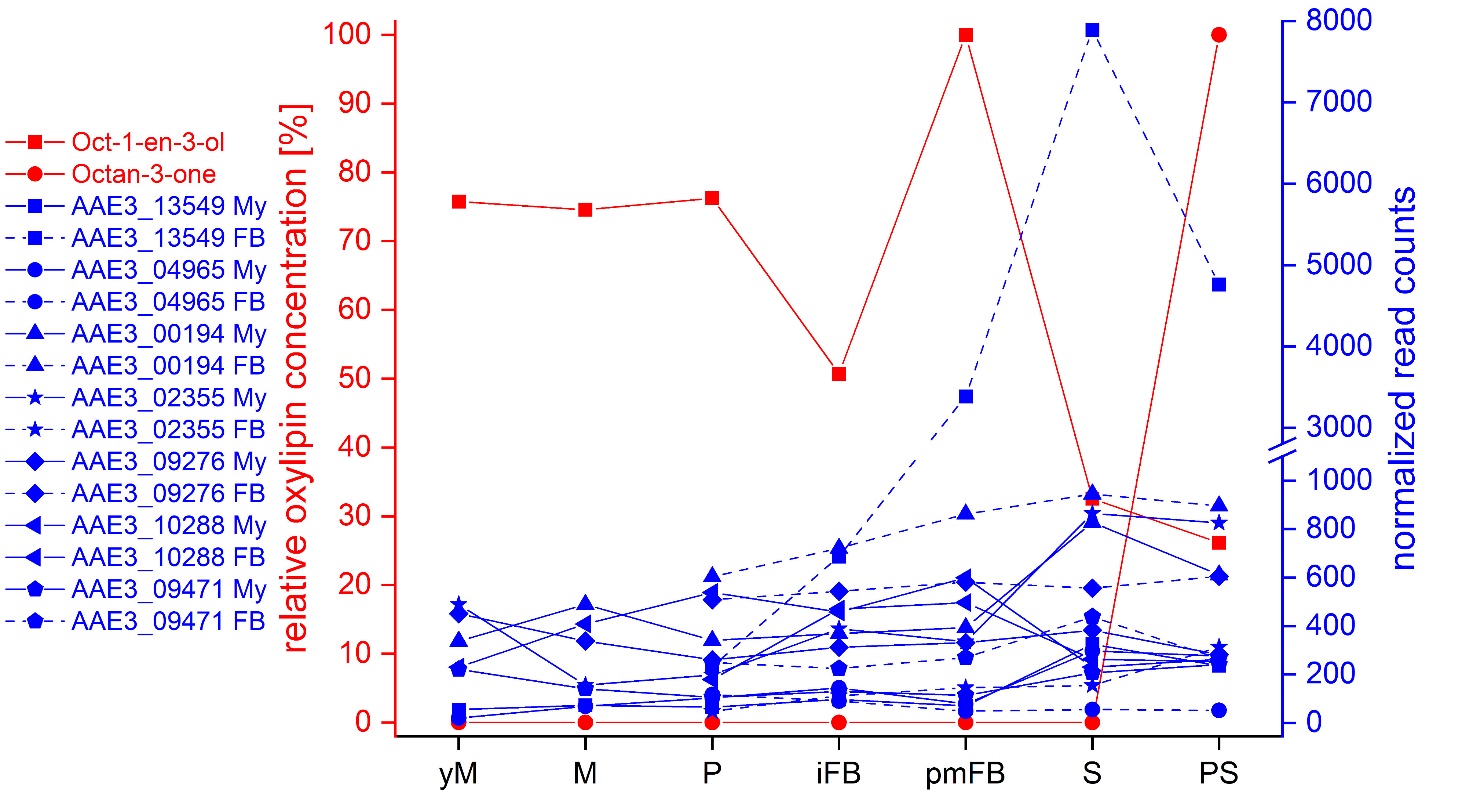


Figure S11: Transcription levels of genes coding for putative ene-reductases (blue) in the mycelium (My) and in fruiting bodies (FB) during different developmental stages of C. aegerita as well as the relative concentrations of volatile C8 oxylipins (red) in the HS of C. aegerita. Only genes were considered showing maximum transcription levels higher than 300 normalized read counts. yM: young (uninduced) mycelium (day 10 post inoculation, p.i.); M: mycelium (day 14 p.i.); P: primordia (day 18 p.i.); iFB: immature fruiting bodies (day 20 p.i.); pmFB: premature fruiting bodies (day 22 p.i.); S: sporulation (day 24 p.i.); PS: post sporulation (day 28 p.i.).
