# Supplementary material for: Transcriptome of different fruiting stages in the cultivated mushroom Cyclocybe aegerita suggests a complex regulation of fruiting and reveals enzymes putatively involved in fungal oxylipin biosynthesis
Source: BMC Genomics. 2021 May 4;22:324. doi: 10.1186/s12864-021-07648-5 (PMC8097960; doi:10.1186/s12864-021-07648-5)
Supplement: Supplementary file 7 — Additional file 7: Revealing putative enzymes of the oxylipin pathway in C. aegerita. Figure S12. Phylogenetic analysis of different DOXs. Figure S13. Partial amino acid sequence alignment of different DOXs. Figure S14. Phylogenetic analysis of different CYP74 proteins. Figure S15. Transcription levels of the putative DOX AAE3_13098 and the putative HPL AAE3_09203. Figure S16. Transcription levels of AAE3_04864 (LOX4) and the putative HPL AAE3_05330. Figure S17. Phylogenetic analysis of putative ADHs. Figure S18. Amino acid sequence alignment of ene-reductases. Figure S19. Transcription levels of the putative ADHs AAE3_00054 and AAE3_06559 as well as the putative ene-reductase AAE3_13549. [file 12864_2021_7648_MOESM7_ESM.docx]

**Revealing putative enzymes of the oxylipin pathway in *C. aegerita***

Putative DOXs were identified in the genome of *C. aegerita* by means of BLAST search using protein sequences of characterized DOXs from Ascomycetes including a 8R-DOX-7,8-LDS (linoleate diol synthase) (*Gaeumannomyces graminis*, AAD49559), a 9R-DOX (*Fusarium oxysporum*, EGU79548) and a 10R-DOX-CYP (cytochrome P450) (*Aspergillus fumigatus*, ABV21633). This way, two putative DOXs (AAE3_00407 and AAE3_13098) were found, being, as reported for other fungal 8-, 9- and 10-DOXs, fused to CYPs, which are usually catalytically functional [1]. Fungal DOX-CYPs consist of several subfamilies including *inter alia* 5,8- and 7,8-LDS, 10*R*-DOX-EAS (epoxy alcohol synthase), 9*S*- and 9*R*-DOX-AOS (allene oxide synthase), 8*S*- and 8*R*-DOX-AOS and 10*R*-DOX-CYP enzymes, the latter lacking the heme-thiolate ligand in the CYP domain. Therefore, this domain is proposed to be not functional [2, 3]. All DOX fusion proteins harbor the dioxygenase domain at the N-terminus whereas the P450 domain, responsible for the rearrangement of the N-terminally formed hydroperoxide fatty acid, is located at the C-terminus [4]. Phylogenetic analysis revealed that fungal DOXs cluster in different groups, with the two putative DOXs from *C. aegerita* assembling with putative DOXs of the basidiomycete fungus *Rhizoctonia solani* indicating sequence and potentially functional difference between DOXs from Basidiomycota and the so far characterized DOXs from Ascomycota (Supplementary Figure S12).

**References**

1. Oliw EH. Polyunsaturated C-18 fatty acids derivatized with Gly and Ile as an additional tool for studies of the catalytic evolution of fungal 8-and 9-dioxygenases. Biochim Biophys Acta-Mol Cell Biol Lipids. 2018;1863:1378–87.

2. Oliw EH. Biosynthesis of Oxylipins by *Rhizoctonia solani* with Allene Oxide and Oleate 8S,9S-Diol Synthase Activities. Lipids. 2018;53:527–37.

3. Brodhun F, Schneider S, Göbel C, Hornung E, Feussner I. PpoC from *Aspergillus nidulans* is a fusion protein with only one active haem. Biochem J. 2010;425:553–65.

4. Hoffmann I, Oliw EH. Discovery of a linoleate 9S-dioxygenase and an allene oxide synthase in a fusion protein of *Fusarium oxysporum*. J Lipid Res. 2013;54:3471–80.


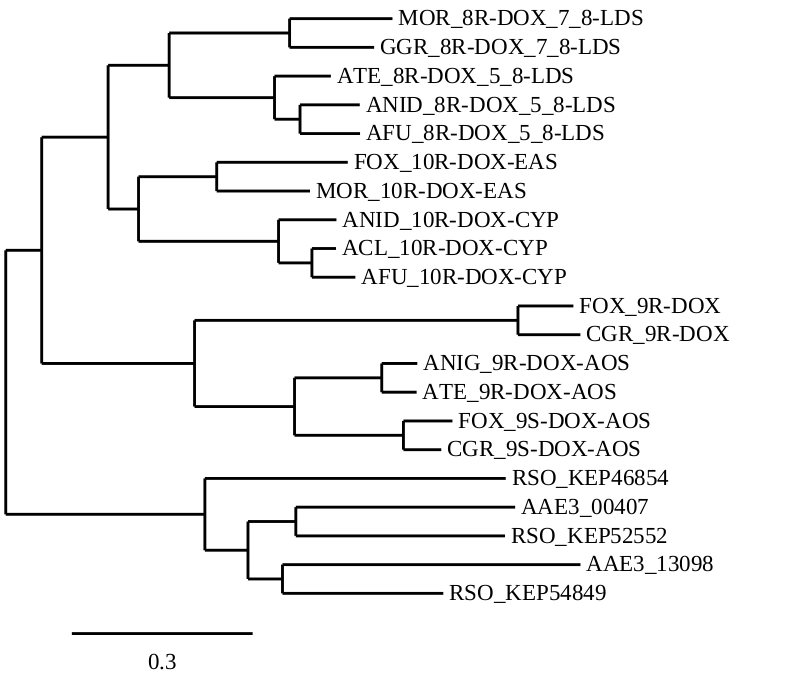


Figure S12: Phylogenetic analysis of different DOXs from Ascomycota and putative DOXs from the Basidiomycota *C. aegerita* and *Rhizoctonia solani*. MOR: *Magnaporthe oryzae*, GGR: *Gaeumannomyces graminis*, ATE: *Aspergillus terreus*, ANID: *Aspergillus nidulans*, AFU: *Aspergillus fumigatus*, FOX: *Fusarium oxysporum*, ANIG; *Aspergillus niger*, ACL: *Aspergillus clavatus*, CGR: *Colletotrichum graminicola*, RSO: *Rhizoctonia solani*. MOR_8R-DOX_7_8-LDS (EHA52010), GGR_8R-DOX_7_8-LDS (AAD49559), ATE_8R-DOX_5_8-LDS (AGA95448), ANID_8R-DOX_5_8-LDS (EAA65132), AFU_8R-DOX_5_8-LDS (EDP50447), FOX_10R-DOX-EAS (EGU86021), MOR_10R-DOX-EAS (EHA53428), ANID_10R-DOX-CYP (AY613780), ACL_10R-DOX-CYP (EAW09782), AFU_10R-DOX-CYP (ABV21633), FOX_9R-DOX (EGU79548, FOXB_09952), CGR_9R-DOX (EFQ36675, GLRG_11821), ANIG_9R-DOX-AOS (EHA25900), ATE_9R-DOX-AOS (AGH14485), FOX_9S-DOX-AOS (EGU88194), CGR_9S-DOX-AOS (EFQ27323).

A)

FOX_9R-DOX DLFDKLMAREE--GGRESQSGLSAMLIYHATIIIHDIFRTNDNDKNISDSSSYLDLSPLYGYTTEMQRKVRDDKYKLGLLKPDTFAEDRLLRQPPGVCIMLVMYNRYHNYAARQLLRINE 291

CGR_9R-DOX VLFDRLMAREN--GGRQSQSGLSSMLLYHATIIIHDIFRTNDNDKNLSDSSSYLDLSPLYGYTEEMVRKVRDNKYKLGLLKPDTFAEDRLLRQPPGVCIMLVMYNRYHNYAARQLLQINE 287

AAE3_13098 LVFDTLLKRDK---FEKHPAGISALFFAFADLVIHSIFNTNHTDWTINDASSYLDLSILYGSSDQEVDSIRKKD-GSGALYEDVFADKRLLMMPPASCALLVLLSRNHNFIAQRIRDINE 362

AAE3_00407 LIFDTLLRRQG---FKKHPGGLSSLMFSFATLVIHSVFRTSHRDWSINETSSYVDLAPLYGNNQKEQDRLRIRD-GRGLLYPDVFSEDRLLLLPPAVCTLLVLFSRNHNYIAKKLLEINE 282

RSO_KEP52552 LVFECLLKRDK---VTPHPAGLSAMFFSFATLVIHTCFRTNHRDVTINETSSYVDLAPLYGNNQEDQDSVRRWD-GTGRLKEDVFTENRLLFLPPAVCTLLVLFCRNHNFIARKLYLINE 297

RSO_KEP54849 LIFDTLLRRDT-PGGKEHPAGVSSLLFSFAVLIIHSLFRTAPSSMYINDTSSYLDLSPLYGHSFEAQMKVRRND-GTGRLYEDVFAENRLLLMPPAVSALLIMFSRNHNYIATKLLQINE 348

RSO_KEP46854 MVVDELLTASSSTPRDLHPGGNSSLTFAFASLVTHSLFRTDPSDWGKNNTSSYLDLSPLYGSNQAEQDLVRVKD-GRGLLHPDTFSEGRLVFLPPAAAALLVMWNRNHNFIANNILKINE 239

ANIG_9R-DOX-AOS LLFDLLMARDD-TTFRENPAGISSVLFYHASIIIHDVFCTNRRDPNISDTSSYLDLAPLYGSSYEDQLRVRTMQ--RGMLKPDTFHEKRLLGQPPGVNVILVMYNRFHNYVADVLLKINE 348

ATE_9R-DOX-AOS LLFDLLMARDD-STFKENPAGISSMLFYHASIIIHDIFRTNRRDPNISDTSSYLDLAPLYGSSLEDQLKVRTME--KGMLKPDTFHEKRLLGQPAGVNVILVMYSRFHNYVADMLLKINE 348

FOX_9S-DOX-AOS LLFDLLMARDE-TTFQENPAGISSMLFYHAAIIIHDIFRTNRTDMNKSDTSSYLDLAPLYGSSLKDQHEIRTMK--EGKLKPDTFHEKRLLGQPAGVNVMLVLYSRFHNYVADILLKINE 319

CGR_9S-DOX-AOS LLFDLLMARDD-STFKENPAGISSMLFYHASIIIHDIFRTNRTDMNKSDTSSYLDLAPLYGSSLKDQLEIRTMK--EGMLKPDTFHERRLLGQPAGVNAMLVLYNRFHNYVCDILLKINE 319

MOR_8R-DOX(7,8-LDS) TIFDTLMARDP-AKFRPHPNQISSVLFYFATIITHDIFQTSSRDPSINLTSSYLDLSPLYGRNLEEQLSVRAMK--DGLLKPDTFCSKRVHGFPPGVGVLLIMFNRFHNYVVTSLAKINE 296

GGR_8R-DOX(7,8-LDS) TIFDTLMVRDP-AKFRPHPNKISSMLFYLATIITHDIFQTSPRDFNINLTSSYLDLSPLYGRNHDEQMAVRTGK--DGLLKPDTFSSKRVIGFPPGVGAFLIMFNRFHNYVVTQLAKINE 286

ANID_10R-DOX-CYP LVFDTLFAR---QKFTPHPNKVSSLFFDWASLIIHDIFQTDYRDYNKNKTSAYLDLAILYGDVQEEQDLVRTHK--DGKLKPDSFSEPRLQAFPAACCVLLVMLNRFHNYVVEELAAINE 337

ACL_10R-DOX-CYP LVFDTLFAR---QSFKPHPNNVSSLFFDWASLIIHDIFQTDYRNPHVNKTSGYLDLSILYGDVQEEQNLIRTFE--GGRLKTDSFSEPRLQAFPAACCVLLVMLNRFHNHVVEQLAAINE 332

AFU_10R-DOX-CYP LVFDTLFAR---QTFKPHPNKVSSVFFYWASLIIHDIFQTDYKNPNMNKTSGYLDLSILYGDVQEEQNLIRTFK--DGKLKPDSFSEPRLQAFPATCCVLMVMLNRFHNYAVEQLAAINE 336

ATE_8R-DOX(5,8-LDS) ALFDSLLAR---KDFKEHPNKISSVLFYIASIIIHDLFQTDHRDSSINRTSSYLDLSPLYGNNQDEQYLMRTFK--DGKLKPDCFSSKRILGFPPGVGVLLIMFNRFHNYVVEQLAAVNE 276

ANID_8R-DOX(5,8-LDS) TIFDCLLRR---KEYREHPNKISSVLFYLASIIIHDLFQTDPKDNSVSKTSSYLDLSPLYGNNQDEQNLVRTFK--DGKLKPDCFATKRVLGFPPGVGVLLIMFNRFHNYVVDQLAAINE 285

AFU_8R-DOX(5,8-LDS) TLFDCLLAR---KEYKEHPNKISSVLFYIASIIIHDLFETDRKDPAISLTSSYLDLSPLYGNNQQEQDLIRTFK--DGKLKPDCFSTKRVLGFPPDVGVVLIMFNRFHNYVVEKLAMINE 285

FOX_10R-DOX-EAS AIFESVFAR---DAFRKNPNNVSSILWYWATIIIHDLFWTNLQDPNQNDSSSYLDLAPLYGSTEKDRDSIRTFK--DGQLKPDCFADKRLIGNPPGVPILLIMFNRFHNHVATNLADINE 310

MOR_10R-DOX-EAS LIFDSIMGRTP-NSYRKHPNNVSSILWYWATIIIHDIFWTDPRDINTNKSSSYLDLAPLYGNSQEMQDSIRTFK--DGRMKPDCYADKRLAGMPPGVSVLLIMFNRFHNHVAENLALINE 359

:.: :: *:: * :: * * * . . :*.*:**: *** :* . * : * : *: * .::: * **. : :**

FOX_9R-DOX TLKLKDHDHQAAKDFEAACDEFKDAWEAAWNKQDDDLFNTARLITCGMYIQISVHDYLRALMGFHQFDTNFTLDPRADFDQKK-----TSRGIGNQVTVEFNLLYRFHCAISRKDEAYTE 525

CGR_9R-DOX GQKLERESPALLKSFENNCKDFKIAWQAACDKQDDDLFNTARLITQGMYVNISVHDYLRALMGFHQFDTNFTLDPRKDFDQKK-----TTRGIGNQVTVEFNLIYRFHCAISLRDEKYVE 522

AAE3_13098 ------------------------------MLQDDEIFHRARLVNCGYFMQIILGDYVGAILGLVRDETDWRLNPLMTMRELD--HDFAPTGEGNVCSVEFNLLYRWHATLSEPDTEWIT 463

AAE3_00407 ----------------------DPISKAKLVEQEEDIFQTARLINCGWFGMVVFSDYFSSILGLVRDGSSWSLTPFDEMRKED--HSLFERGKGNVCSVEFNCLYRWHATTSREDEQWTH 391

RSO_KEP52552 ----------------------RR------KQQDHVLFNTARLINVGFFVSIVLGDYLASILGIVREGSDWSLNPFQDIVQSE--RNHVPRGDGNSVSVEFNLLYHWHSTTSALDEEWTE 400

RSO_KEP54849 GTASTAYTTPAGSRFLGLDNQASRKARTPLEEQDHVIFNTARLINCGFFMHIIISDYIGAILGLTREGSSYSLNPLEEIRGSD--HQLVGRGEGNSCSVEFNLLYRWHATLSAADEKWTE 529

RSO_KEP46854 --------------------------PEKCARQDKEIFETARLINCGSFMSVVFGDYVAGFLGLSREGSSWGMQPFDPIQGSE--G-EVGRGQGNHCSVEFNLLYRWHAVTSEADEKWTD 341

ANIG_9R-DOX-AOS ----------------------EDAKRKALAKQDEDLFQVTRLIVNGLYVNISLHDYLRGLTNTHHSASDWTLDPRVAVSRAFD-ADGVPRGVGNQVSAEFNLLYRFHSVISRRDEQWTN 456

ATE_9R-DOX-AOS ----------------------EEARKKALAKQDEDLFQVARLVVNGLYVNISLHDYLRGLTNTHHSASDWTLDPRIAVGRTFD-PDGVPRGIGNQISAEFNLLYRFHSVISRRDEKWTN 456

FOX_9S-DOX-AOS ----------------------EEDKAKAIAKQDHDLFNVARLITGGLYINICLHDYLRAITNTHHSASDWTLDPRVAIDKQFD-GDGVPRGVGNQVSVEFNLLYRFHSCISKRDEKWIN 429

CGR_9S-DOX-AOS ----------------------PEDRAKAVAKQDHDLFNTARLIVGGLYINISLHDYLRAITNTHHSKSDWTLDPRVEIGKQFD-GEGVPRGVGNQVSVEFNLLYRFHSCISKKDERWID 429

MOR_8R-DOX(7,8-LDS) ------------------------DDTAAWEKYDNDLFQTGRLITCGLYVNIVLVDYVRTILNLNRVDSSWILDPRTEEGKSLL-SKPTPEAVGNQVSVEFNLIYRWHCGMSQRDDKWTT 400

GGR_8R-DOX(7,8-LDS) -----------------------PDDTAGWETYDNSLFQTGRLITCGLYINIVLGDYVRTILNLNRANTTWNLDPRTKEGKSLL-SKPTPEAVGNQVSVEFNLIYRWHCTISERDDKWTT 391

ANID_10R-DOX-CYP ----------------------EEQAKKAWAKYDEDLFQTGRLITCGLFINITLYDYLRTIVNLNRVNSTWCLDPRAQMEGSA-----TPAGLGNQCSVEFNLAYRWHSAISANDEKWTE 442

ACL_10R-DOX-CYP ----------------------EDQAKKAWEKYDEDLFQTGRLITCGLYINITLYDYLRTIVNLNRTNSTWCLDPRAQMEGNN----TTPSGLGNQCSVEFNLAYRWHSAISANDEKWTE 438

AFU_10R-DOX-CYP ----------------------EEEAKKAWAKYDEDLFQTGRLITCGLYINITLYDYLRTIVNLNRTNSTWCLDPRAQMEGSH----TAPSGLGNQCSVEFNLAYRWHSATSATDEKWTE 442

ATE_8R-DOX(5,8-LDS) ------------------------SNDKEYAKYDNNLFQTGRLVTCGLYINIILKDYVRTILNINRTNSTWSLDPRMDMKDGLL-GDAAPLATGNQVSAEFNLIYRWHSCISQRDEKWTT 380

ANID_8R-DOX(5,8-LDS) ------------------------SNVDEYAKYDNNLFQTGRLVTCGLYANIILKDYVRTILNINRTDSTWSLDPRMEMKDGLL-GEAAAMATGNQVSAEFNVVYRWHACISKRDEKWTE 389

AFU_8R-DOX(5,8-LDS) ------------------------SDTAAYAKYDNDLFQTGRLVTCGLYVNIILKDYVRTILNINRTDSIWSLDPRSEMKDGLL-GRAAAQATGNQVAAEFNLVYRWHSCISQRDQKWTE 389

FOX_10R-DOX-EAS ----------------------PEAADAAWKKRDTELFETARLVTSGLYINITLIDYVRNIINLNRVDTTWTLDPRQEMGVSVGTKDLSESGTGNVVSAEFNLCYRWHSCLSEMDDKWVQ 420

MOR_10R-DOX-EAS ----------------------GEAREAAWKKYDNDLFQVARLVTSGLYINITLVDYVRNIVNLNRVDTTWTLDPRQDAGAHVGTADGAERGTGNAVSAEFNLCYRWHSCISEKDSKFVE 469

: :*. **: * : : . **. : . : : : : * . ** :.*** *::*. * * :

B)

FOX_9R-DOX GVFVAKQVLA-YEKDQSK--AA-AILLLVCLDFAYNAVVSFTATLDGYMRDLYAAADGRP 1106

CGR_9R-DOX GVFVARQVLK-HEPNRVR--AA-AILLLISLDFAYNAVVSFSATLDGFMEDLCQVANGDN 1134

AAE3_13098 SHMFLKKLWHKLGKNYTRTEFTAQ-VFAAVIPTVALYSQAVAHIVDFYLDQ--------- 987

AAE3_00407 YHDIVKRLY-ELGE--STDQLANT-ILALMVTAGTELVITITNALNVYLGS--------- 898

RSO_KEP52552 SFDFMRRLH-KSGK--SKDQLCNA-VLAAIVA-SFEFIPALINVVNFYLET--------- 904

RSO_KEP54849 AQAFLTNLY-ANKGDLTVEQLSYN-VFGCMIASVSNYAQAATQVVDFYLDD--------- 1050

RSO_KEP46854 SDAFHTALL-ASKK--PINDLVAM-ICGMLVSSTVNFAQATSHMVDFYMDE--------- 861

ANIG_9R-DOX-AOS GNNVVKQMME---MDMTAAETAEVCW-LTAVGGVGAPVGLVADVLQYYLRP--------- 973

ATE_9R-DOX-AOS GNNVAKEMME---MGMSAEEVADICW-LTAIGGVGTPSGVVANVLQYYFRY--------- 972

FOX_9S-DOX-AOS GLKLVEELLA---QGNNVDQVTDNLW-LTAFGGIGVPVTAFYEVLSFFLRP--------- 954

CGR_9S-DOX-AOS GHKLVEELLA---QGNSAEQVVDNMW-LTAFGGIGAPVTAFYEVLEYFLRR--------- 955

MOR_8R-DOX(7,8-LDS) GDLMLRRMIEAYGEGKSVKEAVYGQIMPSIAAGTANQTQIMAQCLDYYMSD--------- 961

GGR_8R-DOX(7,8-LDS) GDQLLQRMLSQ--DGRSIEETVSGTILPVVMAGTANQTQLLAQCLDYYLG---------- 952

ANID_10R-DOX-CYP GDQLIKRLAE---GGLSVSDITYGQILPTAVELVHGQAQMFTRVVEYYLN---------- 935

ACL_10R-DOX-CYP GAHMTKQLLE---NGLGASEITWSQILPTVIAMVPSQAQAFTQIIDFYLSK--------- 947

AFU_10R-DOX-CYP GVHLTKQLLE---NGLGAHEIAWAQFLPTVIAMVPAQAQAFTQIVDFYLSK--------- 951

ATE_8R-DOX(5,8-LDS) GIHMIQRLLA---SGLPASEIVWTHLLPTAGGMVANQGQLFSQCLDYYLSE--------- 893

ANID_8R-DOX(5,8-LDS) GIHMIQRLLD---SGLPATEIVWTHILPTAGGMVANQAQLFSQCLDYYLSE--------- 902

AFU_8R-DOX(5,8-LDS) GVHMIQRLLD---SGMPAPEIVWTHVLPTAGGMVANQAQLFSQSLDYYLSE--------- 902

FOX_10R-DOX-EAS GENMAKGLKK---AGLSTEDIVWSQILPTAGAMVPNQAQVFAQTLDWYLSP--------- 929

MOR_10R-DOX-EAS GKTMIKGLKA---HGLSDYDIAWSHVVPTSGAMVPNQAQVFAQAVDYYLSP--------- 980

FOX_9R-DOX QVLM---SQLSIADK----F----GVFAPRRVATISLTSMIKFVAQMKNPRRGHDAQGKL 1251
CGR_9R-DOX EFLT---LQLSIADK----Y----AVFSPRRFTPLSQAQMIKFIALTRNTRRGPAAQGEL 1272
AAE3_13098 -------DYLRAASNPGLASFIGETGLLTSEFFQSTVPGVLAAIFKLKDLQRGPGLSGSF 1126
AAE3_00407 SLTRPAKDRLSADGAFNY-LGEGLTVKVKLSF-------------DMLPAVT---DNAPL 1015
RSO_KEP52552 DPRRPVNNY-------TL-MGDGLHRCFTDDFVHSTMACAIRAVFQLKNVRRGPGKSGHL 1039
RSO_KEP54849 NPDRPREAY-------NL-FGFGLHKCMGDQFTERTMPAVIKSIFKLKNVRRAPGESGKL 1188
RSO_KEP46854 DPRRPKEHY-------AI-QAIGAHGCPGLDATEQCMAMILREIFKLKNIRRAPGVLGQL 1001
ANI_9R-DOX-AOS KLDRPSNAY--------IHFGYGAHECLGKEIGLTFAVSMLRVLAGLKYLRPAPGDMGML 1108
ATE_9R-DOX-AOS RLDRPASAY--------IQWGYGAHECLGKEIAITFAVSMIRILAGLKYLRPAPGEMGVL 1106
FOX_9S-DOX-AOS NPQRKKEDV--------SAFSYGQHECIAKDVALAFVTGLIKLVADLKELRPAPGQMGTV 1084
CGR_9S-DOX-AOS DAKRKTDPV--------SAFSYGQHECLAKDIATTFIVGLVKLVADLKQLRPAPGQMGLV 1085
MOR_8R-DOX(7,8-LDS) RLDRDLDSY--------TFFGLGPHRCAGDKVVRITMTAVFKVLLQLDGLRRAEGGRGVF 1122
GGR_8R-DOX(7,8-LDS) RLDRPLESY--------VHFGLGPHRCAGEPISQIALSSVMKVLLQLDGLRRAAGPRGEI 1113
ANI_10R-DOX-CYP RLDRPDESY--------LNYGIGSQIGLGKDATLTAVTAMVRAAFSLEGLRPAPGVQGVL 1072
ACL_10R-DOX-CYP RLDRPLDAY--------INHSLGPHGFLSKETSQIALTAMLRAVGRLNNLRRAPGAQGEV 1084
AFU_10R-DOX-CYP RLDRPMNSY--------INPTLGPHGFLSKETSHIALTAMLRAVGRLNNLRVAPGVQGQL 1088
ATE_8R-DOX(5,8-LDS) RLDRDMDLY--------VHFGSGPHKCLGFGLCKLGLTTMLKVVGGLDNLRRAPGPQGQL 1030
ANI_8R-DOX(5,8-LDS) KLDRDMNLY--------AHFGFGPHKCLGLDLCKTGLSTMLKVLGRLDNLRRAPGAQGQL 1039
AFU_8R-DOX(5,8-LDS) KLDRDMNLY--------AHFGFGPHQCLGLGLCKTALTTMLKVIGRLDNLRRAPGGQGKL 1039
FOX_10R-DOX-EAS DPKRPLDKY--------IHYGVGPHACLGRDISQVALTELFRAVFRKKGVRRVPGAQGEL 1068
MOR_10R-DOX-EAS NPRRPAKKY--------IHYGVGPHACLGRDASQIAITEMFRCLFRRRNVRRVPGPQGEL 1119

Figure S13: Partial amino acid sequence alignment of different DOXs from Ascomycota and putative DOXs from the Basidiomycota *C. aegerita* and *Rhizoctonia solani*. A) N-terminal DOX domain containing histidine heme ligands residues as well as the catalytic tyrosine residue shown on a black background, latter being part of the YRWH motif (dark grey background). Leucine and valine residues responsible for the oxygenation at C-10 and C-8 of linoleic acid are underlined with light grey background. B) C-terminal P450 domain containing the NXXQ motif (dark grey background) and the cysteine residue responsible for the coordination of heme iron (black background). Alignment was carried out by using Clustal Omega with default parameters. MOR: *Magnaporthe oryzae*, GGR: *Gaeumannomyces graminis*, ATE: *Aspergillus terreus*, ANID: *Aspergillus nidulans*, AFU: *Aspergillus fumigatus*, FOX: *Fusarium oxysporum*, ANIG; *Aspergillus niger*, ACL: *Aspergillus clavatus*, CGR: *Colletotrichum graminicola*, RSO: *Rhizoctonia solani*. MOR_8R-DOX_7_8-LDS (EHA52010), GGR_8R-DOX_7_8-LDS (AAD49559), ATE_8R-DOX_5_8-LDS (AGA95448), ANID_8R-DOX_5_8-LDS (EAA65132), AFU_8R-DOX_5_8-LDS (EDP50447), FOX_10R-DOX-EAS (EGU86021), MOR_10R-DOX-EAS (EHA53428), ANID_10R-DOX-CYP (AY613780), ACL_10R-DOX-CYP (EAW09782), AFU_10R-DOX-CYP (ABV21633), FOX_9R-DOX (EGU79548, FOXB_09952), CGR_9R-DOX (EFQ36675, GLRG_11821), ANIG_9R-DOX-AOS (EHA25900), ATE_9R-DOX-AOS (AGH14485), FOX_9S-DOX-AOS (EGU88194), CGR_9S-DOX-AOS (EFQ27323).


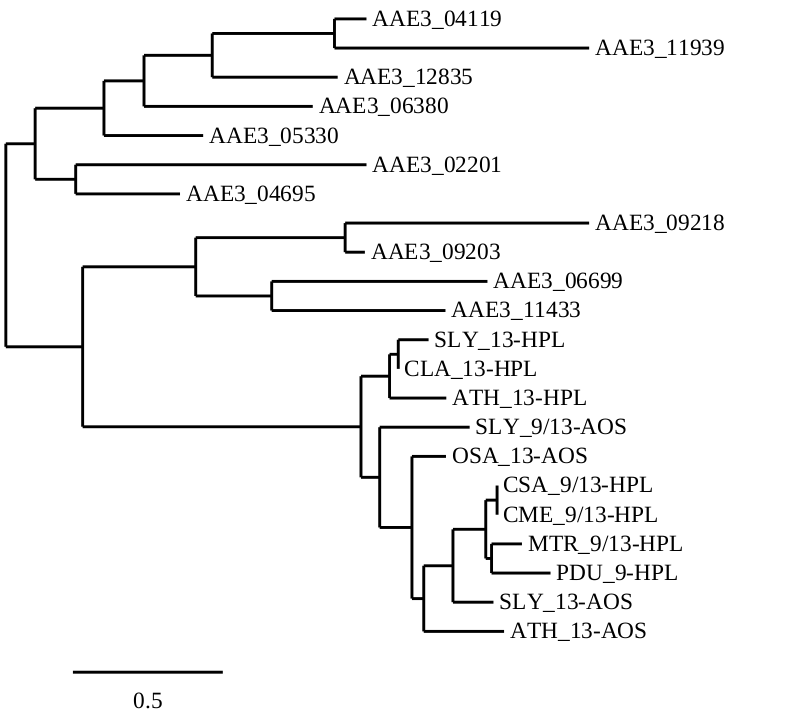


Figure S14: Phylogenetic analysis of different CYP74 proteins of plant and putative HPLs from *C. aegerita*. SLY: *Solanum lycopersicum*, CLA: *Citrullus lanatus*, ATH: *Arabidopsis thaliana*, OSA: *Oryza sativa*, CSA: *Cucumis sativus*, CME: *Cucumis melo*, MTR: *Medicago truncatula*, PDU: *Prunus dulcis*. SLY_13-HPL (K4CF70), CLA_13-HPL (Q66UT1), ATH_13-HPL (Q9ZSY9), SLY_9/13-AOS (Q9LLB0), OSA_13-AOS (Q7XYS3), CSA_9/13-HPL (Q9M5J2, AF229811), CME_9/13-HPL (Q93XR3), MTR_9/13-HPL (Q7X9B3), PDU_9-HPL (Q7XB42, AJ578748), SLY_13-AOS (Q9LLB0), ATH_13-AOS (Q96242).


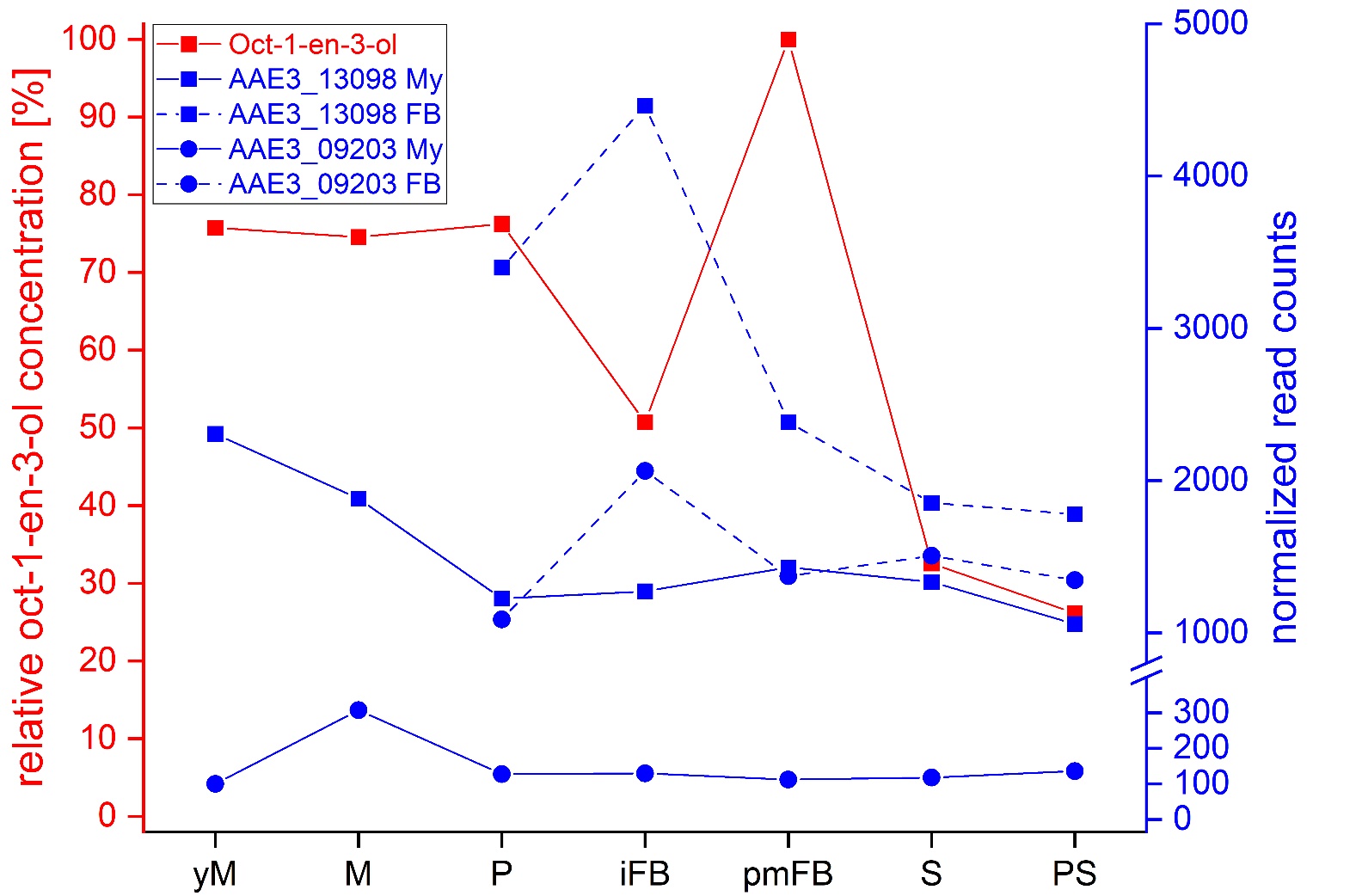


Figure S15: Transcription levels of the putative DOX AAE3_13098 and the putative HPL AAE3_09203 (blue) in the mycelium (My) and in fruiting bodies (FB) during different developmental stages of C. aegerita as well as the relative concentration of oct-1-en-3-ol (red) in the HS of C. aegerita. yM: young (uninduced) mycelium (day 10 post inoculation, p.i.); M: mycelium (day 14 p.i.); P: primordia (day 18 p.i.); iFB: immature fruiting bodies (day 20 p.i.); pmFB: premature fruiting bodies (day 22 p.i.); S: sporulation (day 24 p.i.); PS: post sporulation (day 28 p.i.).


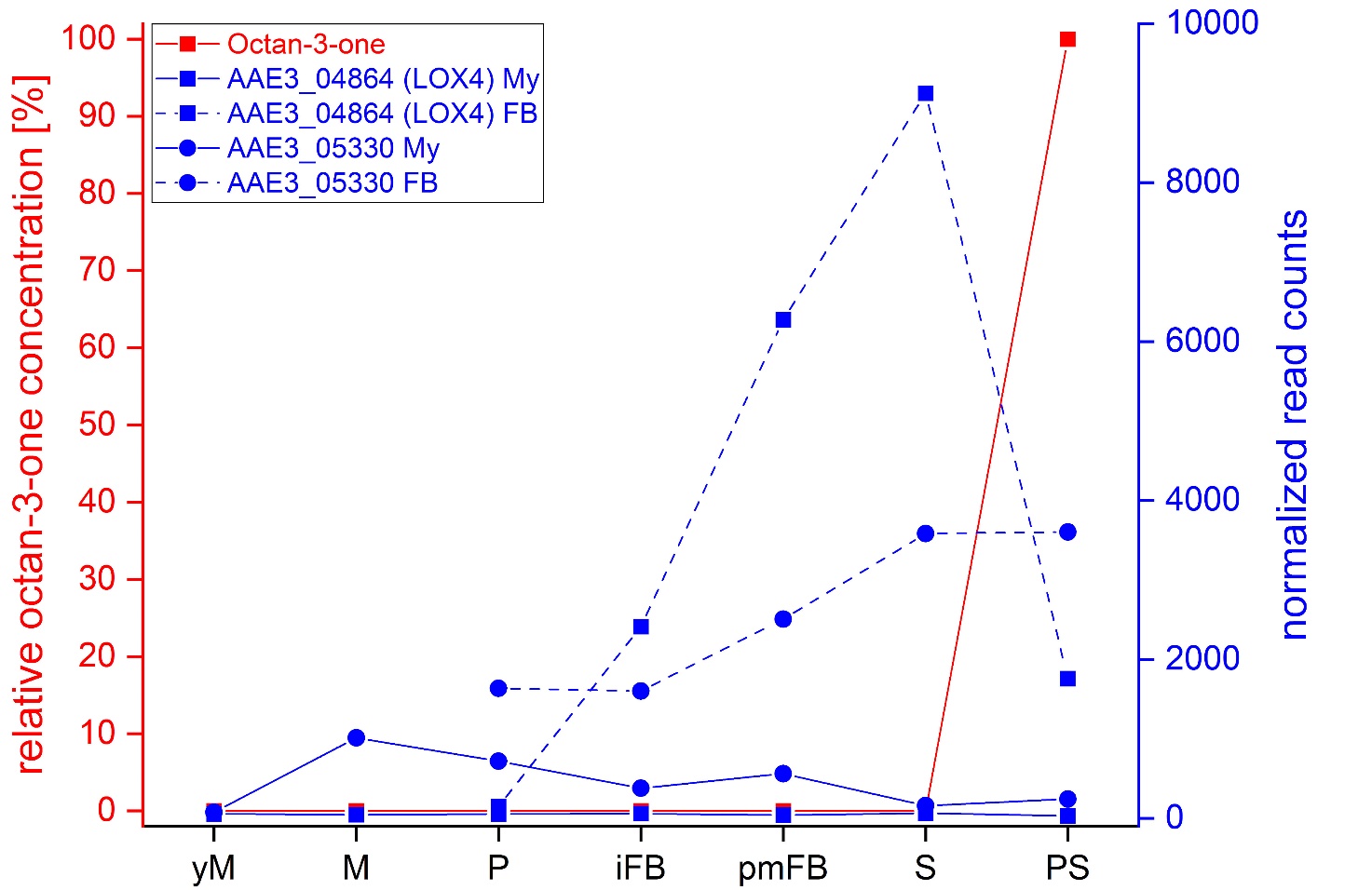


Figure S16: Transcription levels of AAE3_04864 (LOX4) and the putative HPL AAE3_05330 (blue) in the mycelium (My) and in fruiting bodies (FB) during different developmental stages of C. aegerita as well as the relative concentration of octan-3-one (red) in the HS of C. aegerita. yM: young (uninduced) mycelium (day 10 post inoculation, p.i.); M: mycelium (day 14 p.i.); P: primordia (day 18 p.i.); iFB: immature fruiting bodies (day 20 p.i.); pmFB: premature fruiting bodies (day 22 p.i.); S: sporulation (day 24 p.i.); PS: post sporulation (day 28 p.i.).


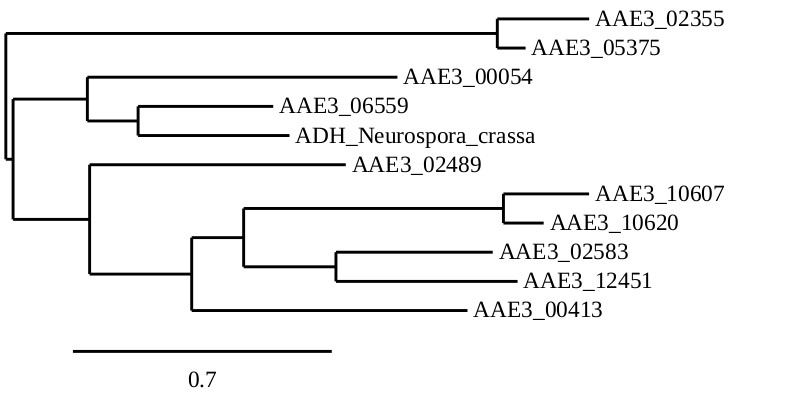


Figure S17: Phylogenetic analysis of putative ADHs from *C. aegerita* and an ADH from *Neurospora crassa* (Q9P6C8).

Sporidiobolus_salmonicolor ------------------------------------------------------------ 0

AAE3_13549 -MAPVTNGRIIFNSIPTGFPVPGETTIYDTTETIDLDTAPLDGGFLLKTLELSVDPYMRG 59

Nicotiana_tabacum MAEEVSNKQVILKNYVTGYPKESDMEIKN--VTIKLKVPEGSNDVVVKNLYLSCDPYMRS 58

Arabidopsis_thaliana --MTATNKQVILKDYVSGFPTESDFDFTT--TTVELRVPEGTNSVLVKNLYLSCDPYMRI 56

Sporidiobolus_salmonicolor ----RPAGTKSYVPPFELGQPIANFGTGEARPVPHLSPDDRQLTALSPQVLKSANASIKQ 56

AAE3_13549 GMRAPEK--KSYSAPFTLGQPLRGYGVGV--------------------VLRSENPQVKA 97

Nicotiana_tabacum RMRKIEG---SYVESFAPGSPITGYGVAK--------------------VLESGDPKFQK 95

Arabidopsis_thaliana RMGKPDPSTAALAQAYTPGQPIQGYGVSR--------------------IIESGHPDYKK 96

: : *.*: .:*.. ::.* . . :

Sporidiobolus_salmonicolor GQHVYGSFPFAEYNVFSKEEASRLRILENKEGLPWTTWVGAAGMPGQTAWHGLRAIGKPQ 116

AAE3_13549 GDHLYGFFEHTHYSIRKDL--TGLQAIENAYNLPWSVFIGVIGMPGKTAYMAWKEYAHPK 155

Nicotiana_tabacum GDLVWGMTGWEEYSIITPT---QTLFKIHDKDVPLSYYTGILGMPGMTAYAGFHEVCSPK 152

Arabidopsis_thaliana GDLLWGIVAWEEYSVITPM--THAHFKIQHTDVPLSYYTGLLGMPGMTAYAGFYEVCSPK 154

*: ::* .*.: . : .:* : : * **** **: . *:

Sporidiobolus_salmonicolor KGETIFVSGAMGAVGQMVISIAHKLGLKVIASAGSDEKVELLKKEFKVEVAFNYKTVDTE 176

AAE3_13549 QGETVFVSTGAGPVGSFVIQLAKADGLKVIASAGSEEKVQFMK-EVGADVAFNYKTTNTA 214

Nicotiana_tabacum KGETVFVSAASGAVGQLVGQFAKMLGCYVVGSAGSKEKVDLLKSKFGFDEAFNYKEEQDL 212

Arabidopsis_thaliana EGETVYVSAASGAVGQLVGQLAKMMGCYVVGSAGSKEKVDLLKTKFGFDDAFNYKEESDL 214

:***::** . * **.:* .:*: * *:.****.***:::* :. : ***** .

Sporidiobolus_salmonicolor KIL----SENPFQIYWDNVAGPTFEAVLNTIEPRGRIIGCVAKQHSNDYNGQPYGIKNIF 232

AAE3_13549 EVLE---KEGPIDIYWDNVGGETLEAALNAANVNARFIECGMISGYN--SGG-APVRNIF 268

Nicotiana_tabacum SAALKRYFPDGIDIYFENVGGKMLDAVLVNMKLYGRIAVCGMISQYN--LEQTEGVHNLF 270

Arabidopsis_thaliana TAALKRCFPNGIDIYFENVGGKMLDAVLVNMNMHGRIAVCGMISQYN--LENQEGVHNLS 272

. ::**::**.* ::*.* : .*: * . * ::*:

Sporidiobolus_salmonicolor QVVSKELLYQGFIVLNHP---IEAFYDEVPKWIASGEVTKPKEHIYKGLDN-GESFNDLF 288

AAE3_13549 HVIGKSITMTGFIVSRIEPKYSAEFYKEVPAKVASGELKY-REHVYNGLEKLGDVILAVQ 327

Nicotiana_tabacum CLITKRIRMEGFLVFDYYHLYPK-YLEMVIPQIKAGKVVY-VEDVAHGLESAPTALVGLF 328

Arabidopsis_thaliana NIIYKRIRIQGFVVSDFYDKYSK-FLEFVLPHIREGKITY-VEDVADGLEKAPEALVGLF 330

:: * : **:* : . * : *:: *.: .**:. : :

Sporidiobolus_salmonicolor TGANFGKAVISLE-- 301

AAE3_13549 KGENKAKAVVHVADD 342

Nicotiana_tabacum SGRNIGKQVVMVSRE 343

Arabidopsis_thaliana HGKNVGKQVVVVARE 345

* * .* *: :

Figure S18: Amino acid sequence alignment of ene-reductases from plants, the fungus *Sporidiobolus salmonicolor,* and the putative ene-reductase AAE3_13549 from *C. aegerita*. *Nicotiana tabacum* (Q9SLN8), *Arabidopsis thaliana* (Q39172), *Sporidiobolus salmonicolor* (A0A0D6ERK8).


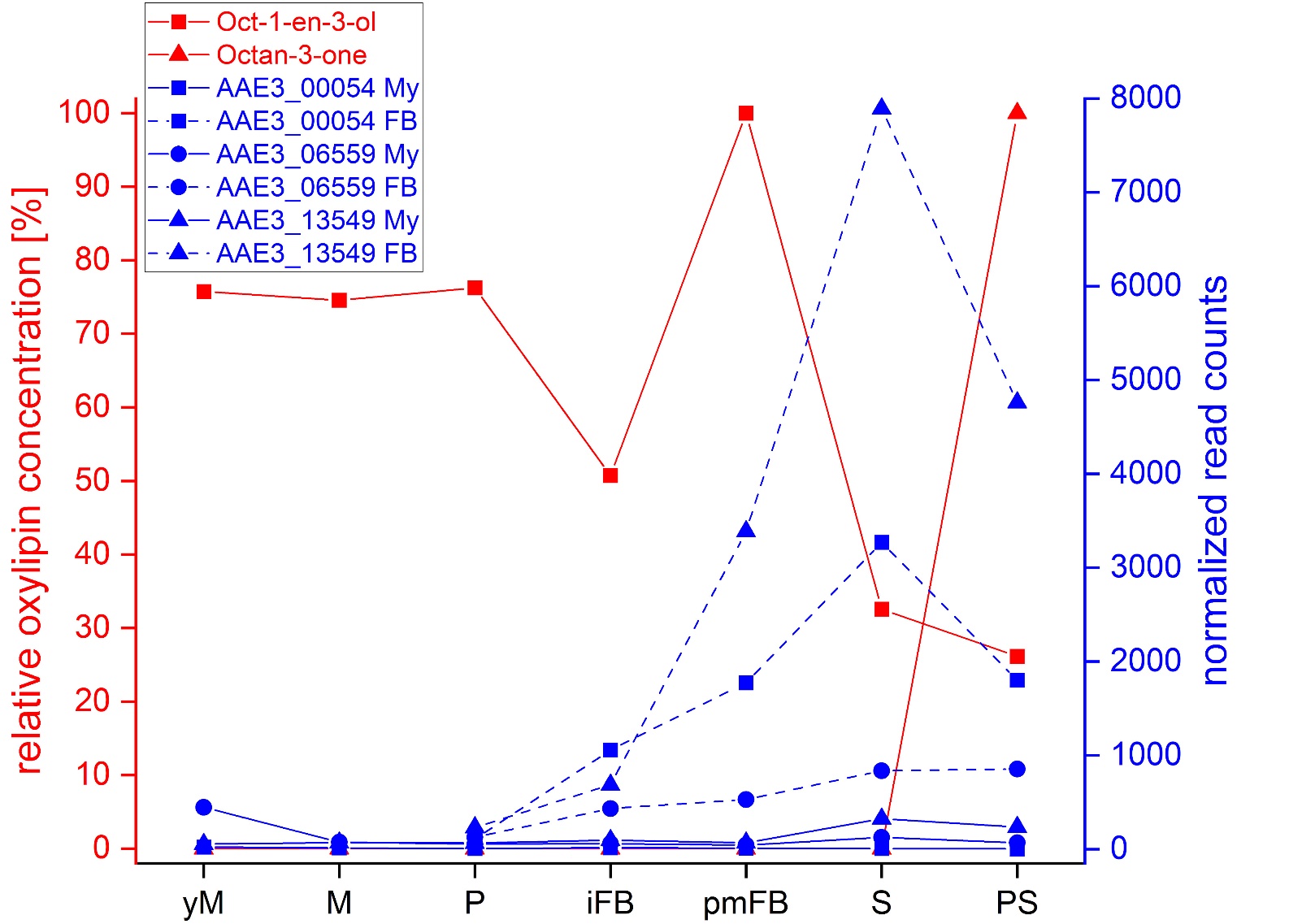


Figure S19: Transcription levels of the putative ADHs AAE3_00054 and AAE3_06559 as well as the putative ene-reductase AAE3_13549 blue in the mycelium (My) and in fruiting bodies (FB) during different developmental stages of C. aegerita as well as the relative concentrations of C8 oxylipins (red) in the HS of C. aegerita. yM: young (uninduced) mycelium (day 10 post inoculation, p.i.); M: mycelium (day 14 p.i.); P: primordia (day 18 p.i.); iFB: immature fruiting bodies (day 20 p.i.); pmFB: premature fruiting bodies (day 22 p.i.); S: sporulation (day 24 p.i.); PS: post sporulation (day 28 p.i.).
